# A general approach for the site-selective modification of native proteins, enabling the generation of stable and functional antibody–drug conjugates†
†Dedicated to Professor Jack Baldwin on the occasion of his 80^th^ birthday.
‡
‡Electronic supplementary information (ESI) available. See DOI: 10.1039/c8sc04645j


**DOI:** 10.1039/c8sc04645j

**Published:** 2018-11-09

**Authors:** Stephen J. Walsh, Soleilmane Omarjee, Warren R. J. D. Galloway, Terence T.-L. Kwan, Hannah F. Sore, Jeremy S. Parker, Marko Hyvönen, Jason S. Carroll, David R. Spring

**Affiliations:** a Department of Chemistry , University of Cambridge , Cambridge , CB2 1EW , UK . Email: spring@ch.cam.ac.uk; b Cancer Research UK Cambridge Institute , University of Cambridge , Cambridge , CB2 0RE , UK . Email: jason.carroll@cruk.cam.ac.uk; c Early Chemical Development , Pharmaceutical Development , IMED Biotech Unit , AstraZeneca , Macclesfield , UK; d Department of Biochemistry , University of Cambridge , Cambridge , CB2 1GA , UK

## Abstract

Antibody–drug conjugates (ADCs) are a class of targeted therapeutics that utilize the specificity of antibodies to selectively deliver highly potent cytotoxins to target cells. Although recent years have witnessed significant interest in ADCs, problems remain with the standard linkage chemistries used for cytotoxin-antibody bioconjugation. These typically (1) generate unstable constructs, which may lead to premature cytotoxin release, (2) often give a wide variance in drug–antibody ratios (DAR) and (3) have poor control of attachment location on the antibody, resulting in a variable pharmacokinetic profile. Herein, we report a novel divinylpyrimidine (DVP) linker platform for selective bioconjugation *via* covalent re-bridging of reduced disulfide bonds on native antibodies. Model studies using the non-engineered trastuzumab antibody validate the utility of this linker platform for the generic generation of highly plasma-stable and functional antibody constructs that incorporate variable biologically relevant payloads (including cytotoxins) in an efficient and site-selective manner with precise control over DAR. DVP linkers were also used to efficiently re-bridge both monomeric and dimeric protein systems, demonstrating their potential utility for general protein modification, protein stabilisation or the development of other protein-conjugate therapeutics.

## Abstract 2

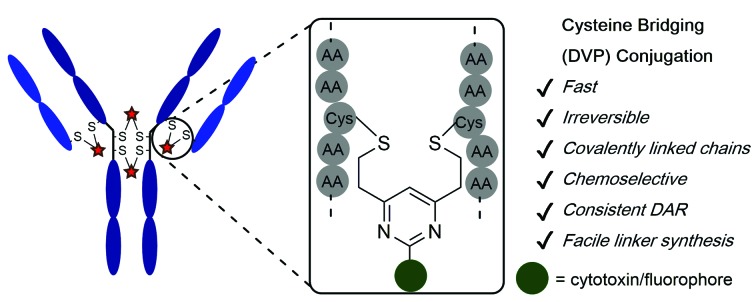
Divinylpyrimidine (DVP) linkers enable access to highly stable and functional antibody–drug conjugates.

## Introduction

**Fig. 1:**
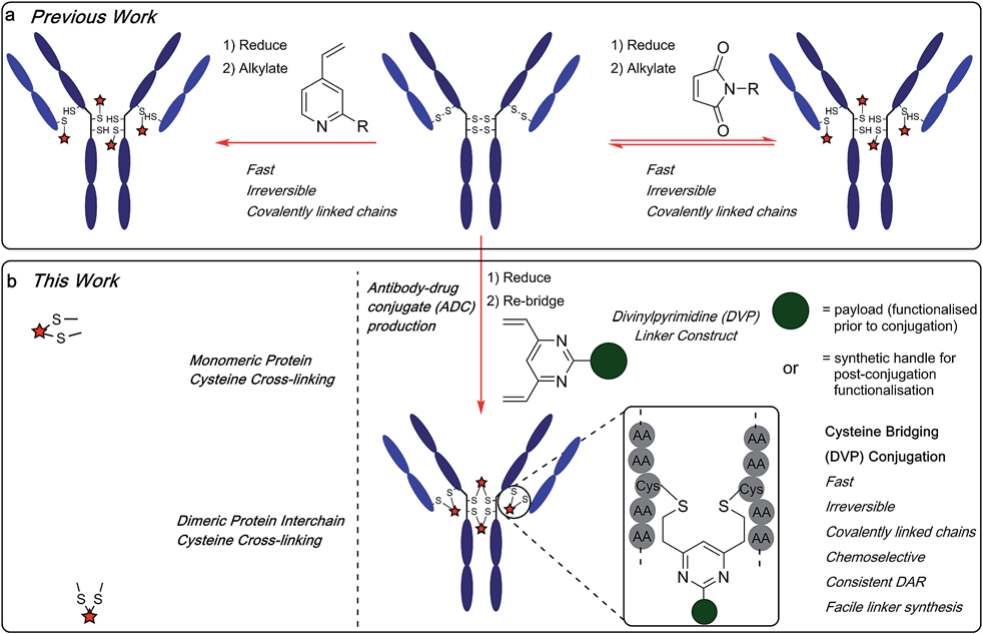
(a) Previous work using monovinylpyridine or maleimide linkers for the generation of ADCs from native antibodies and (b) the divinylpyrimidine (DVP) linkers developed in this work generate homogeneous and stable ADCs *via* cysteine re-bridging (cross-linking).

The emergence of biotherapeutics in recent decades has opened up vast new areas of research for the treatment of a range of grievous diseases,^1^,^2^ with antibody–drug conjugates (ADCs) demonstrating considerable promise as anticancer agents.^3^,^4^ ADCs utilize the impeccable cell-targeting ability of an antibody in combination with the highly potent nature of a cytotoxic payload to achieve cell-selective cytotoxicity^5^ while overcoming the dose-limiting toxicity of classical non-targeted small molecule chemotherapy.^6^,^7^ There are currently four ADCs on the market^8^–^11^ and over 60 other ADCs in clinical trials.^12^ However, current approaches for ADC production still have numerous shortcomings. Stability, drug–antibody ratio (DAR) and drug distribution have all been shown to be crucial to the efficacy, safety and overall pharmacological profile of ADCs and are strongly influenced by the chemistry used to attach the linker to the antibody. Commonly employed nucleophilic bioconjugation at cysteine or lysine residues are pseudorandom: in theory, any of the targeted amino acids within the antibody can be modified. Consequently, there is a lack of selectivity, leading to the formation of ADCs which are heterogeneous in terms of the number of cytotoxin molecules incorporated (the DAR) and their locations on the antibody. Such constructs are associated with unreliable pharmacokinetic profiles and therefore, a reduced pharmacological effect.^13^–^17^ Maleimide conjugation to reduced antibody cysteine thiols has been extensively used in ADC development (Fig. 1a). However, the formed succinimide thioether is inherently unstable in the body (due to a retro-Michael reaction) which leads to premature dissociation of the payload from the antibody.^18^,^19^ The plasma stability of maleimide-based linkers has been increased by hydrolysis of the succinimide thioether ring through linker modifications or antibody engineering.^20^,^21^ However, an inherently stable linker is preferential. The development of new ADC formats to enable site-selective antibody modification, including the incorporation of engineered cysteine residues^13^ and unnatural amino acids^22^,^23^ into the antibody sequence and the use of various enzymatic processes,^24^–^26^ have produced ADCs with precise DAR and defined attachment points. While effective, these methods are complicated and generally inefficient.^27^

Recently, disulfide-bridging linkers have emerged for ADC production: a bis-reactive linker moiety undergoes reaction with both thiol residues derived from a reduced cysteine disulfide bond, leading to covalent re-bridging of the protein. Such linkers are capable of generating ADCs with more precise DAR and drug distribution as well as reforming covalent bonds between the antibody chains. Significant progress has been made in the field with this class of linker by Baker, Caddick and Chudasama and their co-workers, amongst others.^27^–^36^ Despite these impressive advances, new methods are still required for the production of stable and homogeneous ADCs from non-engineered antibodies. We sought to develop a new disulfide bridging linker platform, which generated highly stable ADCs with precise DAR and drug distribution. Vinylpyridines have previously been used to modify proteins *via* cysteine conjugation.^37^ Glythera has recently developed a monovinylpyridine-based linker platform for ADC construction with such conjugates demonstrating excellent stability (Fig. 1a). We envisaged that divinyl-functionalised hetero-aryl linkers could be used to achieve cysteine re-bridging, generating inherently stable constructs whilst also achieving precise control of DAR and site-of-attachment with native antibodies.

Herein, we report upon our investigations in this area and development of a novel divinylpyrimidine (DVP) linker platform for cysteine-bridging bioconjugation with a range of proteins (Fig. 1b). Model studies using the non-engineered trastuzumab antibody validate the utility of this linker platform for the generation of highly plasma-stable antibody constructs that incorporate different biologically relevant payloads in a robust and efficient manner. The utility of the DVP linker platform was also exemplified on other protein systems, exemplifying the capability of such linkers for a range of protein or peptide modification applications.

## Results and discussion

**Fig. 2:**
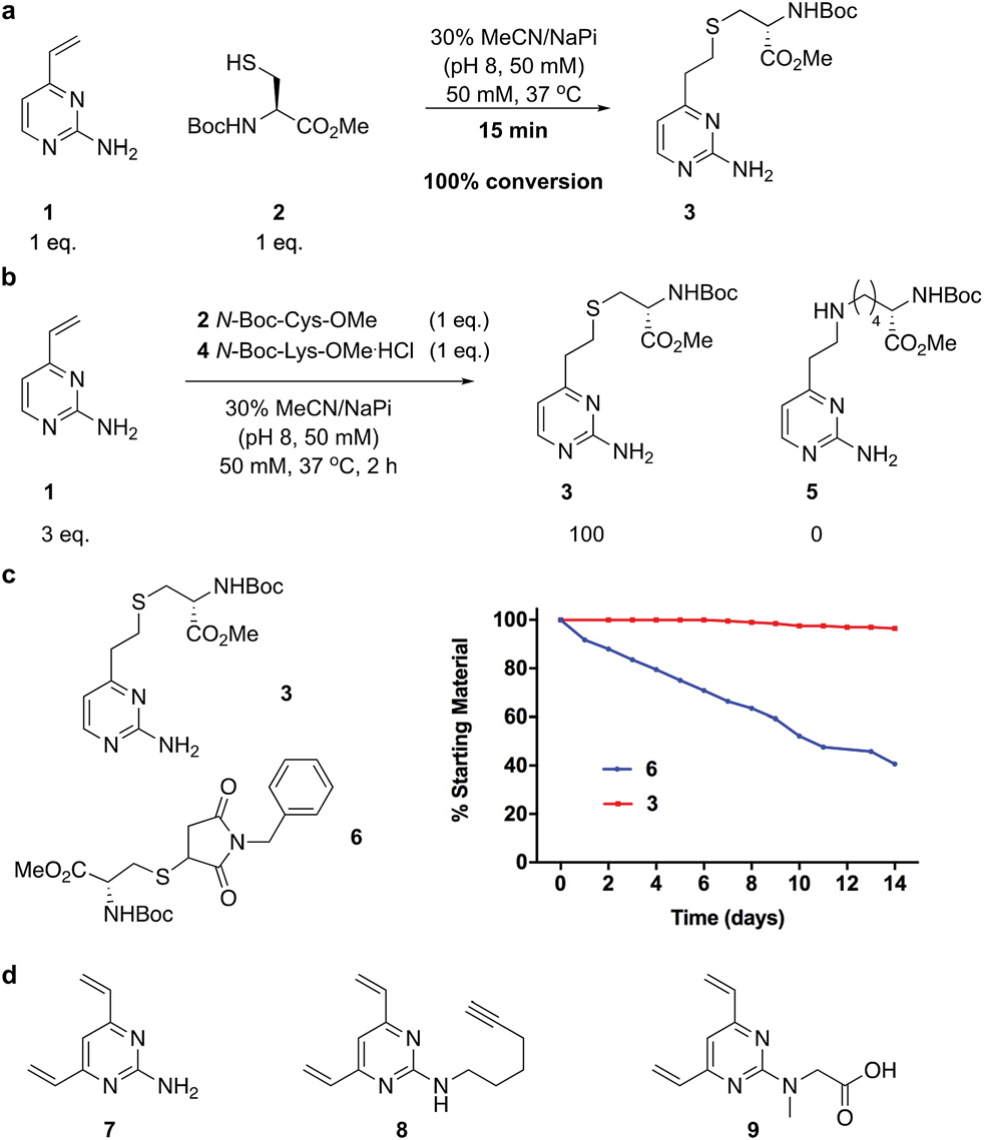
Development and analysis of the DVP linkers. (a) conjugation of vinylpyrimidine **1** with *N*-Boc-Cys-OMe, (b) selectivity experiment by reaction of *N*-Boc-Cys-OMe and *N*-Boc-Lys-OMe with an excess of **1**, (c) stability comparison of vinylpyrimidine-conjugate **3***versus* maleimide conjugate **6** in the presence of reduced GSH and (d) DVP linkers **7**, **8** and **9**.

We began our study by identifying the potential of a vinyl-heteroaryl scaffold for cysteine re-bridging. It was postulated that vinylpyridine bioconjugation would be too slow to enable efficient cross-linking but that replacement of the pyridine with a pyrimidine would enhance the reactivity to desirable levels by increasing the electron accepting capacity of the heteroaryl ring, without compromising the stability seen with vinylpyridine conjugates.^38^ To test this hypothesis, vinylpyrimidine **1** (prepared by a Suzuki–Miyaura cross-coupling, see ESI‡) was reacted with Boc-Cys-OMe in a mixture of aqueous buffer and acetonitrile. Pleasingly, full conversion from **1** to conjugate **3** was achieved in 15 minutes, monitored by thin layer chromatography (TLC), under these bioconjugation compatible conditions (Fig. 2a). A competition experiment involving the reaction of **1** with Boc-Cys-OMe and Boc-Lys-OMe at alkaline pH showed full conversion to the cysteine conjugate **3**. No evidence of the lysine conjugate **5** was observed, even with an excess of vinylpyrimidine **1** present in the reaction (Fig. 2b and ESI Fig. S1‡). Similarly, **1** showed poor reactivity with tris(2-carboxyethyl)phosphine hydrochloride (TCEP), a commonly used reducing agent in cysteine bioconjugation (ESI Fig. S5‡), demonstrating the capability of this scaffold for chemoselective cysteine conjugation. The stability of conjugate **3** under physiological conditions was investigated by incubation with an excess of reduced l-glutathione (GSH) in pH 7.4 buffer at 37 °C. The stability was tracked *via*^1^H NMR and pleasingly, almost no degradation (<5%) was observed after two weeks. In comparison, the corresponding maleimide conjugate **6** showed >50% conversion to the glutathione-maleimide conjugate after two weeks under the same conditions (Fig. 2c and S6‡). This suggested that vinylpyrimidine bioconjugation could indeed be used to generate conjugates that are stable under physiological conditions. Efforts then turned to the synthesis of divinylpyrimidine (DVP) linkers which contained reactive synthetic handles suitable for attachment of other modalities, such as drugs or fluorophores. To this end, DVP linkers **7**, **8** and **9** were synthesised in one, two and three steps, respectively (Fig. 2d, see ESI‡ for synthetic details). The chemoselectivity of linkers **7**, **8** and **9** was then confirmed in an analogous way to monovinylpyrimidine **1** (ESI Fig. S1–S4‡). Subsequently, the stability of Boc-Cys-OMe modified **7**, **8** and **9** in the presence of GSH was also demonstrated *via*^1^H NMR (ESI Fig. S6‡).

**Fig. 3:**
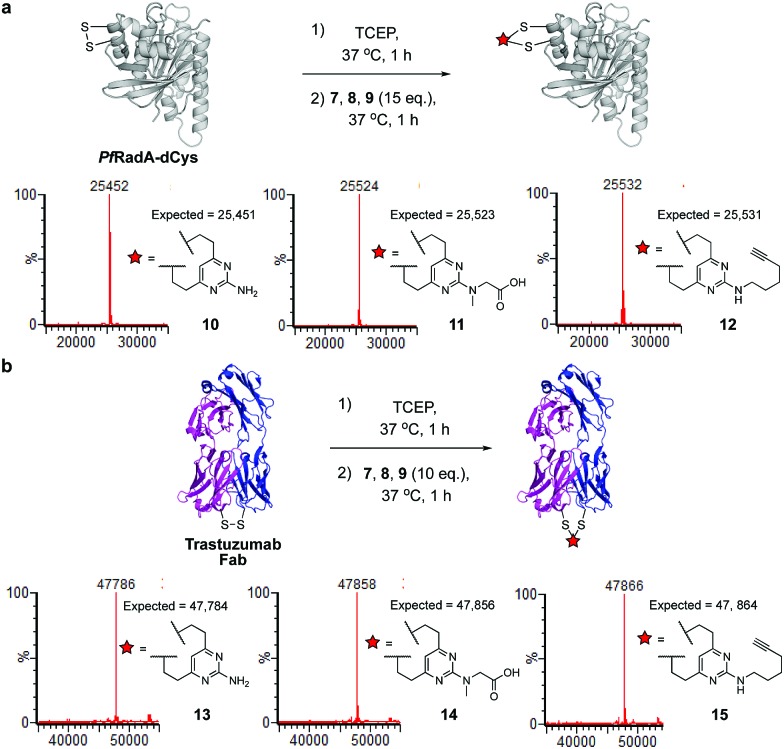
Reaction conditions and subsequent LC-MS analysis for (a) modification of recombinant *Pf*RadA with DVP linkers **7**, **8** and **9** resulted in covalently re-bridged conjugates **10**, **11** and **12**, and (b) bridging of trastuzumab Fab with **7**, **8** and **9** resulted in the desired interchain bridged conjugates **13**, **14** and **15**.

We next sought to ascertain the reactivity of the DVP linkers in protein systems. RadA from *Pyrococcus furiosus* is a DNA recombinase enzyme that does not contain any cysteine residues. Through site-directed mutagenesis, a monomeric version was produced that contained two cysteine residues in close proximity in the tertiary structure (*Pf*RadA-dCys, ESI Fig. S25‡). Pleasingly, reduction of the mutant *Pf*RadA with TCEP followed by the addition of linkers **7**, **8** or **9** (15 equiv.) for 1 hour at 37 °C yielded excellent conversion to the desired covalently re-bridged conjugates **10**, **11** and **12**, as detected by LC-MS (Fig. 3a and ESI Fig. S7–S9‡). To evaluate the intended strategy further, the bridging reaction was appraised in a system where interchain bridging between two polypeptide chains would be required. Antibody Fabs (fragment, antigen binding) are heterodimeric proteins with the chains linked by a single disulfide. Evaluation of the DVP linker platform by reduction of trastuzumab Fab with TCEP, followed by reaction with **7**, **8** or **9** led to complete conversion to the desired interchain-bridged conjugates **13–15** in ∼30 minutes using a slight excess of the linker (10 equiv.) (Fig. 3b and ESI Fig. S10–S13‡). Kinetic analysis of the bridging rate of **8** was conducted with 10 and 20 equivalents linker after TCEP reduction. Strikingly, >90% re-bridging of the Fab chains was observed for both stoichiometries after only 15 minutes (ESI Fig. S14‡). To confirm the selectivity of the DVP linker platform for cysteine residues, **8** was incubated with unreduced trastuzumab Fab and no reaction was observed after two hours at 37 °C (ESI Fig. S23‡). These results exemplify the potential for the DVP linker platform to serve as a general protein modification tool for monomeric and multimeric proteins.

**Fig. 4:**
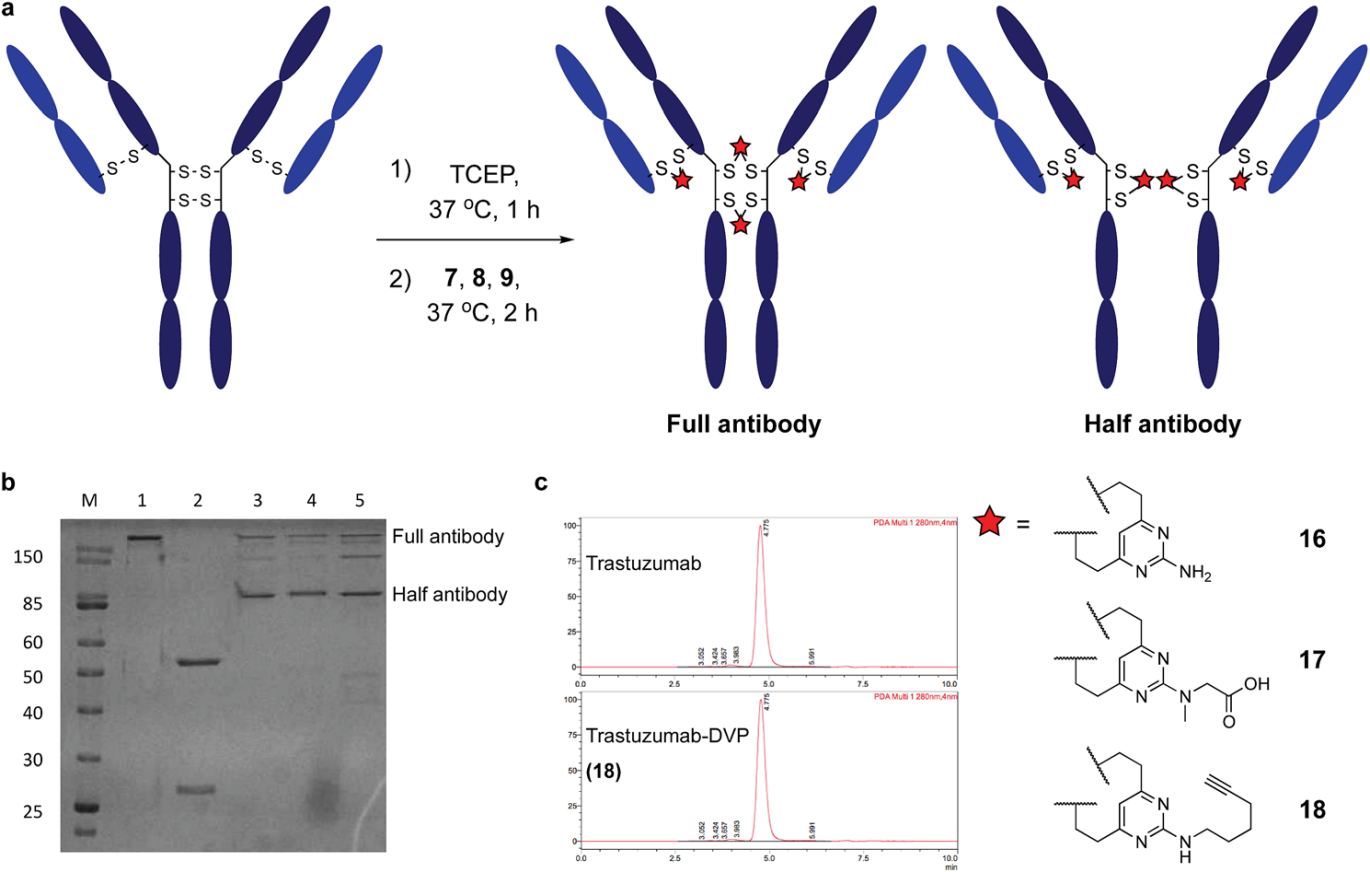
Reaction of trastuzumab with the DVP linkers and subsequent analysis. (a) Cysteine bridging of trastuzumab with **7**, **8** or **9** resulted in re-bridged mAbs **16**, **17** and **18**, (b) analysis of conjugate **16**, **17** and **18** by SDS-PAGE; lane 1 is non-reducing, lanes 2–5 are reducing; lanes: (M) molecular weight marker, (1) trastuzumab, (2) reduced trastuzumab, (3) **16**, (4) **17**, (5) **18**, and (c) SEC analysis of **18**.

Encouraged by earlier results, efforts shifted toward modification of an IgG antibody. DVP linkers potentially enable modification of all four interchain disulfides in an IgG_1_, generating an ADC with definitive modification sites while giving a consistent DAR of four.^39^ Trastuzumab mAb was reduced with TCEP, revealing eight free thiols as evidenced by LC-MS and Ellman's assay (ESI Fig. S15‡). Subsequently, the reduced antibody was treated with a slight excess of **7**, **8** or **9** (10 equiv. per disulfide) for two hours at 37 °C (Fig. 4a). Removal of small molecule reagents by dialysis was followed by LC-MS, SDS-PAGE and RP-HPLC analysis. The fully re-bridged mAbs **16**, **17** and **18** were evident by LC-MS, suggesting good conversion to the desired bioconjugates (ESI Fig. S15, S17 and S18‡). Analysis by SDS-PAGE and RP-HPLC confirmed the presence of the correctly bridged antibodies (Fig. 4b and ESI Fig. S17‡) along with the ‘half-antibody’ formed by intrachain bridging of the hinge region heavy chain cysteines, an issue seen with other re-bridging linkers.^35^,^40^ A large number of conditions (reaction concentration, time, linker stoichiometry) were explored to avoid this ‘half-antibody’ formation, with little change observed (ESI Table S1 and Fig. S16‡). Through this process, it was found that the reaction worked efficiently (90–95% conversion to the bridged bioconjugates) at low concentrations (<10 μM) and with a slight excess of DVP (2.5 equiv. per disulfide). We postulate that the half-antibody conjugate remains useful as the modification site and DAR are still controlled and the stability of the conjugate conferred through the DVP linker remains. Crucially, the light and heavy chains are always re-bridged efficiently, ensuring that the Fab region (the region that confers receptor specificity to the antibody) is covalently linked. In order to determine the effect of bridging on mAb aggregation, antibody conjugate **18** was analysed by size-exclusion chromatography (SEC). This showed identical aggregation levels observed for **18** and the unmodified mAb (Fig. 4c).

**Fig. 5:**
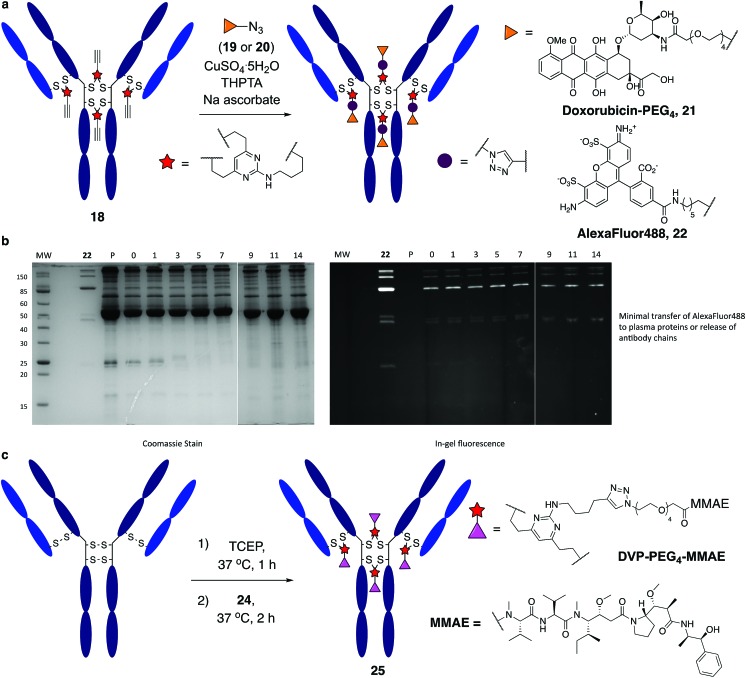
Functional modification and stability analysis of DVP-modified trastuzumab. (a) On-antibody CuAAC reaction between modified trastuzumab, **18** forming a doxorubicin conjugate, **21** and an AlexaFluor™ 488 conjugate, **22**, (b) stability analysis by SDS-PAGE of conjugate **22** in human plasma supplemented with GSH; P = human plasma, MW = molecular weight marker, days of incubation indicated above the representative lane. Left gel is after coomassie staining, right gel is in-gel fluorescence measured before staining, and (c) DVP-mediated formation of an MMAE ADC, **25**.

It was anticipated that the synthetic handles present in DVP linkers would enable modular and divergent functionalisation, both before and after DVP antibody conjugation. Terminal alkyne-functionalised DVP **8** was identified as a suitable candidate for post-conjugation modification, as copper-catalysed azide-alkyne cycloaddition (CuAAC) chemistry offers a well-established method for diverse functionalisation under physiologically relevant conditions.^41^ An azide-functionalised doxorubicin (**19**) was synthesised and reacted with **18** in the presence of CuSO_4_·5H_2_O, tris(3-hydroxypropyltriazolylmethyl)amine (THPTA) and sodium ascorbate (Fig. 5a). Gratifyingly, excellent conversion to triazole product **21** was observed by LC-MS. DAR analysis by UV-vis spectroscopy also confirmed high conversion with a measured DAR of 4.0 (ESI Fig. S19‡). With the aim of creating a potentially valuable cellular imaging agent, conjugate **18** was reacted with AlexaFluor™ 488 azide **20** under CuAAC conditions (Fig. 5a). Similarly, excellent conversion was observed with a measured fluorophore-antibody ratio (FAR) of 3.9 (ESI Fig. S20‡).

Synthesis of **22** enabled assessment of the plasma stability of DVP bioconjugates. The fluorescent antibody conjugate was incubated in human plasma supplemented with GSH at 37 °C for two weeks. Pleasingly, in-gel fluorescence and coomassie staining revealed almost no transfer of the AlexaFluor™ 488 label onto plasma proteins, or release of individual heavy or light chains from the antibody (Fig. 5b and ESI Fig. 22‡). This analysis confirms the stability assessment made on the small molecule model system that DVP linkers enable the generation of highly stable bioconjugates.

Functionalisation before antibody conjugation was then examined using the highly potent dolastatin 10 analogue, monomethylauristatin E (MMAE) as a representative payload. A DVP–PEG_4_–MMAE linker-warhead, **24** was prepared from **8** and azide-functionalised MMAE (**23**) (see ESI‡). Reaction of reduced trastuzumab with **24** (10 equiv. per disulfide) proceeded with excellent conversion to **25***via* LC-MS (Fig. 5c and ESI Fig. S21‡).

**Fig. 6:**
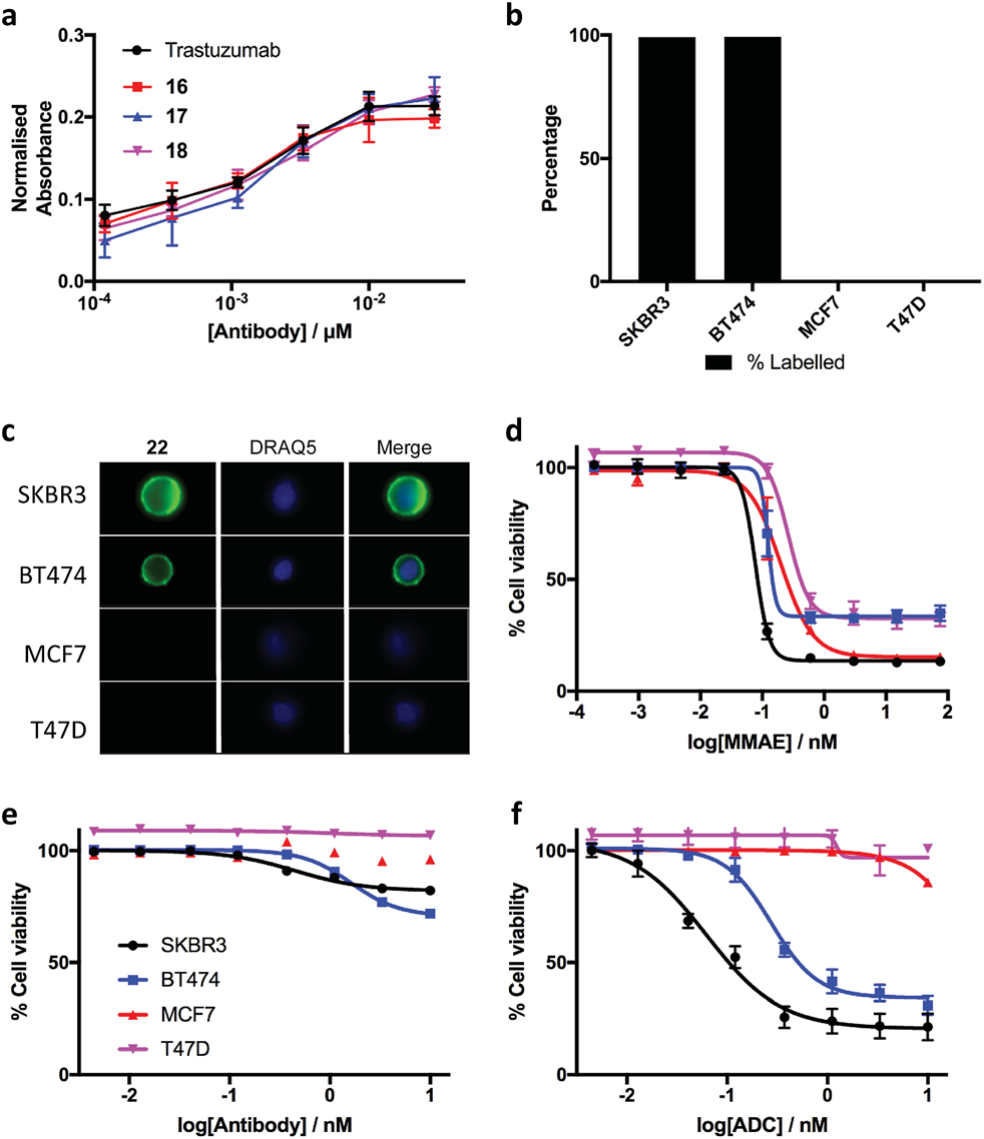
Biological evaluation of the DVP linker platform. (a) Binding affinity comparison of trastuzumab, **16**, **17** and **18***via* ELISA. Error bars represent the standard deviation of biological quadruplicates, (b) percentage labelling of HER2-positive and HER2-negative cells with **22**, (c) internalisation of **22** in HER2-positive cells without any observed internalisation in HER2-negative cells, and cytotoxicity in HER2-positive and HER2-negative cells with (d) MMAE, (e) trastuzumab and (f) DVP-MMAE ADC **25**. Viability data shows the mean of three independent experiments and error bars represent s.e.m.

The biological effects of DVP-bridging were then investigated. Firstly, trastuzumab conjugates, **16–18** all demonstrated comparable affinities to the native antibody for the HER2 receptor *via* enzyme-linked immunosorbent assay (ELISA) (Fig. 6a). Fluorescence-activated cell sorting (FACS) coupled with live individual cell imaging was next used to ensure that the DVP conjugation did not alter cellular recognition and selectivity. Fluorescent trastuzumab conjugate, **22** was incubated in both HER2-positive (SKBR3 and BT474) and HER2-negative (MCF7 and T47D) breast cancer cell lines. The cells were incubated at 37 °C for 1 hour to allow antigen binding and complex internalisation, followed by washing with PBS to remove any unbound antibody. Subsequent FACS analysis revealed full labelling of both HER2-positive cell lines while only minor labelling was observed with HER2-negative cell lines (Fig. 6b). Internalisation of the conjugate was observed in both HER2-positive cell types, with no internalisation visible in either HER2-negative cell line (Fig. 6c), confirming that DVP bridging does not affect receptor specificity, affinity or complex internalisation.

Finally, evaluation of the *in vitro* cytotoxicity of a therapeutically relevant ADC was undertaken. DVP-MMAE ADC **25**, containing a non-cleavable linker was used to treat both HER2-positive (SKBR3 and BT474) and HER2-negative (MCF7 and T47D) cell lines. Cytotoxicity was only observed in the HER2-positive cell lines (Fig. 6f and ESI Fig. S24‡), demonstrating that DVP linkers do not affect the cell-killing ability of MMAE, enabling the use of these linkers for the delivery of auristatin payloads with non-cleavable linkers. In contrast to the specific cytotoxicity observed with our ADC, treatment of the same cell lines with free MMAE resulted in high levels of cytotoxicity in both HER2-positive and HER2-negative cell lines (Fig. 6d). Furthermore, incubation of unmodified trastuzumab with these cell lines did not cause significant cytotoxicity (Fig. 6e).

## Conclusion

In conclusion, we have developed a novel DVP linker platform for bioconjugation through the covalent re-bridging of cysteine residues generated by the reduction of native disulfide bonds. Model studies using the trastuzumab antibody validate the utility of this linker platform for the efficient generation of highly plasma-stable antibody constructs from non-engineered antibodies. We have shown that this technology can be used to introduce different biologically relevant payloads, in an efficient and site-selective manner with control over DAR, either before or after bioconjugation, without recourse to any other additional processes. Furthermore, the conjugation platform utilizes a reaction that is orthogonal to other biocompatible reactions (such as the CuAAC), enabling their use in tandem with one another. The modified antibodies maintained their cellular specificity and receptor affinity, and a DVP-ADC demonstrated exquisite potency and selectivity for its target cells, while having little effect on the mechanism of action of its auristatin payload. In addition, the DVP linkers showed excellent cysteine re-bridging of monomeric and dimeric proteins, enabling their use for general protein modification,^42^ protein stabilisation^43^,^44^ or the development of other biotherapeutics such as stapled peptides.^45^,^46^

## Conflicts of interest

There are no conflicts to declare.

## Supplementary Material

Supplementary information
