# Correction: A general approach for the site-selective modification of native proteins, enabling the generation of stable and functional antibody–drug conjugates

**DOI:** 10.1039/c8sc90248h

**Published:** 2018-12-13

**Authors:** Stephen J. Walsh, Soleilmane Omarjee, Warren R. J. D. Galloway, Terence T.-L. Kwan, Hannah F. Sore, Jeremy S. Parker, Marko Hyvönen, Jason S. Carroll, David R. Spring

**Affiliations:** a Department of Chemistry , University of Cambridge , Cambridge , CB2 1EW , UK . Email: spring@ch.cam.ac.uk; b Cancer Research UK Cambridge Institute , University of Cambridge , Cambridge , CB2 0RE , UK . Email: jason.carroll@cruk.cam.ac.uk; c Early Chemical Development , Pharmaceutical Development , IMED Biotech Unit , AstraZeneca , Macclesfield , UK; d Department of Biochemistry , University of Cambridge , Cambridge , CB2 1GA , UK

## Abstract

Correction for ‘A general approach for the site-selective modification of native proteins, enabling the generation of stable and functional antibody–drug conjugates’ by Stephen J. Walsh *et al.*, *Chem. Sci.*, 2019, DOI: ; 10.1039/c8sc04645j.



## 

The authors regret that Fig. 1 is incorrect in the original manuscript. Structures, ticks and crosses were absent. The correct figure is displayed below.

**Fig. 1:**
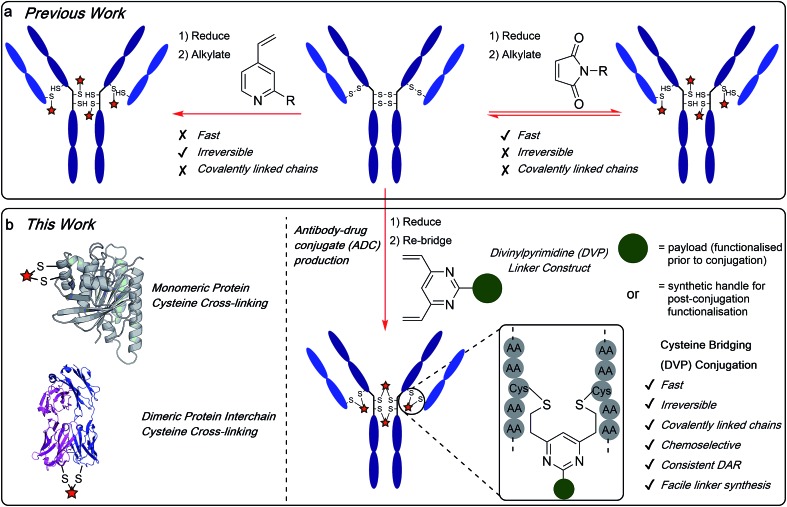
(a) Previous work using monovinylpyridine or maleimide linkers for the generation of ADCs from native antibodies and (b) the divinylpyrimidine (DVP) linkers developed in this work generate homogeneous and stable ADCs via cysteine re-bridging (crosslinking).

The Royal Society of Chemistry apologises for these errors and any consequent inconvenience to authors and readers.

