# Supplementary material for: A general approach for the site-selective modification of native proteins, enabling the generation of stable and functional antibody–drug conjugates
Source: Chem Sci. 2018 Nov 9;10(3):694–700. doi: 10.1039/c8sc04645j (PMC6349026; doi:10.1039/c8sc04645j)

Electronic Supplementary Material (ESI) for Chemical Science.
This journal is © The Royal Society of Chemistry 2018

A General Approach for the Site-Selective Modification of Native Proteins, Enabling the Generation of Stable and Functional Antibody-Drug Conjugates

Stephen J. Walsh[1], Soleilmane Omarjee[2], Warren R. J. D. Galloway[1], Terence T.-L. Kwan[1], Hannah F. Sore[1], Jeremy S. Parker[3], Marko Hyvönen[4], Jason S. Carroll[2]*, David R. Spring[1]*

[1]Department of Chemistry, University of Cambridge, Lensfield Rd, Cambridge, CB2 1EW, UK. [2]Cancer Research UK Cambridge Institute, University of Cambridge, Robinson Way, Cambridge, CB2 0RE, UK. [3]Early Chemical Development, Pharmaceutical Sciences, IMED Biotech Unit, AstraZeneca, Macclesfield, UK. [4]Department of Biochemistry, University of Cambridge, Tennis Court Rd, CB2 1GA, UK.

Supplementary Information

Contents

General Experimental Details 3
Chemical Synthesis 5
Cysteine/Lysine Selectivity 13
TCEP Reactivity 17
Stability Analysis 18
*Pf*RadA-dCys Preparation 21
*Pf*RadA-dCys **7** Conjugation 22
*Pf*RadA-dCys **8** Conjugation 24
*Pf*RadA-dCys **9** Conjugation 24
Antibody Conjugation 25
Fab Preparation 25
Trastuzumab Fab **7** conjugation 26
Trastuzumab Fab **8** conjugation 27
Trastuzumab Fab **9** conjugation 28
Trastuzumab Fab 8 conjugation kinetic experiment 29
Trastuzumab **7** conjugation 29
Trastuzumab **7** conjugation optimisation 31
Trastuzumab **8** conjugation 33
Trastuzumab **9** conjugation 34
Trastuzumab-DVP (**18**) Dox-PEG$_4$-N$_3$ CuAAC 35
UV-vis DAR Calculation 36
Trastuzumab-DVP (**18**) AlexaFluor488 Azide CuAAC 37
UV-vis FAR Calculation 38
Trastuzumab **24** conjugation 39
Trastuzumab Ellman's Test 40
Trastuzumab-DVP **18** Stability 40
Cysteine selectivity 41
Enzyme-linked immunosorbent assay (ELISA) 42
Cells Lines 42
Live Cell Labeling/Internalization by Fluorescence-activated cell sorting (FACS) 43
Cell Viability 43
Cell Growth Assay 44
*Pf*RadA Sequence and SDS-PAGE 45
NMR Spectra 47
HPLC 59

General Experimental Details

All solvents and reagents were used as received unless otherwise stated. Ethyl acetate, methanol, dichloromethane, acetonitrile and toluene were distilled from calcium hydride. Diethyl ether was distilled from a mixture of lithium aluminium hydride and calcium hydride. Petroleum ether (PE) refers to the fraction between 40 – 60 °C upon distillation. Tetrahydrofuran was dried using Na wire and distilled from a mixture of lithium aluminium hydride and calcium hydride with triphenylmethane as indicator.

Non-aqueous reactions were conducted under a stream of dry nitrogen using oven-dried glassware. Temperatures of 0 °C were maintained using an ice-water bath. Room temperature (rt) refers to ambient temperature.

Yields refer to spectroscopically and chromatographically pure compounds unless otherwise stated. Reactions were monitored by thin layer chromatography (TLC) or liquid chromatography mass spectroscopy (LC-MS). TLC was performed using glass plates pre-coated with Merck silica gel 60 F_{254} and visualized by quenching of UV fluorescence (λ_{max} = 254 nm) or by staining with potassium permanganate. Retention factors (R_f) are quoted to 0.01. LC-MS was carried out using a Waters ACQUITY H-Class UPLC with an ESCi Multi-Mode Ionisation Waters SQ Detector 2 spectrometer using MassLynx 4.1 software; EI refers to the electrospray ionisation technique; LC system: solvent A: 2 mM NH_4OAc in H_2O/MeCN (95:5); solvent B: MeCN; solvent C: 2% formic acid; column: ACQUITY UPLC® CSH C18 (2.1 mm × 50 mm, 1.7 µm, 130 Å) at 40 °C; gradient: 5 – 95 % B with constant 5 % C over 1 min at flow rate of 0.6 mL/min; detector: PDA eλ Detector 220 – 800 nm, interval 1.2 nm.

Flash column chromatography was carried out using slurry-packed Merck 9385 Kieselgel 60 SiO_2 (230-400 mesh) under a positive pressure of nitrogen.

Analytical high performance liquid chromatography (HPLC) was performed on Agilent 1260 Infinity machine, using a Supelcosil™ ABZ+PLUS column (150 mm × 4.6

mm, 3 μm) with a linear gradient system (solvent A: 0.05% (v/v) TFA in H_2O; solvent B: 0.05% (v/v) TFA in MeCN) over 20 min at a flow rate of 1 mL/min, and UV detection (λ_{max} = 220 – 254 nm).

Melting points (m.p.) were obtained using a Büchi Melting Point B-545 melting point apparatus and are uncorrected.

Infrared (IR) spectra were recorded neat on a Perkin-Elmer Spectrum One spectrometer with internal referencing. Selected absorption maxima (ν_{max}) are reported in wavenumbers (cm^{-1}).

Proton and carbon nuclear magnetic resonance (NMR) were recorded using an internal deuterium lock on Bruker DPX-400 (400 MHz, 101 MHz), Bruker Avance 400 QNP (400 MHz, 101 MHz) and Bruker Avance 500 Cryo Ultrashield (500 MHz, 126 MHz). Tetramethylsilane was used as an internal standard. In proton NMR, chemical shifts (δ_H) are reported in parts per million (ppm), to the nearest 0.01 ppm and are referenced to the residual non-deuterated solvent peak ($CDCl_3$: 7.26, DMSO-d_6: 2.50, CD_3OD: 3.31, D_2O: 4.79). Coupling constants (*J*) are reported in Hertz (Hz) to the nearest 0.1 Hz. Data are reported as follows: chemical shift, multiplicity (s = singlet; d = doublet; t = triplet; q = quartet; qn = quintet; sep = septet; m = multiplet; or as a combination of these, e.g. dd, dt etc.), integration and coupling constant(s). In carbon NMR, chemical shifts (δ_C) are quoted in ppm, to the nearest 0.1 ppm, and are referenced to the residual non-deuterated solvent peak ($CDCl_3$: 77.16, DMSO-d_6, 39.52, CD_3OD: 49.00).

High resolution mass spectrometry (HRMS) measurements were recorded with a Micromass Q-TOF mass spectrometer or a Waters LCT Premier Time of Flight mass spectrometer. Mass values are reported within the error limits of ±5 ppm mass units. ESI refers to the electrospray ionisation technique.

Protein LC–MS was performed on a Xevo G2-S TOF mass spectrometer coupled to an Acquity UPLC system using an Acquity UPLC BEH300 C4 column (1.7 μm, 2.1 × 50

mm). H_2O with 0.1% formic acid (solvent A) and 95% MeCN and 5% water with 0.1% formic acid (solvent B), were used as the mobile phase at a flow rate of 0.2 mL/min. The gradient was programmed as follows: 95% A for 0.93 min, then a gradient to 100% B over 4.28 min, then 100% B for 1.04 minutes, then a gradient to 95% A over 1.04 min. The electrospray source was operated with a capillary voltage of 2.0 kV and a cone voltage of 40 V. Nitrogen was used as the desolvation gas at a total flow of 850 L/h. Total mass spectra were reconstructed from the ion series using the MaxEnt algorithm preinstalled on MassLynx software (v4.1 from Waters) according to the manufacturer's instructions. Trastuzumab samples were deglycosylated with PNGase F (New England Biolabs) prior to LC-MS analysis.

Chemical Synthesis

2-amino-4-vinyl-pyrimidine (1)

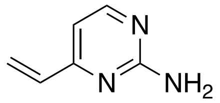

A solution of 2-amino-4-chloropyrimidine (300 mg, 2.32 mmol), potassium vinyltrifluoroborate (931 mg, 6.95 mmol), $Pd(dppf)Cl_2{\cdot}CH_2Cl_2$ (189 mg, 0.232 mmol) and potassium carbonate (1.92 g, 13.9 mmol) in THF/H_2O (10:1, 7.7 mL) was heated to 70 °C for 16 h. Upon completion, the reaction mixture was filtered through Celite® and the solvent removed *in vacuo*. The resulting residue was purified by flash column chromatography (FCC, 40% EtOAc/PE) to yield 2-amino-4-vinylpyrimidine **1** (195 mg, 1.61 mmol, 70%) as a white solid. $\mathbf{R_f}$ 0.12 (SiO_2; 40% EtOAc/PE); **m.p.** 83-86 °C; $\boldsymbol{\nu_{max}}$ (neat/cm^{-1}) 3318, 1648, 1546, 1466, 1403; $\boldsymbol{\delta_H}$ (400 MHz, $CDCl_3$) 8.26 (d, 1H, *J* = 5.2 Hz), 6.65 (d, 1H, *J* = 5.2 Hz), 6.58 (dd, 1H, *J* = 17.4, 10.6 Hz), 6.37 (dd, 1H, *J* = 17.5, 1.3 Hz), 5.64 (dd, 1H, *J* = 10.7, 1.2 Hz), 5.20 (s, 2H); $\boldsymbol{\delta_C}$ (101 MHz, $CDCl_3$) 163.9, 162.7, 158.4, 135.4, 123.0, 108.9; **HRMS** (ESI) *m/z* found $[M+H]^+$ 122.0715, $C_6H_8N_3^+$ required 122.0713.

Methyl *S*-(2-(2-aminopyrimidin-4-yl)ethyl)-*N*-*tert*-butoxycarbonyl)-*L*-cysteinate (3)

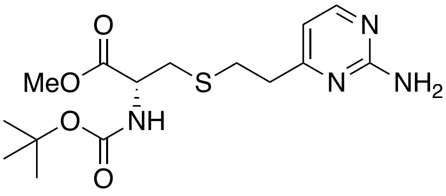

A solution of **1** (15.0 mg, 124 µmol), *N*-(*tert*-butoxycarbonyl)-*L*-cysteine methyl ester (29.2 mg, 124 µmol) in 30% MeCN/sodium phosphate buffer (pH 8, 50 mM, 2.48 mL) was stirred at 37 °C for 15 min. The reaction was analyzed every 2 min by TLC and upon completion, the mixture was diluted with H_2O (10 mL), extracted with EtOAc (4 × 10 mL), dried ($MgSO_4$) and concentrated *in vacuo*. The crude residue was purified by FCC (50-100% EtOAc/PE) to yield **3** (39.6 mg, 111 µmol, 90 %) as a clear oil. $\mathbf{R_f}$ 0.10 (SiO_2; 50% EtOAc/PE); $\mathbf{\nu_{max}}$ (neat/cm^{-1}) 3334, 2977, 1741, 1704, 1616, 1562, 1457; $\mathbf{\delta_H}$ (400 MHz, $CDCl_3$) 8.17 (d, 1H, *J* = 5.1 Hz), 6.49 (d, 1H, *J* = 5.5 Hz), 5.70 (d, 1H, *J* = 7.5 Hz), 5.13 (s, 2H), 4.60-4.56 (m, 1H), 3.74 (s, 3H), 3.03-2.96 (m, 2H), 2.95-2.81 (m, 4H), 1.44 (s, 9H); $\mathbf{\delta_C}$ (101 MHz, $CDCl_3$) 171.7, 169.3, 163.1, 158.3, 155.4, 111.0, 80.4, 53.7, 52.7, 37.5, 34.7, 30.8, 28.5; **HRMS** (ESI) *m/z* found $[M+H]^+$ 357.1606, $C_{15}H_{25}N_4O_4{}^{32}S_1{}^+$ required 357.1597.

Methyl *S*-(1-benzyl-2,5-dioxopyrrolidin-3-yl)-*N*-(*tert*-butoxycarbonyl)-*L*-cysteinate (6)

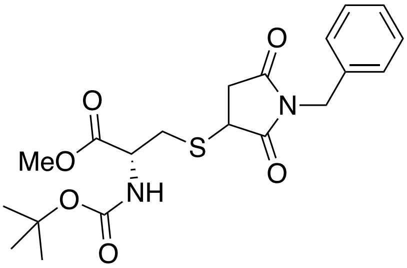

A solution of *N*-benzylmaleimide (100 mg, 0.534 mmol), *N*-(*tert*-butoxycarbonyl)-*L*-cysteine methyl ester (151 mg, 0.641 mmol) and triethylamine (74.0 µL, 0.534 mmol) was stirred at 37 °C for 16 h. Upon completion, the reaction mixture was concentrated *in vacuo* and the crude residue purified by FCC (35% EtOAc/PE) to yield **6** (210 mg, 0.497 mmol, 93%) as a white solid. $\mathbf{R_f}$ 0.16 (SiO_2; 35% EtOAc/PE); **m.p.** 73-77 °C; $\mathbf{\nu_{max}}$ (neat/cm^{-1}) 2982, 1744, 1700, 1498, 1434; $\mathbf{\delta_H}$ (400 MHz, DMSO-d_6) 7.39-7.23 (m, 5H), 4.56 (s, 2H), 4.29-4.21 (m, 1H), 4.11-4.07 (m, 1H), 3.64 (s, 3H), 3.28-

3.21 (m, 2H), 3.13-3.00 (m, 1H), 2.60-2.52 (m, 1H) 1.38 (s, 9H); **δ_C** (101 MHz, DMSO-*d*$_6$) 176.7, 174.9, 171.4, 155.5, 136.0, 128.6, 127.5, 127.4, 78.6, 53.5, 52.2, 41.6, 39.0, 35.9, 32.5, 28.2; **HRMS** (ESI) *m/z* found $[M+H]^+$ 423.1576, $C_{20}H_{27}N_2O_6{}^{32}S_1{}^+$ required 423.1590.

2-amino-4,6-divinylpyrimidine (7)

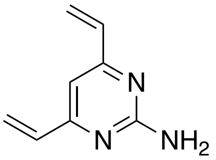

2-amino-4,6-dichloropyrimidine (200 mg, 1.22 mmol), potassium vinyltrifluoroborate (490 mg, 3.66 mmol), Pd(dppf)Cl_2·CH_2Cl_2 (100 mg, 0.122 mmol) and potassium carbonate (1.01 g, 7.32 mmol) in THF/H_2O (10:1, 3.3 mL) were heated to 70 °C for 18 h. Upon completion, the reaction mixture was filtered through Celite® and the solvent removed *in vacuo*. The resulting residue was purified by FCC (20-40% EtOAc/PE) to yield 2-amino-4,6-divinylpyrimidine **7** (152 mg, 1.03 mmol, 85%) as an off-white solid. **R_f** 0.29 (SiO_2; 50% EtOAc/PE); **m.p.** 81-83 °C; **ν_{max}** (neat/cm^{-1}) 3213, 1567, 1536, 1415; **δ_H** (500 MHz, DMSO-*d*$_6$) 6.81 (s, 1H), 6.57 (dd, 2H, *J* = 17.4, 10.6 Hz), 6.54 (s, 2H), 6.32 (dd, 2H, *J* = 17.5, 1.5 Hz), 5.58 (dd, 2H, *J* = 10.7, 1.4 Hz); **δ_C** (126 MHz, DMSO-*d*$_6$) 163.6, 163.4, 136.1, 121.5, 104.7; **HRMS** (ESI) *m/z* found $[M+H]^+$ 148.0871, $C_8H_{10}N_3{}^+$ required 148.0869.

***N*-(hex-5-yn-1-yl)-4,6-divinylpyrimidin-2-amine (8)**

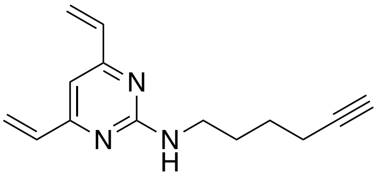

To a solution of amine **7** (50.0 mg, 0.340 mmol) in DMF (1.5 mL) was added 6-iodo-1-hexyne (223 µL, 1.69 mmol) followed by the slow addition of sodium hydride (60% in mineral oil, 68.0 mg, 1.69 mmol). The reaction mixture was stirred at rt for 15 h, then diluted with H_2O (15 mL) and extracted with CH_2Cl_2 (3 × 15 mL). The combined organic fractions were washed with brine, dried ($MgSO_4$) and concentrated *in vacuo*. The crude residue was purified by FCC (20% EtOAc/PE) to yield **8** (40.0 mg, 0.176

mmol, 52%) as a clear oil. **R$_f$** 0.36 (SiO_2; 25% EtOAc/PE); **ν$_{max}$** (neat/cm^{-1}) 3298, 2936, 1635, 1539, 1458, 1420; **δ$_H$** (500 MHz, $CDCl_3$) 6.58 (dd, 2H, *J* = 17.4, 10.6 Hz), 6.53 (s, 1H), 6.38 (d, 2H, *J* = 17.3 Hz), 5.58 (dd, 2H, *J* = 10.6, 1.4 Hz), 5.29 (s, 1H), 3.51 (q, 2H, *J* = 6.6 Hz), 2.25 (td, 2H, *J* = 10.5, 2.7 Hz), 1.95 (t, 1H, *J* = 2.7 Hz), 1.78-1.72 (m, 2H), 1.67-1.61 (m, 2H); **δ$_C$** (126 MHz, $CDCl_3$) 163.7, 162.4, 135.8, 121.9, 105.7, 84.4, 68.7, 41.0, 28.9, 25.9, 18.3; **HRMS** (ESI) *m/z* found $[M+H]^+$ 228.1497, $C_{14}H_{18}N_3^+$ required 228.1495.

Ethyl *N*-(4,6-dichloropyrimidin-2-yl)-*N*-methylglycinate (26)

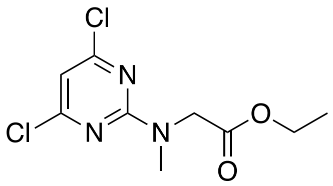

To a solution of 2,4,6-trichloropyrimidine (1.00 g, 5.45 mmol) in acetone (6 mL) at 0 °C was added sarcosine ethyl ester hydrochloride (1.01 g, 6.54 mmol) followed by the slow addition of triethylamine (1.90 mL, 13.6 mmol) and the reaction mixture stirred at 0 °C for 90 min. Upon completion, the solvent was removed *in vacuo* then redissolved in H_2O (20 mL) and extracted with CH_2Cl_2 (4 × 20 mL). The combined organic fractions were dried ($MgSO_4$), concentrated *in vacuo* and the crude residue purified by FCC (2-20% EtOAc/PE) to yield **26** (266 mg, 1.01 mmol, 19%) as a clear oil. **R$_f$** 0.34 (SiO_2; 10% EtOAc/PE); **m.p.** 40-42 °C; **ν$_{max}$** (neat/cm^{-1}) 1747, 1567, 1511, 1413, 1198; **δ$_H$** (400 MHz, $CDCl_3$) 6.59 (s, 1H), 4.33 (s, 2H), 4.21 (q, 2H, *J* = 7.2 Hz), 3.23 (s, 3H), 1.28 (t, 3H, *J* = 7.2 Hz); **δ$_C$** (101 MHz, $CDCl_3$) 169.5, 161.4, 108.8, 61.4, 51.4, 36.8, 14.4; **HRMS** (ESI) *m/z* found $[M+H]^+$ 264.0299, $C_9H_{12}Cl_2N_3O_2^+$ required 264.0307.

Ethyl *N*-(4,6-divinylpyrimidin-2-yl)-*N*-methylglycinate (27)

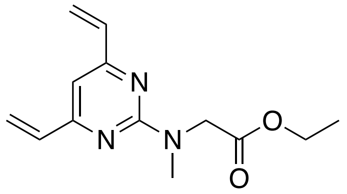

26 (204 mg, 0.722 mmol), potassium vinyltrifluoroborate (517 mg, 3.86 mmol), $Pd(dppf)Cl_2{\cdot}CH_2Cl_2$ (94.6 mg, 0.116 mmol) and potassium carbonate (641 mg, 4.63

mmol) in THF/H_2O (10:1, 5.5 mL) were heated to 70 °C for 17 h. Upon completion, the reaction mixture was filtered through Celite® and the solvent removed *in vacuo*. The resulting residue was purified by FCC (0-4% EtOAc/PE) to yield **27** (182 mg, 0.736 mmol, 95%) as an off-white solid. **R_f** 0.30 (SiO_2; 10% EtOAc/PE); **ν_{max}** (neat/cm^{-1}); 1747, 1560, 1540, 1508, 1401, 1196; **δ_H** (400 MHz, $CDCl_3$) 6.59 (dd, 2H, *J* = 17.2, 10.5 Hz), 6.49 (s, 1H), 6.38 (d, 2H, *J* = 17.2 Hz), 5.53 (dd, 2H, *J* = 10.5, 1.0 Hz), 4.36 (s, 2H), 4.18 (q, 2H, *J* = 7.1 Hz), 3.32 (s, 3H), 1.23 (t, 3H, *J* = 7.2 Hz); **δ_C** (101 MHz, $CDCl_3$) 171.1, 162.2, 136.2, 121.3, 105.7, 60.8, 51.8, 36.6, 14.4; **HRMS** (ESI) *m/z* found $[M+H]^+$ 248.1397, $C_{13}H_{18}N_3O_2^+$ required 248.1399.

_N_-(4,6-divinylpyrimidin-2-yl)-_N_-methylglycine (9)

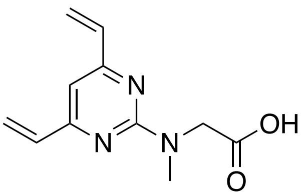

To a solution of **27** (40.0 mg, 0.162 mmol) in THF/H_2O (1:1, 2 mL) was added LiOH·H_2O (14.9 mg, 0.178 mmol) and the reaction mixture stirred at rt for 18 h. Upon completion, the mixture was diluted with H_2O (10 mL) and washed with Et_2O (10 mL). The aqueous phase was neutralized with 1M HCl and extracted with CH_2Cl_2 (4 × 20 mL). The combined organic fractions were dried ($MgSO_4$) and concentrated *in vacuo*. The crude residue was triturated with PE to yield **9** (29.0 mg, 0.132 mmol, 82%) as a pale yellow solid. **m.p.** 92-95 °C; **ν_{max}** (neat/cm^{-1}) 2935, 1705, 1630, 1536, 1395, 1243, 1220; **δ_H** (400 MHz, CD_3OD) 6.67-6.60 (m, 3H), 6.40 (dd, 2H, *J* = 17.4, 1.5 Hz), 5.55 (dd, 2H, *J* = 10.6, 1.5 Hz), 4.36 (s, 2H), 3.28 (s, 3H); **δ_C** (101 MHz, $CDCl_3$) 174.6, 164.8, 163.4, 137.2, 121.8, 106.1, 52.1, 36.7; **HRMS** (ESI) *m/z* found $[M+H]^+$ 220.1087, $C_{11}H_{14}N_3O_2^+$ required 220.1086.

Dox-PEG$_4$-N$_3$ (19)

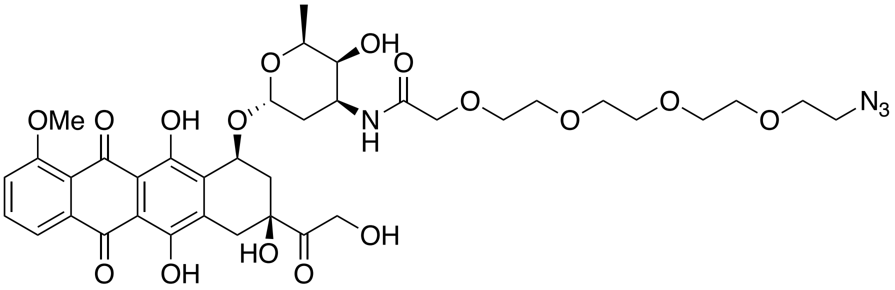

To a solution of doxorubicin hydrochloride (10.9 mg, 20.0 µmol) in DMF (1.10 mL) was added DIPEA (7.00 µL, 40.0 µmol) followed by HBTU (9.10 mg, 24.0 µmol). The mixture was stirred at rt for 5 min. 14-azido-3,6,9,12-tetraoxatetradecanoic acid (0.5 M in TBME , 44.5 µL, 20.0 mmol) was added and the mixture stirred for 2.5 h. Upon completion, the reaction mixture was diluted with H_2O (10 mL) and the product extracted in CH_2Cl_2 (4 × 10 mL), dried ($MgSO_4$). The organic fractions were concentrated and the crude residue purified by FCC (5% MeOH/EtOAc) to yield **19** (10.9 mg, 13.6 µmol, 65%) as a clear oil. **R$_f$** 0.22 (SiO_2; 10% MeOH/CH_2Cl_2); **ν_{max}** (neat/cm^{-1}) 2922, 2106, 1724, 1654, 1619, 1578, 1535, 1411; **δ_H** (500 MHz, $CDCl_3$) 8.04 (d, 1H, *J* = 7.8 Hz), 7.78 (t, 1H, *J* = 8.1 Hz), 7.39 (d, 1H, *J* = 8.0 Hz), 5.51 (d, 1H, *J* = 3.8 Hz), 5.30-5.29 (m, 1H), 4.77 (d, 2H, *J* = 3.1 Hz), 4.61 (s, 1H), 4.22-4.16 (m, 1H), 4.13 (q, 1H, *J* = 6.5 Hz), 4.08 (s, 3H), 3.93 (m, 2H), 3.72-3.63 (m, 15H), 3.40 (t, 2H, *J* = 5.0 Hz), 3.28 (dd, 1H, *J* = 18.8, 1.7 Hz), 3.04 (s, 1H), 2.37 (d, 1H, *J* = 14.7 Hz), 2.16 (dd, 1H, *J* = 14.7, 4.0 Hz), 1.93 (td, 1H, *J* = 19.8, 4.2 Hz), 1.80 (dd, 1H, *J* = 13.4, 5.0 Hz), 1.29 (d, 3H, *J* = 6.6 Hz); **δ_C** (126 MHz, $CDCl_3$) 214.1, 187.3, 186.9, 169.4, 161.2, 156.4, 155.9, 135.9, 135.7, 133.8, 133.8, 121.1, 120.0, 118.6, 111.7, 111.5, 101.1, 76.8, 71.1, 70.9, 70.7, 70.6, 70.6, 70.4, 70.2, 70.2, 69.8, 69.3, 67.6, 65.7, 56.8, 50.8, 45.0, 35.8, 34.1, 29.8, 17.1; **HRMS** (ESI) *m/z* found $[M+H]^+$ 803.2968, $C_{37}H_{47}N_4O_{16}^+$ required 803.2982.

N$_3$-PEG$_4$-MMAE (23)

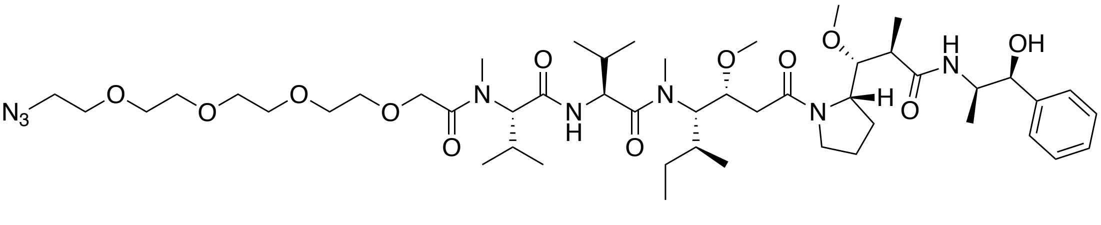

A solution of N_3-PEG_4-COOH (50.0 µL, 25.1 µmol, 0.5 M in TBME, 90%), HBTU (12.7 mg, 33.4 µmol) and DIPEA (17.5 µL, 100 µmol) in DMF (0.5 mL) was stirred at rt for 15 min. To this was added a solution of MMAE (12.0 mg, 16.7 µmol) in DMF (0.5 mL) and the reaction mixture stirred at rt for 18 h. Upon completion, the solvent was removed under a stream of N_2 and the crude residue was purified by FCC (0-5% MeOH/CH_2Cl_2) to yield **23** (14.6 mg, 14.9 µmol, 90%) as a clear oil. **R_f** 0.48 (SiO_2; 10% MeOH/CH_2Cl_2); **ν_{max}** (neat/cm^{-1}) 3423, 2926, 2109, 1629, 1454, 1098; **HPLC** (5-95% MeCN/H_2O over 20 min) retention time 11.097 min; **HRMS** (ESI) *m/z* found $[M+Na]^+$ 999.6100, $C_{49}H_{84}N_8O_{12}Na_1^+$ required 999.6100.

DVP-PEG_4-MMAE (24)

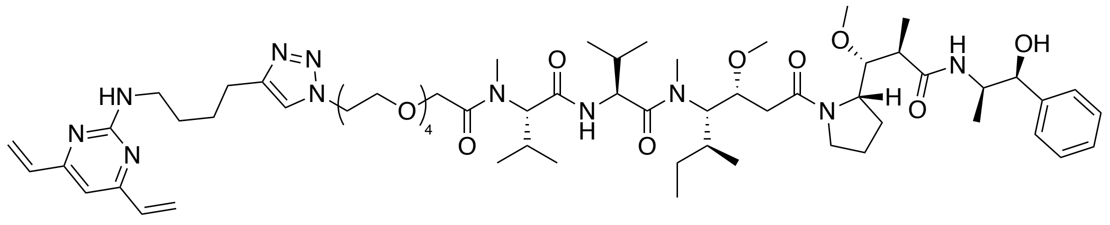

To a degassed solution of **23** (4.90 mg, 5.00 µmol) and **9** (2.30 mg, 10.0 µmol) in CH_2Cl_2 (0.25 mL) was added a degassed solution of $CuSO_4 \cdot 5H_2O$ (1.50 mg, 6.00 µmol), THPTA (4.40 mg, 10.0 µmol) and sodium ascorbate (5.00 mg, 25.0 µmol) in H_2O/^{t}BuOH (0.5 mL, 1:1) and the reaction mixture stirred at rt for 2 h. Upon completion, the reaction was diluted with H_2O (15 mL) and extracted with CH_2Cl_2 (5 × 15 mL). The combined organic fractions were dried ($MgSO_4$), concentrated *in vacuo* and the crude residue purified by FCC (0-10% MeOH/CH_2Cl_2) to yield **24** (1.80 mg, 1.50 µmol, 30%) as a clear oil. **R_f** 0.28 (SiO_2; 7.5% MeOH/CH_2Cl_2); **ν_{max}** (neat/cm^{-1}) 3301, 2926, 1632, 1544, 1452, 1098; **HPLC** (5-95% MeCN/H_2O over 20 min) retention time 10.309 min; **HRMS** (ESI) *m/z* found $[M+Na]^+$ 1226.7502, $C_{63}H_{101}N_{11}O_{12}Na_1^+$ required 1226.7523.

Dimethyl 3,3'-(((2-aminopyrimidine-4,6-diyl)bis(ethane-2,1-diyl))bis(sulfanediyl))(2*R*,2'*R*)-bis(2-((*tert*-butoxycarbonyl)amino)propanoate) (28)

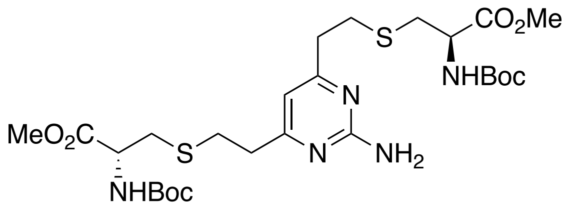

A solution of **7** (10.0 mg, 68.0 µmol), *N*-(*tert*-butoxycarbonyl)-*L*-cysteine methyl ester (40.0 mg, 170 µmol) in 30% MeCN/sodium phosphate buffer (pH 8, 50 mM)(1.36 mL) was stirred at 37 °C for 2 h. Upon completion, the mixture was diluted with H_2O (10 mL), extracted with EtOAc (4 × 10 mL), dried ($MgSO_4$) and concentrated *in vacuo*. The crude residue was purified by FCC (70% EtOAc/PE) to yield **28** (23.0 mg, 37.0 µmol, 55%) as a clear oil. $\mathbf{R_f}$ 0.33 (SiO_2; 70% EtOAc/PE); $\mathbf{\nu_{max}}$ (neat/cm^{-1}) 3359, 2977, 1742,1703, 1615, 1579, 1560, 1503, 1437; $\mathbf{\delta_H}$ (400 MHz, CD_3OD) 6.54 (s, 1H), 4.37-4.34 (m, 2H), 3.73 (s, 6H), 2.99-2.79 (m, 12H) 1.44 (s, 18H); $\mathbf{\delta_C}$ (101 MHz, CD_3OD) 173.3, 171.1, 164.7, 157.8, 110.5, 80.8, 55.1, 52.9, 38.5, 34.6, 31.6, 28.7; **HRMS** (ESI) *m/z* found $[M+H]^+$ 640.2469, $C_{26}H_{43}N_5O_8{}^{32}S_2Na_1{}^+$ required 640.2451.

Dimethyl 3,3'-(((2-(hex-5-yn-1-ylamino)pyrimidine-4,6-diyl)bis(ethane-2,1-diyl))bis(sulfanediyl))(2*R*,2'*R*)-bis(2-((*tert*-butoxycarbonyl)amino)propanoate) (29)

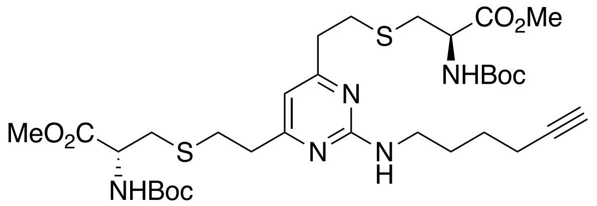

A solution of **8** (10.0 mg, 44.0 µmol), *N*-(*tert*-butoxycarbonyl)-*L*-cysteine methyl ester (25.9 mg, 110 µmol) in 30% MeCN/sodium phosphate buffer (pH 8, 50 mM)(880 µL) was stirred at 37 °C for 1 h. Upon completion, the mixture was diluted with H_2O (10 mL), extracted with CH_2Cl_2 (4 × 10 mL), dried ($MgSO_4$) and concentrated *in vacuo*. The crude residue was purified by FCC (100% EtOAc/PE) to yield **29** (28.0 mg, 40.0 µmol, 91%) as a clear oil. $\mathbf{R_f}$ 0.07 (SiO_2; 70% EtOAc/PE); $\mathbf{\nu_{max}}$ (neat/cm^{-1}) 2976, 2344, 1744, 1711, 1642, 1565; $\mathbf{\delta_H}$ (500 MHz, $CDCl_3$) 6.25 (s, 1H), 5.54-5.41 (m, 2H), 5.14 (s, 1H), 4.61-4.54 (m, 2H), 3.74 (s, 6H), 3.45-3.41 (m, 2H), 2.98 (d, 4H, *J* = 5.1 Hz), 2.91-2.86 (m, 4H), 2.78-2.75 (m, 4H), 2.23 (td, 2H, *J* = 10.5, 2.6 Hz), 1.95 (t, 1H, *J* = 2.5 Hz), 1.73-1.72 (m, 2H), 1.65-1.59 (m, 2H), 1.44 (s, 18H); $\mathbf{\delta_C}$ (126 MHz, $CDCl_3$) 171.7, 168.8, 162.7, 155.3, 108.9, 84.4, 80.3, 68.7, 60.5, 53.5, 52.7, 40.9, 37.7, 34.8, 31.0, 28.5,

25.9, 18.3; **HRMS** (ESI) *m/z* found $[M+H]^+$ 698.3243, $C_{32}H_{52}N_5O_8{}^{32}S_2{}^+$ required 698.3252.

***N*-(4,6-bis(2-(((*R*)-2-((*tert*-butoxycarbonyl)amino)-3-methoxy-3-oxopropyl)thio)ethyl)pyrimidin-2-yl)-*N*-methylglycine (30)**

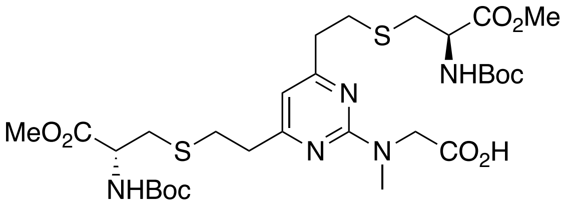

A solution of **9** (5.00 mg, 22.8 µmol), *N*-(*tert*-butoxycarbonyl)-*L*-cysteine methyl ester (13.4 mg, 57.0 µmol) in 30% MeCN/sodium phosphate buffer (pH 8, 50 mM)(456 µL) was stirred at 37 °C for 1 h. Upon completion, the mixture was diluted with H_2O (10 mL), extracted with CH_2Cl_2 (4 × 10 mL), dried ($MgSO_4$) and concentrated *in vacuo*. The crude residue was purified by FCC (70-100% EtOAc/PE then 1% AcOH/EtOAc) to yield **30** (8.6 mg, 12.5 µmol, 55%) as a clear oil. $\mathbf{R_f}$ 0.17 (SiO_2; 70% EtOAc/PE); $\boldsymbol{\nu_{max}}$ (neat/cm^{-1}) 2974, 1704, 1561, 1509; $\boldsymbol{\delta_H}$ (500 MHz, CD_3OD) 6.46 (s, 1H), 4.34-4.32 (m, 4H), 3.72 (s, 6H), 3.24 (s, 3H), 2.97-2.91 (m, 6H), 2.85-2.81 (m, 6H), 1.44 (s, 18H); $\boldsymbol{\delta_C}$ (126 MHz, CD_3OD) 174.7, 173.3, 170.1, 163.5, 157.8, 109.6, 80.8, 55.2, 52.9, 52.2, 38.7, 36.7, 34.7, 31.6, 28.7; **HRMS** (ESI) *m/z* found $[M+H]^+$ 690.2831, $C_{29}H_{48}N_5O_{10}{}^{32}S_2{}^+$ required 690.2837.

Cysteine/Lysine Selectivity

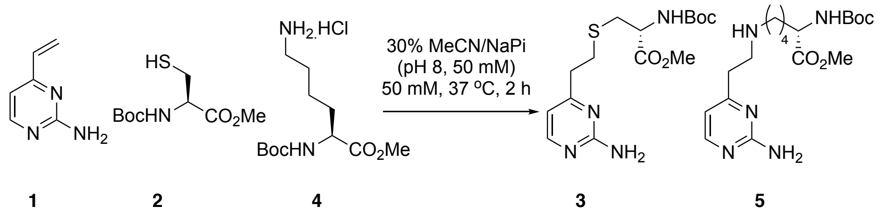

A solution of 2-amino-4-vinylpyrimidine, **1** (12.0 mg, 99.0 µmol), *N*-(*tert*-butoxycarbonyl)-*L*-cysteine methyl ester (7.80 mg, 33.0 µmol) and *N*α-(*tert*-butoxycarbonyl)-*L*-lysine methyl ester hydrochloride (9.70 mg, 33.0 µmol) in 30% MeCN/sodium phosphate buffer (pH 8, 50 mM, 1.98 mL) was stirred at 37 °C for 2 h.

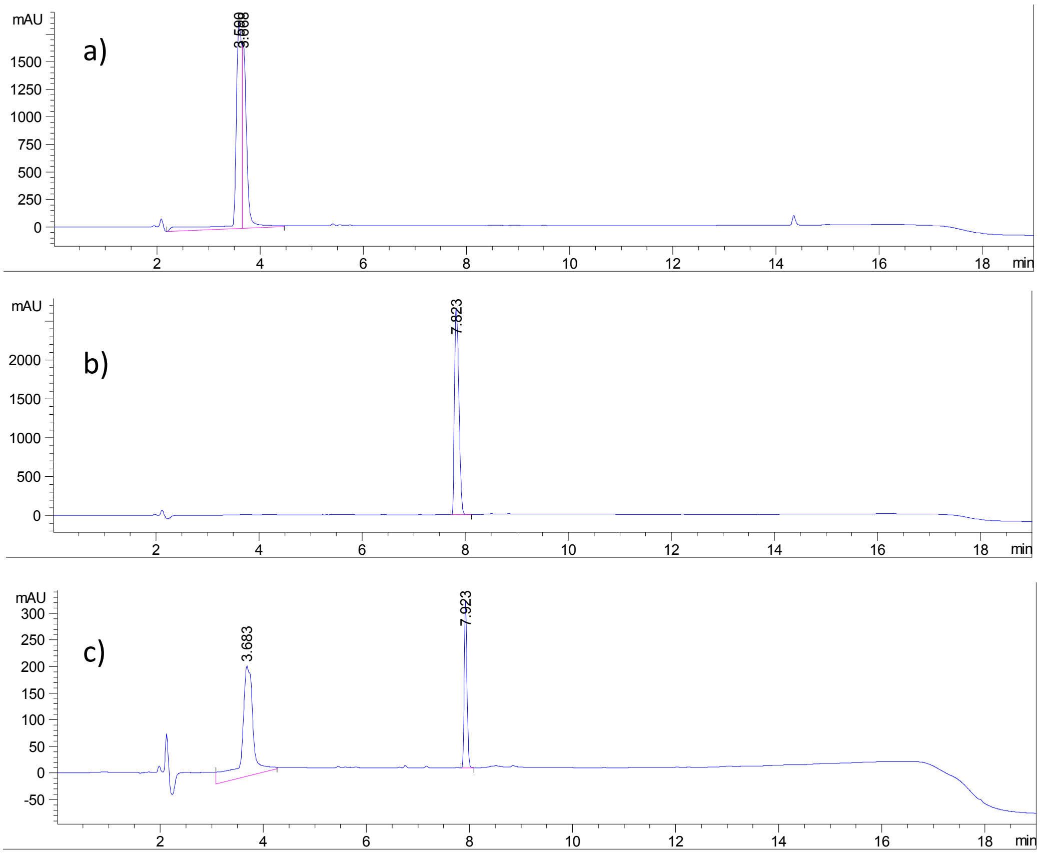

Fig. S1: a) 1, **b) 3**, and **c)** reaction of **1** with *N*-Boc-Cys-OMe and *N*-Boc-Lys-OMe.

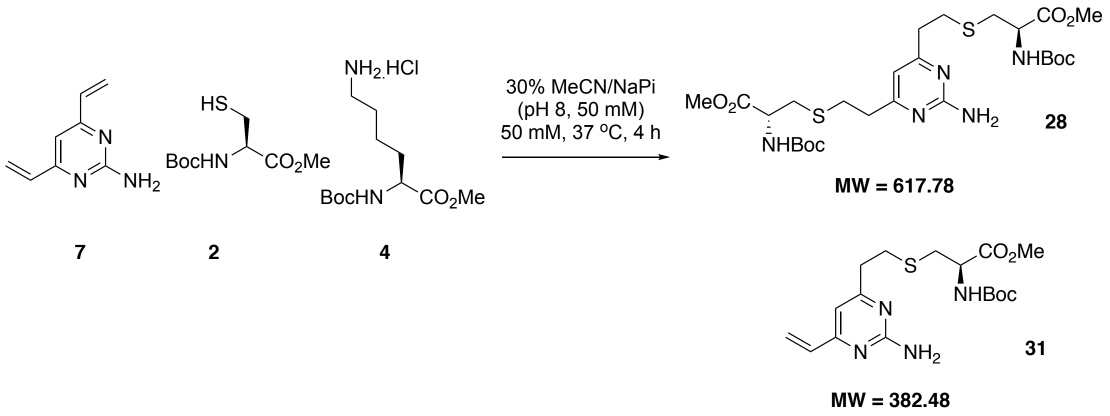

A solution of **7** (4.00 mg, 27.0 µmol), *N*-(*tert*-butoxycarbonyl)-*L*-cysteine methyl ester (2.10 mg, 9.10 µmol) and *N*α-(*tert*-butoxycarbonyl)-*L*-lysine methyl ester hydrochloride (2.70 mg, 9.10 µmol) in 30% MeCN/sodium phosphate buffer (pH 8, 50 mM, 540 µL) was stirred at 37 °C for 4 h.

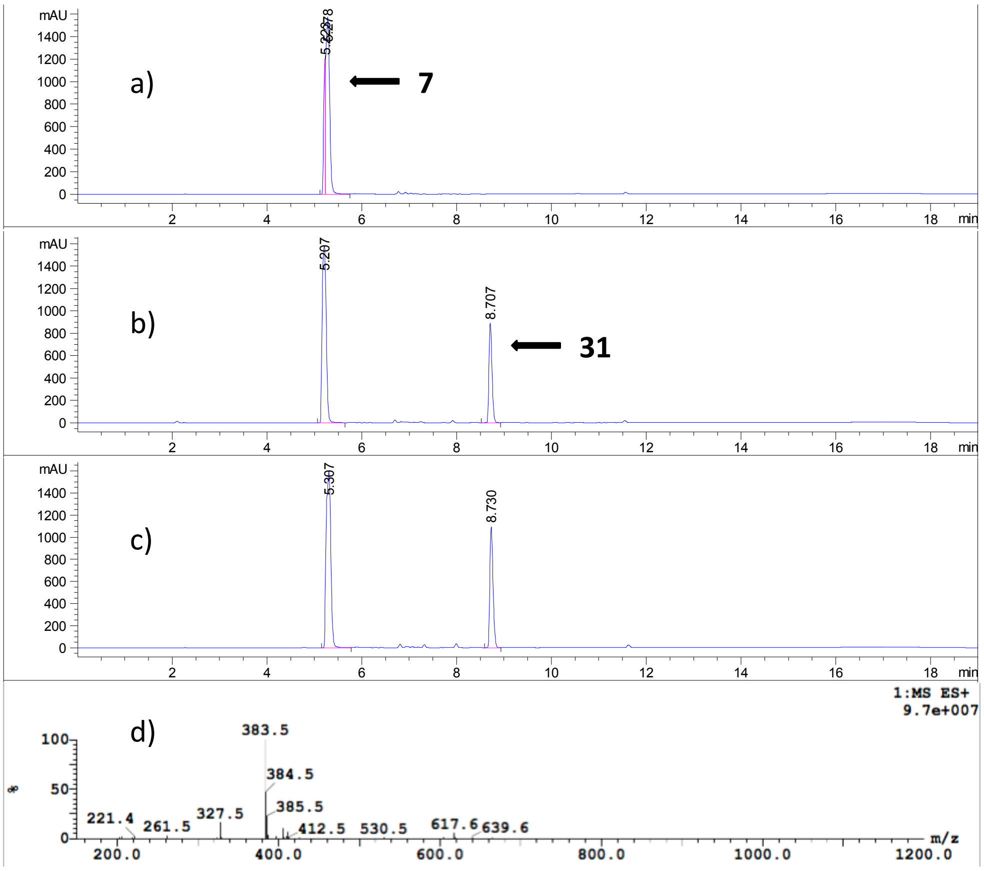

Fig. S2: a) **7**, **b)** reaction of **7** with *N*-Boc-Cys-OMe and *N*-Boc-Lys-OMe after 2 h, **c)** after 4 h and **d)** MS trace of reaction after 4 h.

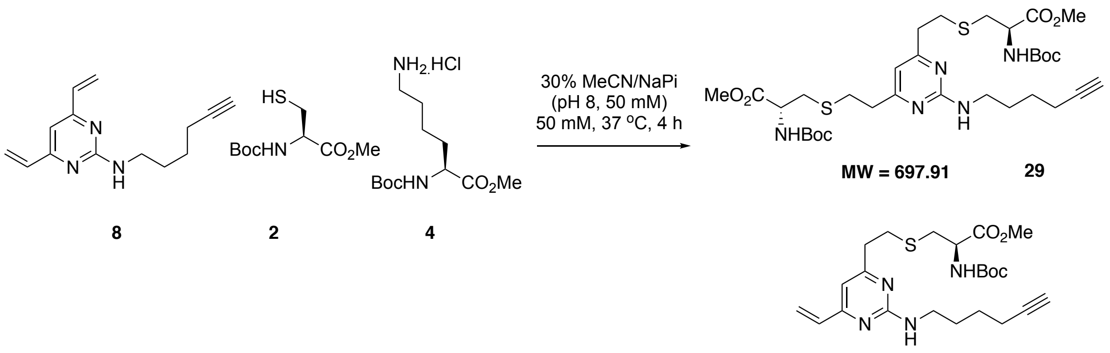

A solution of **8** (2.70 mg, 11.9 µmol), *N*-(*tert*-butoxycarbonyl)-*L*-cysteine methyl ester (1.00 mg, 4.00 µmol) and *N*α-(*tert*-butoxycarbonyl)-*L*-lysine methyl ester hydrochloride (1.20 mg, 4.00 µmol) in 30% MeCN/sodium phosphate buffer (pH 8, 50 mM, 238 µL) was stirred at 37 °C for 4 h.

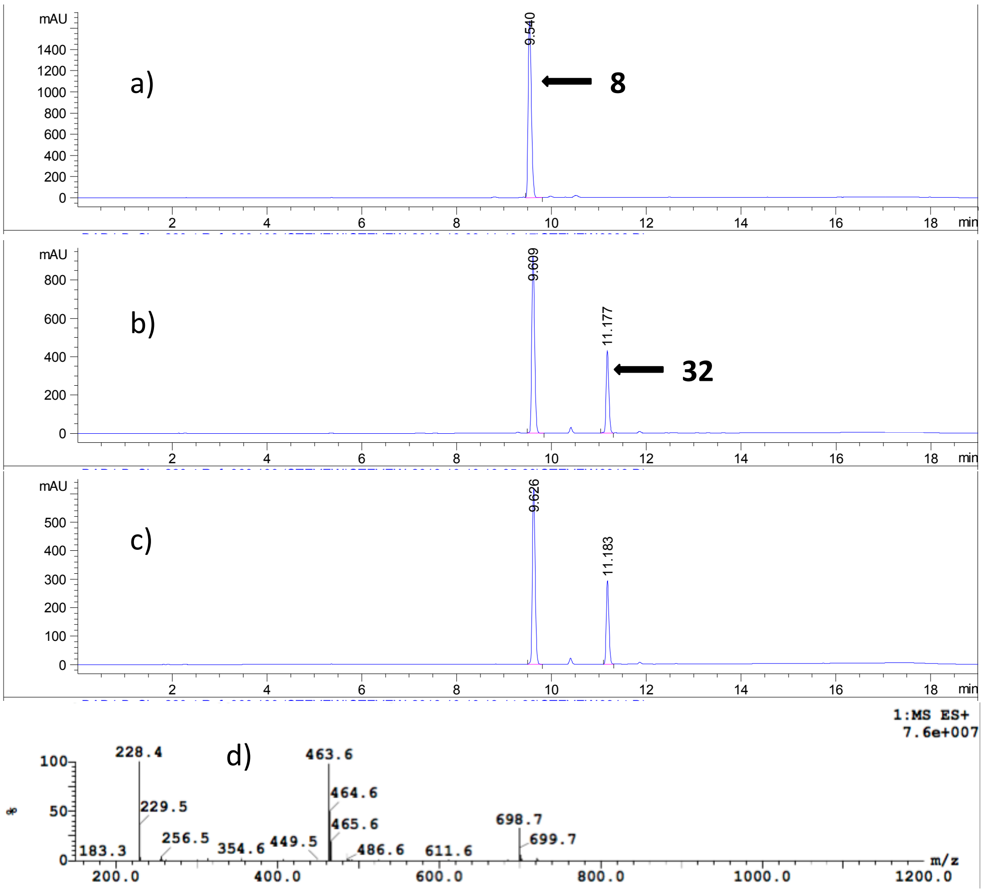

Fig. S3: a) 8, **b)** reaction of **8** with *N*-Boc-Cys-OMe and *N*-Boc-Lys-OMe after 2 h, **c)** after 4 h and **d)** MS trace of reaction after 4 h.

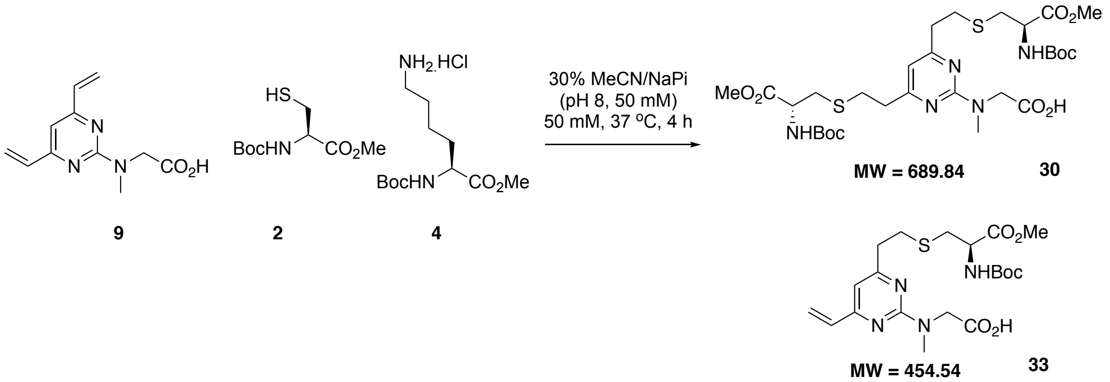

A solution of **9** (4.00 mg, 11.9 µmol), *N*-(*tert*-butoxycarbonyl)-*L*-cysteine methyl ester (1.50 mg, 4.00 µmol) and *N*α-(*tert*-butoxycarbonyl)-*L*-lysine methyl ester hydrochloride (1.80 mg, 4.00 µmol) in 30% MeCN/sodium phosphate buffer (pH 8, 50 mM, 364 µL) was stirred at 37 °C for 4 h.

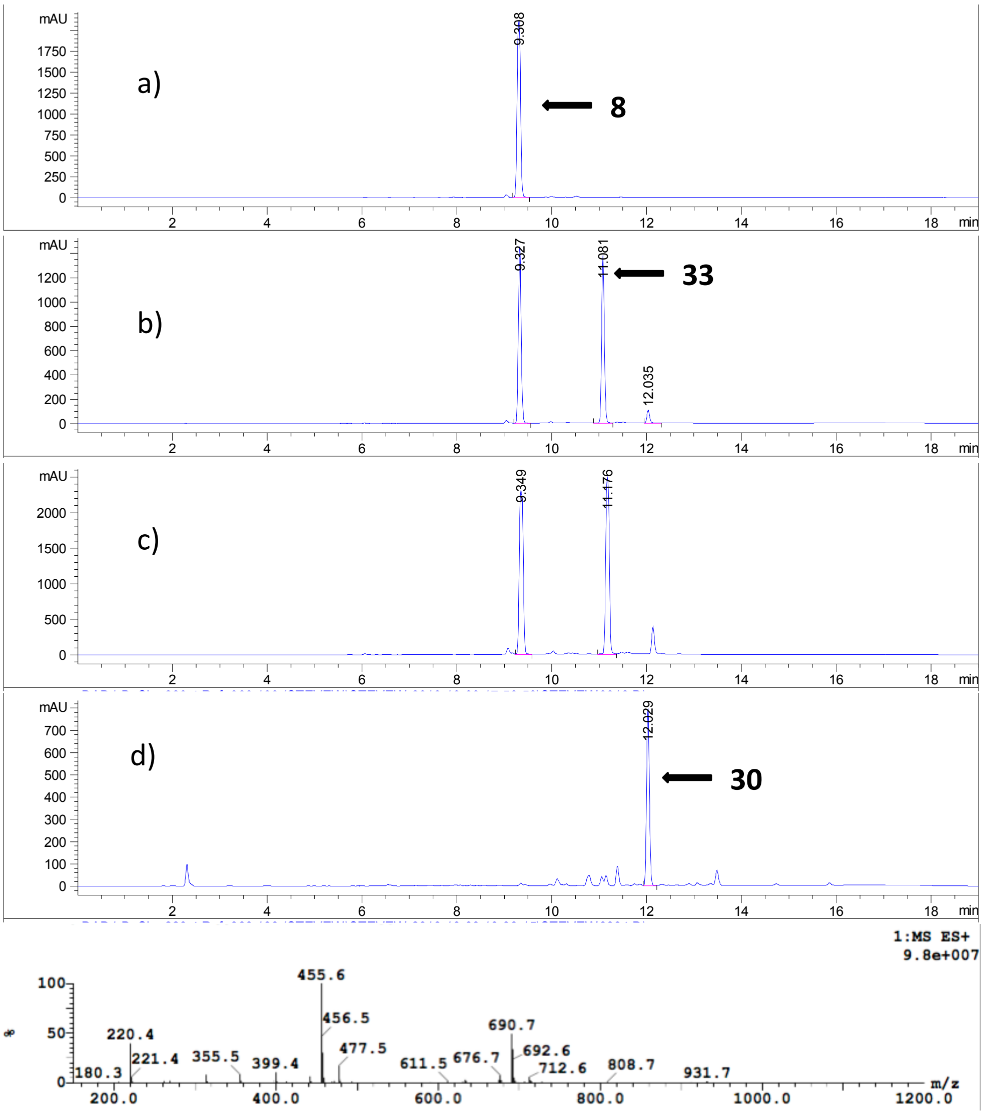

Fig. S4: a) **9**, **b)** reaction of **9** with *N*-Boc-Cys-OMe and *N*-Boc-Lys-OMe after 2 h, **c)** after 4 h, **d)** **30** and **e)** MS trace of reaction after 4 h.

TCEP Reactivity

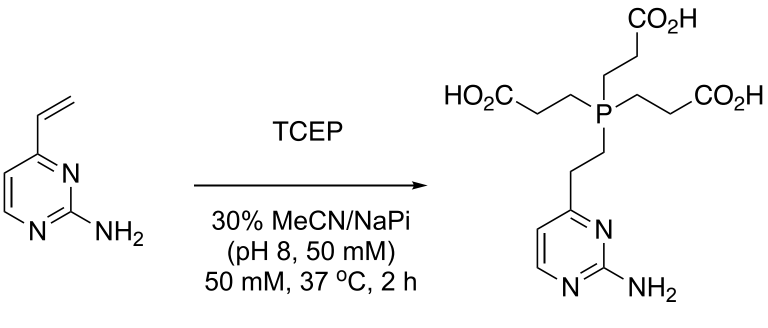

A solution of 2-amino-4-vinylpyrimidine, **1** (8.60 mg, 71.0 µmol) and tris(2-carboxyethyl)phosphine hydrochloride (TCEP) in 30% MeCN/sodium phosphate buffer (pH 8, 50 mM, 1.98 mL) was stirred at 37 °C for 2 h.

a)

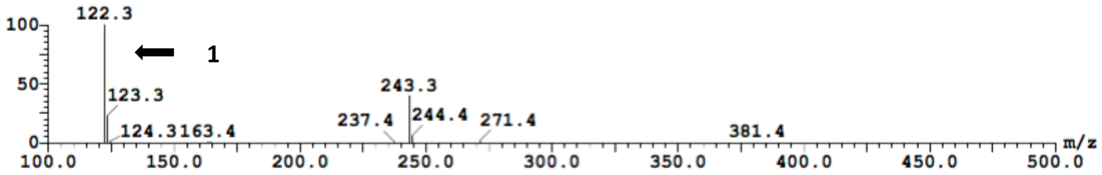

b)

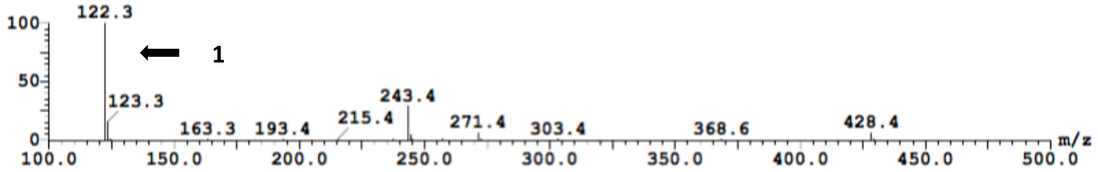

Fig. S5: Analysis of reactivity of vinylpyrimidine **1** with TCEP, **a)** at T=0 and **b)** after 2 hours. No evidence of the TCEP conjugate **28** was observed, expected $[M+H]^+$ = 373.

Stability Analysis

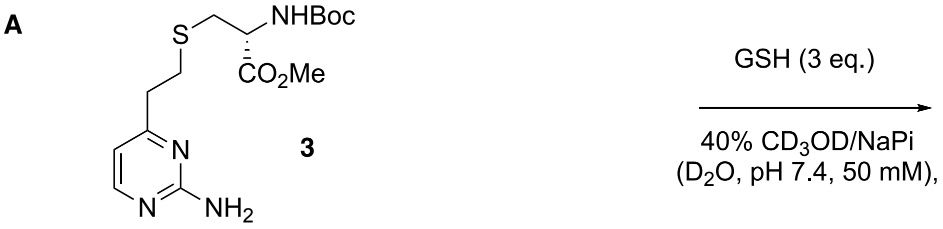

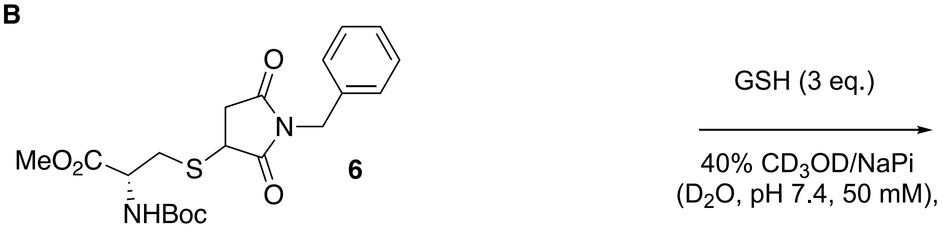

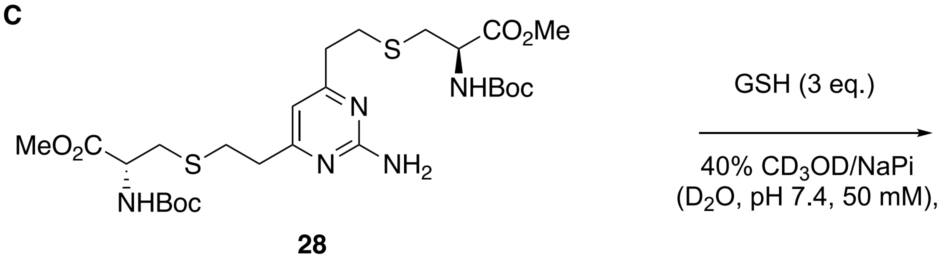

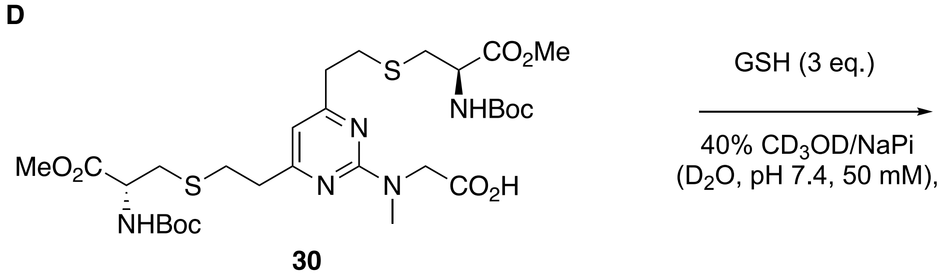

a)

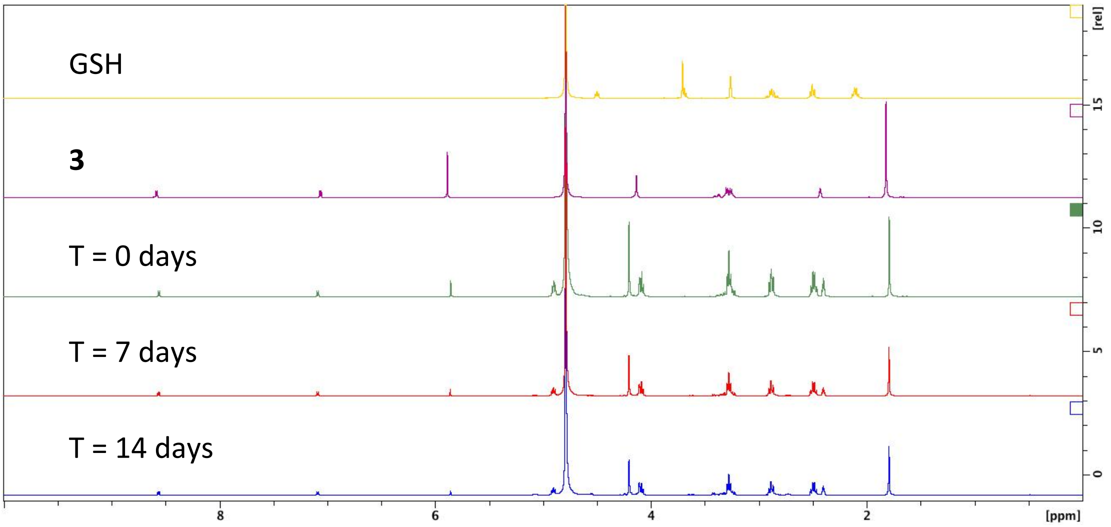

b)

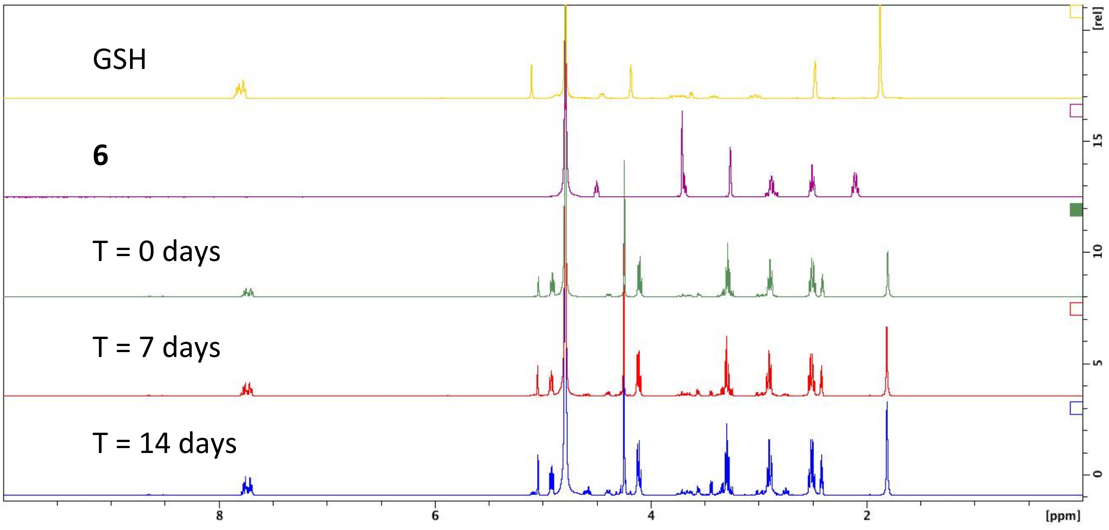
GSH
6
T = 0 days
T = 7 days
T = 14 days
[rel]
[ppm]

c)

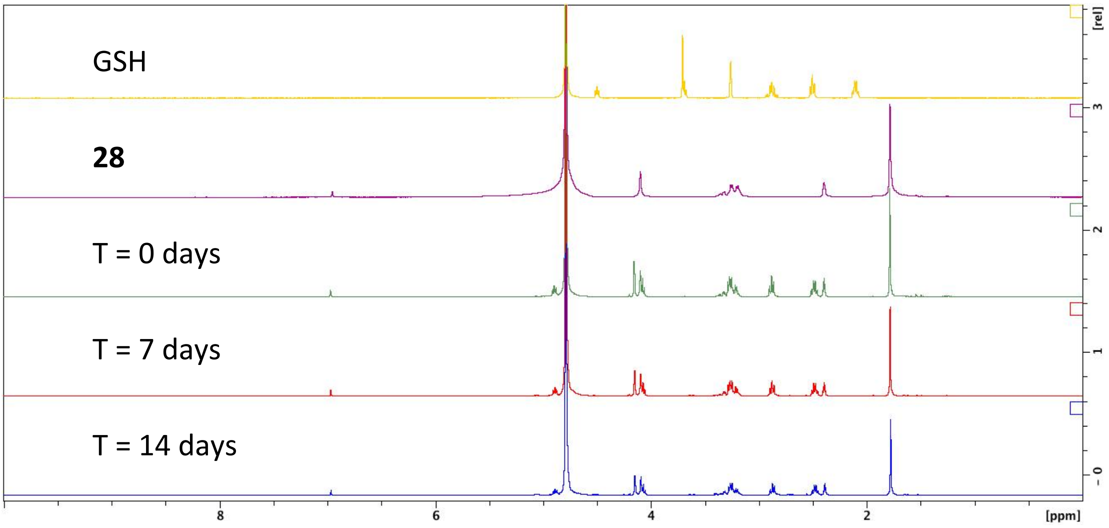
GSH
28
T = 0 days
T = 7 days
T = 14 days
[rel]
[ppm]

d)

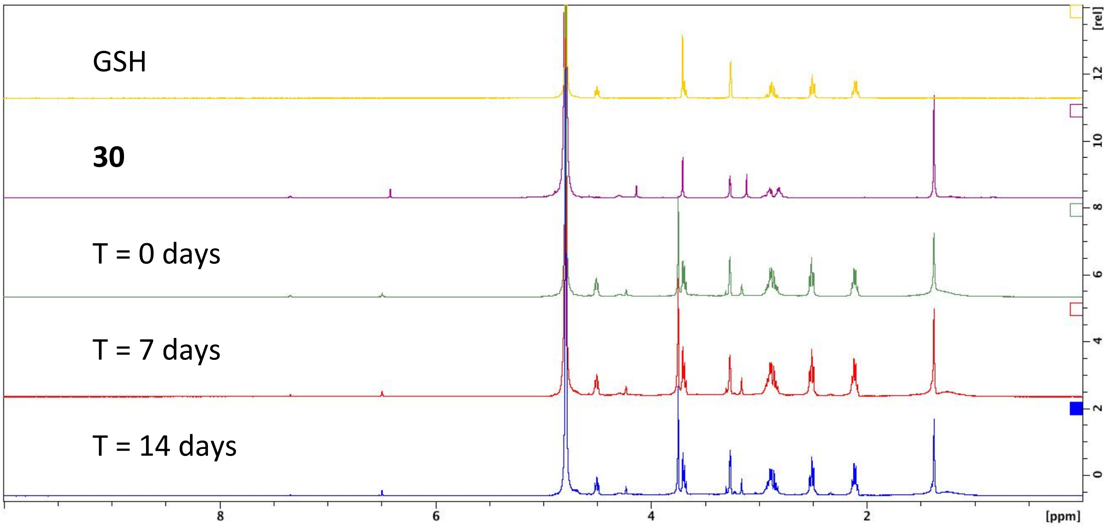
GSH
30
T = 0 days
T = 7 days
T = 14 days
[rel]
[ppm]

e)

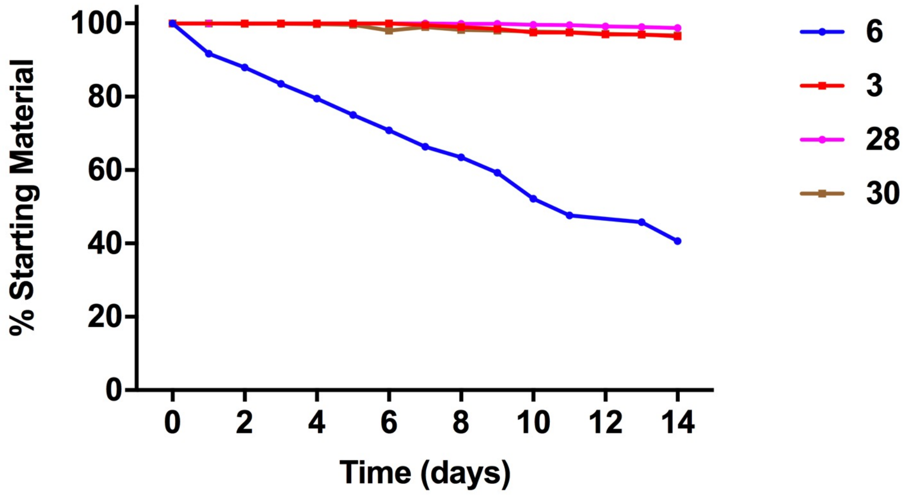

Fig. S6: a) stability analysis of **3** in reduced glutathione via ^{1}H NMR, **b)** stability analysis of **6** in reduced glutathione via ^{1}H NMR, **c)** stability analysis of **28** in reduced glutathione via ^{1}H NMR, **d)** stability analysis of **30** in reduced glutathione via ^{1}H NMR and **e)** graph of the stability of **3**, **6**, **28** and **30** in the presence of glutathione (GSH).

*Pf*RadA-dCys Preparation

Synthetic DNA Production

The synthetic gene of the *Pf*RadA C-terminal ATPase domain containing the desired cysteine mutations was constructed from DNA oligos using overlap-extension PCR. Oligos of approximately 25 bases (Sigma) were designed to include the cysteine mutations. Primers were present in the polymerase chain reaction (PCR) at a concentration of 1 µg/µL. PCR was conducted with Phusion polymerase (Thermo Scientific), and products were purified from agarose gel using a Gel Extraction kit (Qiagen). PCR products and pBAT4 vector were digested using *NcoI* and *XhoI* (New England Biolabs) for at least two hours at 37 °C, dephosphorylated with Shrimp alkaline phosphatase (SAP) (Agilent Technologies) and gel purified. The gene insert was ligated into plasmid using Quick Ligase Kit (New England Biolabs). The construct was then transformed into DH5α strain *E. coli* by heat shock at 42 °C. Transformants were selected using 100 µg/mL ampicillin in Luria-Bertani (LB) medium. Plasmid was extracted from 2 mL overnight cell cultures using Miniprep Spin kit (Qiagen). The insert was verified by restriction digest analysis and DNA sequencing.

Expression and Purification

The plasmids containing the desired insert were transformed into the BL21(DE3)-pUBS520 strain of *E. coli* and soluble expression of mutated proteins was confirmed in a small scale expression test. For large scale expression, cells were streaked from previously stored glycerol stocks on ampicillin- and kanamycin-supplemented LB agar plates. Colonies were grown overnight at 37 °C and used to inoculate 1 L cultures of LB media with ampicillin (100 µg/mL) and kanamycin (25 µg/mL). After 4.5 hours incubation at 37 °C (OD_{600} = 0.7 – 1.0), cells were induced with IPTG (400 mM). Further incubation at 37 °C was carried out for 3 hours, followed by centrifugation of the cultures. The cell pellet was resuspended in 20 mM MES pH 6.0. Cells were lysed using EmulsiFlex C5 homogeniser (Avestin). The cell lysate was heated to 65 °C for 10 minutes followed by centrifugation. The clarified supernatant was loaded onto a 5 mL HiTrap SP-Sepharose HP column using an ÄKTA purifier 10 chromatography system. The protein was eluted using ion exchange elution buffer (20 mM MES pH 6.0, 0.5 mM EDTA, 1 M NaCl) gradient of 0 – 0.5 M NaCl. Appropriate fractions were concentrated to 2 mL using Amico Ultra-4 centrifugal filter units, 10 kDa MWCO (Millipore). One tenth volume of concentrated sample (~180 µL) TCEP was added to each sample and subsequently loaded onto Superdex 75 16/60 gel filtration (GF) column equilibrated with 20 mM MES pH 6.0, 1 mM EDTA, 100 mM NaCl, 0.1 mM TCEP using an ÄKTA purifier 10 chromatography system. The protein was eluted using GF buffer (20 mM MES pH 6.0, 1 mM EDTA, 100 mM NaCl, 0.1 mM TCEP). Fractions containing purified *Pf*RadA-dCys were concentrated to 2 mL using Amico Ultra-4 centrifugal filter units, 10 kDa MWCO and protein purity analyzed by 15% SDS-PAGE (Fig. S21).

*Pf*RadA-dCys 7 Conjugation

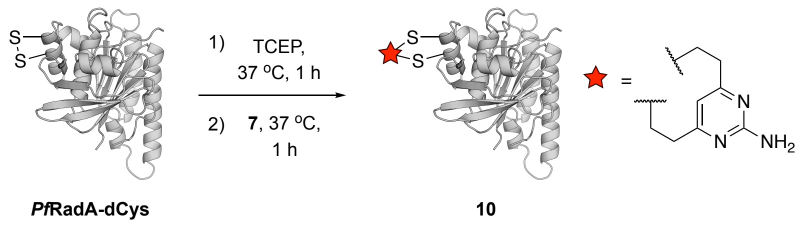

To a solution of *Pf*RadA-dCys (10 µL, 2.94 mg/mL) in Tris (25 mM Tris HCl pH 8, 25 mM NaCl, 0.5 mM EDTA, 3 M guanidine hydrochloride) was added TCEP (5 eq.). The mixture was vortexed and incubated at 37 °C for 1 h. A solution of **7** (10 mM in DMSO) was added (15 eq.) and the reaction mixture incubated at 37 °C for 1 h. The excess reagents were removed by repeated diafiltration into PBS using an Amicon-Ultra centrifugal filter (10000 MWCO, Merck Millipore). LC-MS analysis demonstrated >95% conversion to the desired conjugate.

a)

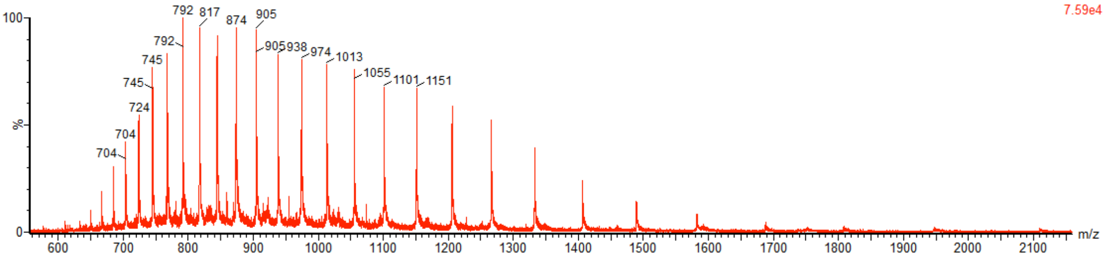

b)

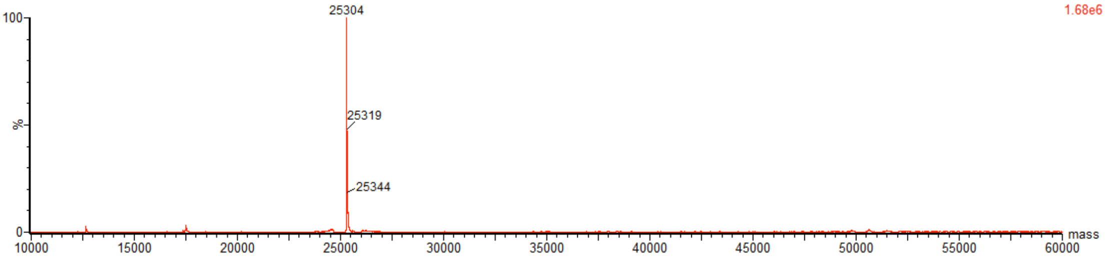

c)

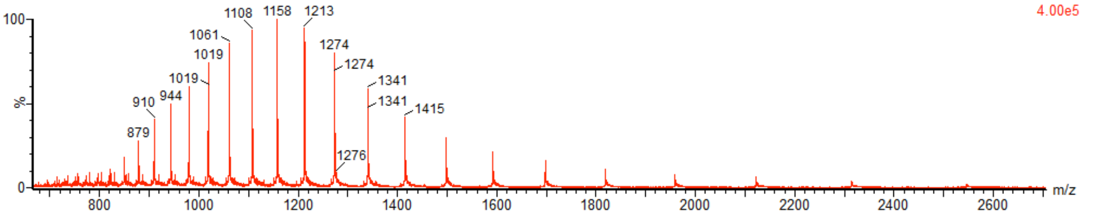

d)

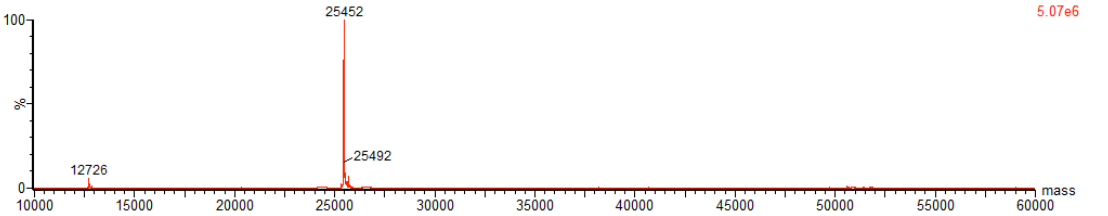

Fig. S7: LC-MS of reaction between *Pf*RadA-dCys and **7**, a) non-deconvoluted MS of unmodified *Pf*RadA-dCys, b) deconvoluted MS of unmodified *Pf*RadA-dCys; expected 25,304 Da, observed 25,304 Da, c) non-deconvoluted MS after reaction with **7** and d) deconvoluted MS after reaction with **7**; expected 25,451 Da, observed 25,452 Da.

*Pf*RadA-dCys 8 Conjugation

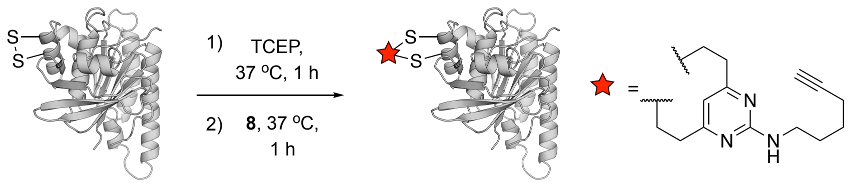

***Pf*RadA-dCys** **12**

To a solution of *Pf*RadA-dCys (10 µL, 2.94 mg/mL) in Tris (25 mM Tris HCl pH 8, 25 mM NaCl, 0.5 mM EDTA, 3 M guanidine hydrochloride) was added TCEP (5 eq.). The mixture was vortexed and incubated at 37 °C for 1 h. A solution of **8** (10 mM in DMSO) was added (15 eq.) and the reaction mixture incubated at 37 °C for 1 h. The excess reagents were removed by repeated diafiltration into fresh PBS using an Amicon-Ultra centrifugal filter (10000 MWCO, Merck Millipore). LC-MS analysis demonstrated >95% conversion to the desired conjugate.

a)

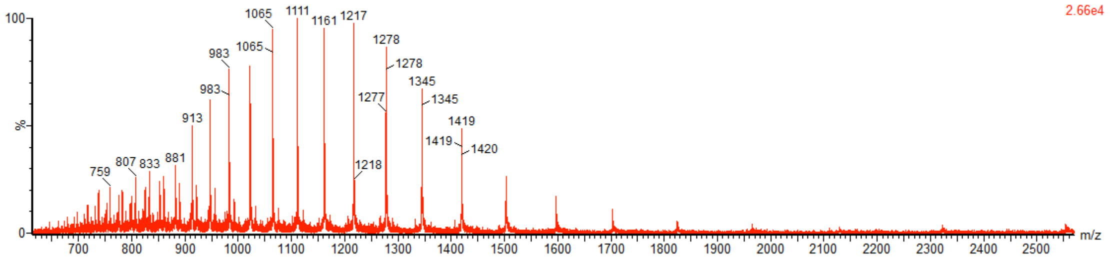

b)

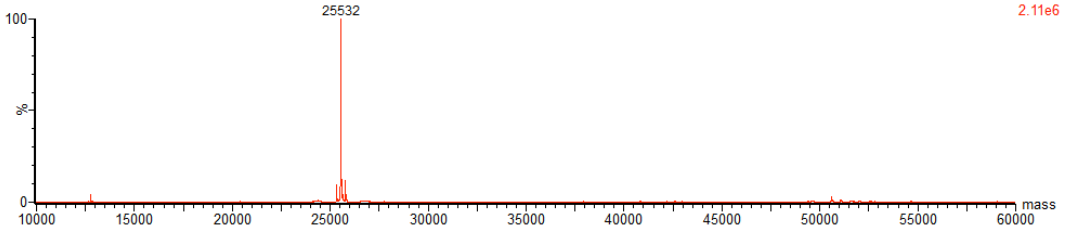

Fig. S8: LC-MS of reaction between *Pf*RadA-dCys and **8**, a) non-deconvoluted MS and b) deconvoluted MS; expected 25,531 Da, observed 25,532 Da.

*Pf*RadA-dCys 9 Conjugation

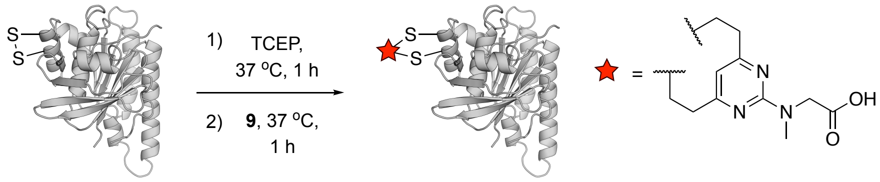

***Pf*RadA-dCys** **11**

To a solution of *Pf*RadA-dCys (10 µL, 2.94 mg/mL) in Tris (25 mM Tris HCl pH 8, 25 mM NaCl, 0.5 mM EDTA, 3 M guanidine hydrochloride) was added TCEP (5 eq.). The

mixture was vortexed and incubated at 37 °C for 1 h. A solution of **9** (10 mM in DMSO) was added (15 eq.) and the reaction mixture incubated at 37 °C for 1 h. The excess reagents were removed by repeated diafiltration into fresh PBS using an Amicon-Ultra centrifugal filter (10000 MWCO, Merck Millipore). LC-MS analysis demonstrated >95% conversion to the desired conjugate.

a)

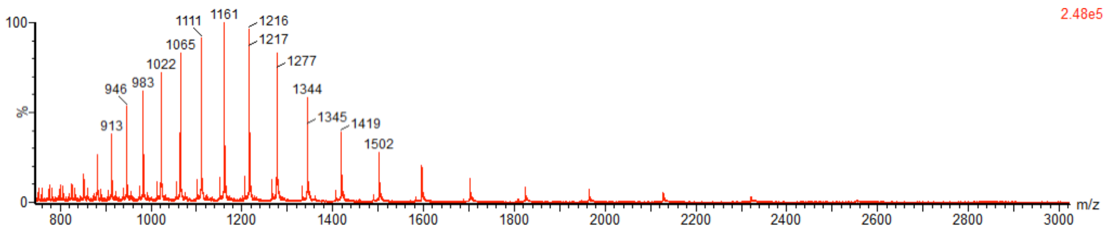

b)

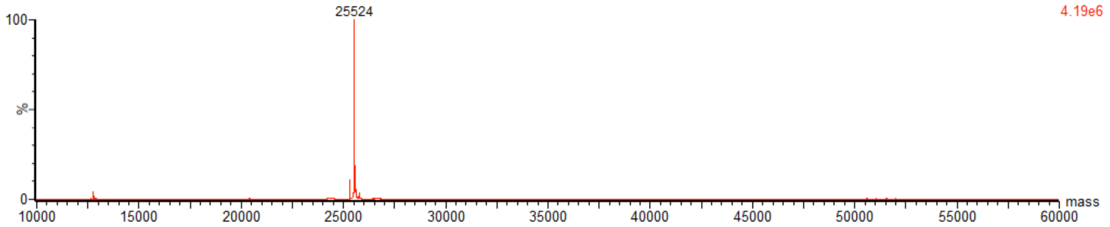

Fig. S9: LC-MS of reaction between *Pf*RadA-dCys and **9**, a) non-deconvoluted MS and b) deconvoluted MS; expected 25,523 Da, observed 25,524 Da.

Antibody Conjugation

Fab Preparation

Trastuzumab Fab was prepared using the Pierce™ Fab Preparation Kit (ThermoFisher). Briefly, 0.25 mL of immobilized papain was washed with Digestion Buffer containing cysteine·HCl to activate the papain. Trastuzumab (500 µL, 2.95 mg/mL) in Digestion Buffer was added to the immobilized papain and incubated with gentle mixing at 37 °C for 16 h. The digest was isolated from the papain and the Fab purified using a Nab Protein A Plus Spin Column followed by preparative size-exclusion chromatography (25 mM sodium borate pH 8, 100 mM NaCl, 0.5 mM EDTA). The isolated Fab was aliquoted and stored at -20 °C until use. Two different batches of trastuzumab Fab were prepared and used throughout this work.

a)

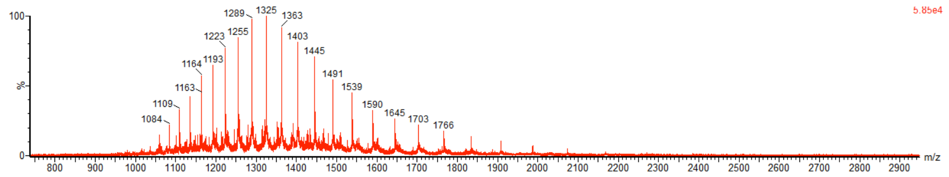

b)

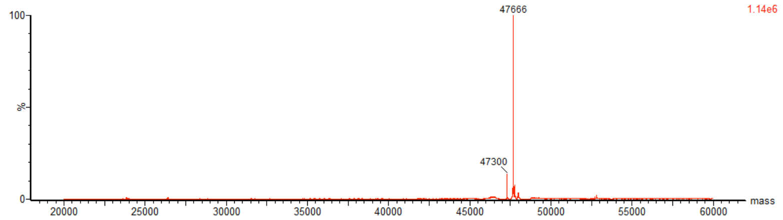

c)

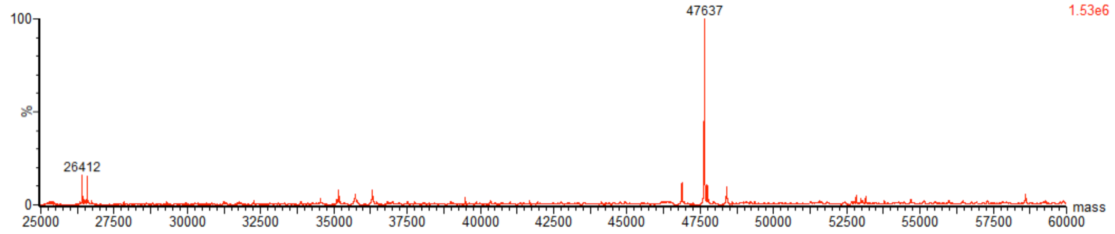

Fig. S10: LC-MS of trastuzumab Fab a) non-deconvoluted MS, b) deconvoluted MS and c) deconvoluted MS of second batch of Fab.

Trastuzumab Fab 7 conjugation

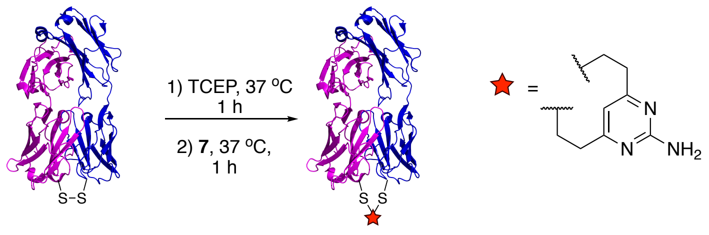

13

To a solution of trastuzumab Fab (10 µL, 27 µM, 1.28 mg/mL) in BBS (25 mM sodium borate pH 8, 25 mM NaCl, 0.5 mM EDTA) was added TCEP (5 eq.). The mixture was vortexed and incubated at 37 °C for 1 h. A solution of **7** (10 mM in DMSO) was added (final concentration of 270 µM, 10 eq.) and the reaction mixture incubated at 37 °C for 1 h. The excess reagents were removed by repeated diafiltration into fresh BBS using an Amicon-Ultra centrifugal filter (10000 MWCO, Merck Millipore). LC-MS analysis demonstrated >95% conversion to the desired conjugate.

a)

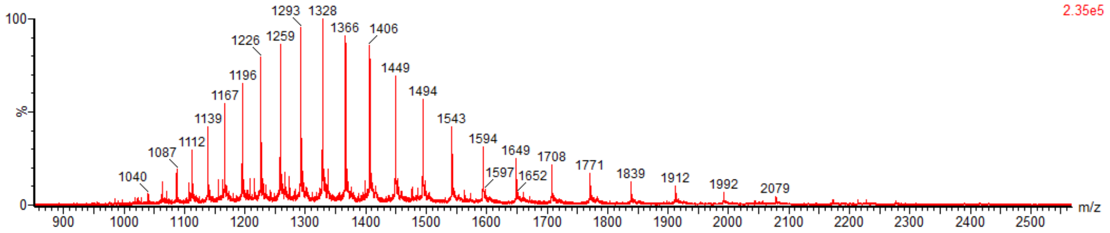

b)

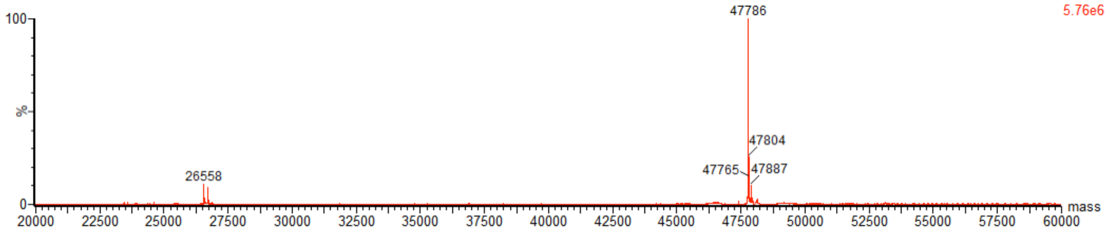

Fig. S11: LC-MS of reaction between trastuzumab Fab and **7**, a) non-deconvoluted MS and b) deconvoluted MS; expected 47,784 Da, observed 47,786 Da.

Trastuzumab Fab 8 conjugation

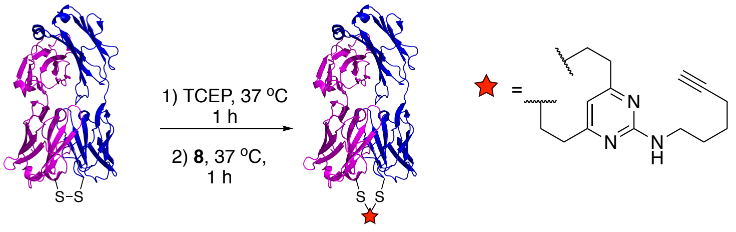

15

To a solution of trastuzumab Fab (10 µL, 27 µM, 1.28 mg/mL) in BBS (25 mM sodium borate pH 8, 25 mM NaCl, 0.5 mM EDTA) was added TCEP (5 eq.). The mixture was vortexed and incubated at 37 °C for 1 h. A solution of **8** (10 mM in DMSO) was added (final concentration of 270 µM, 10 eq.) and the reaction mixture incubated at 37 °C for 1 h. The excess reagents were removed by repeated diafiltration into fresh BBS using an Amicon-Ultra centrifugal filter (10000 MWCO, Merck Millipore). LC-MS analysis demonstrated >95% conversion to the desired conjugate.

a)

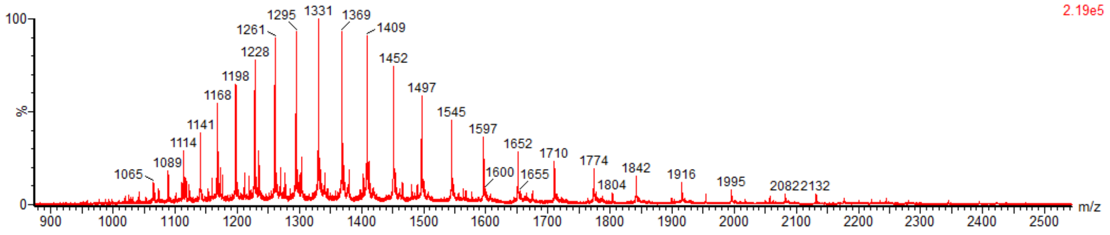

b)

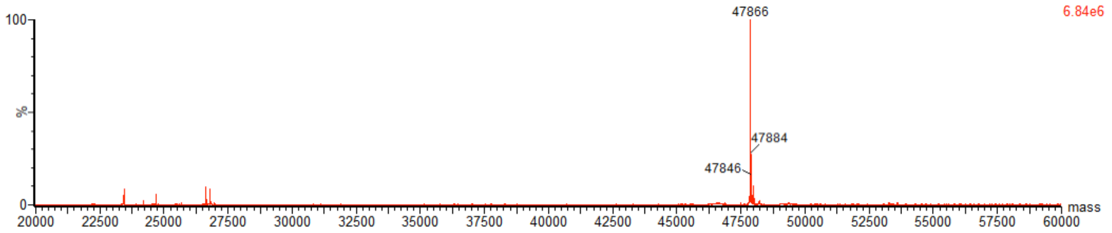

Fig. S12: LC-MS of reaction between trastuzumab Fab and **8**, a) non-deconvoluted MS and b) deconvoluted MS; expected 47,864 Da, observed 47,866 Da.

Trastuzumab Fab 9 conjugation

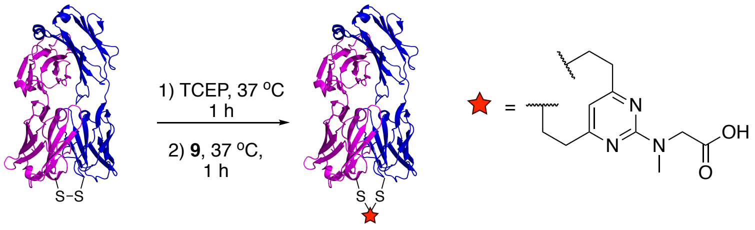

14

To a solution of trastuzumab Fab (10 µL, 27 µM, 1.28 mg/mL) in BBS (25 mM sodium borate pH 8, 25 mM NaCl, 0.5 mM EDTA) was added TCEP (5 eq.). The mixture was vortexed and incubated at 37 °C for 1 h. A solution of **9** (10 mM in DMSO) was added (final concentration of 270 µM, 10 eq.) and the reaction mixture incubated at 37 °C for 1 h. The excess reagents were removed by repeated diafiltration into fresh BBS using an Amicon-Ultra centrifugal filter (10000 MWCO, Merck Millipore). LC-MS analysis demonstrated >95% conversion to the desired conjugate.

a)

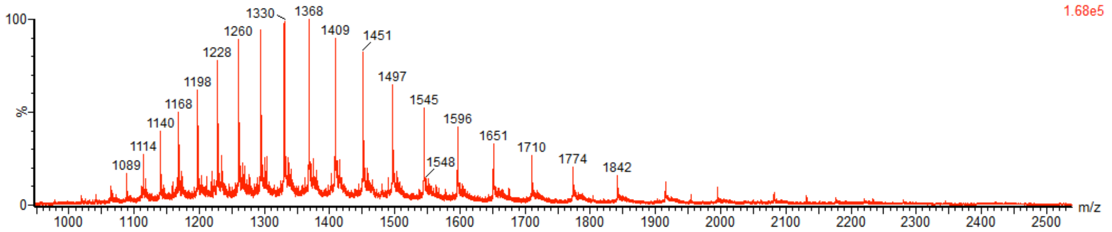

b)

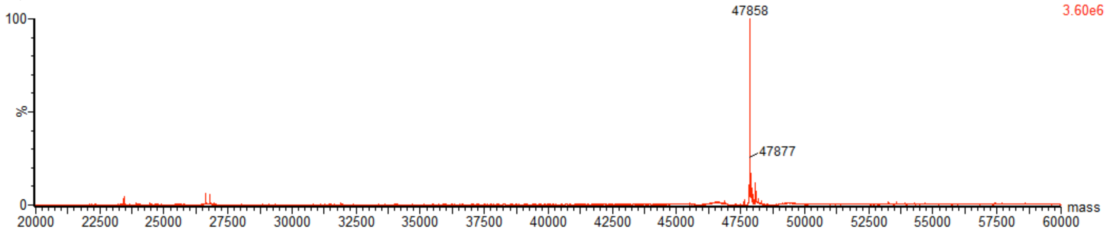

Fig. S13: LC-MS of reaction between trastuzumab Fab and **9**, a) non-deconvoluted MS and b) deconvoluted MS; expected 47,856 Da, observed 47,858 Da.

Trastuzumab Fab 8 conjugation kinetic experiment

To solutions of trastuzumab Fab (5 µL, 51 µM, 2.39 mg/mL) in BBS (25 mM sodium borate pH 8, 25 mM NaCl, 0.5 mM EDTA) was added TCEP (5 eq.). The mixtures were vortexed and incubated at 37 °C for 1 h. A solution of **8** (10 mM in DMSO) was added to each solution (final concentration equal to 10 and 20 equivalents) and the reaction mixture incubated at 37 °C. At t = 0, 5, 10, 15, 20, 30, 40 and 60 min, 0.5 µL was removed from each reaction, diluted with MilliQ water (9 µL) and quenched with 100 eq. of cysteine (5 mM in DMSO). LC-MS analysis was then used to quantify conversion.

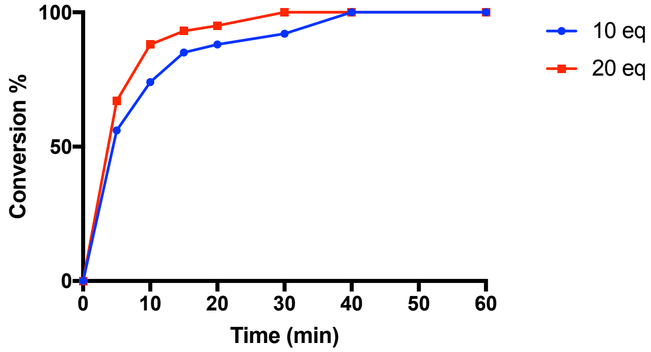

Fig. S14: Graph of bridging conversion of 10 or 20 equivalents of **8** with reduced trastuzumab Fab.

Trastuzumab 7 conjugation

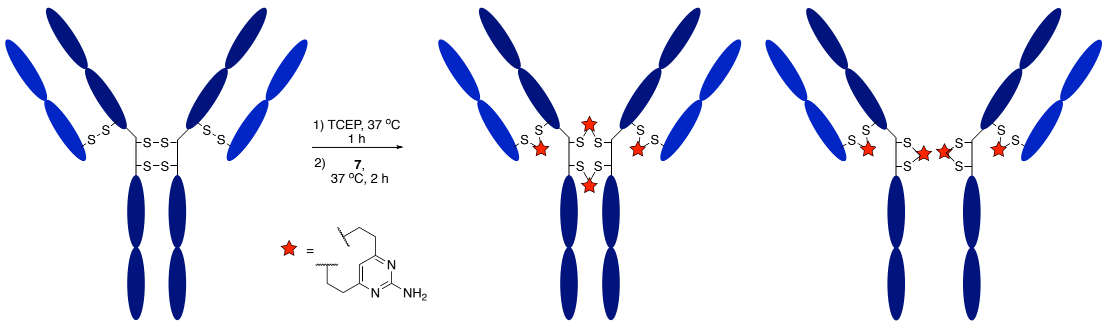

16

To a solution of trastuzumab (10 µL, 17 µM, 2.5 mg/mL) in Tris (25 mM Tris HCl pH 8, 25 mM NaCl, 0.5 mM EDTA) was added TCEP (10 eq.). The mixture was vortexed and incubated at 37 °C for 1 h. A solution of **7** (10 mM in DMSO) was added (final concentration of 680 µM, 40 eq.) and the reaction mixture incubated at 37 °C for 2 h. The excess reagents were removed by repeated diafiltration into PBS using an Amicon-Ultra centrifugal filter (10000 MWCO, Merck Millipore). LC-MS and SDS-PAGE analysis demonstrated >95% conversion to the bridged conjugate.

a)

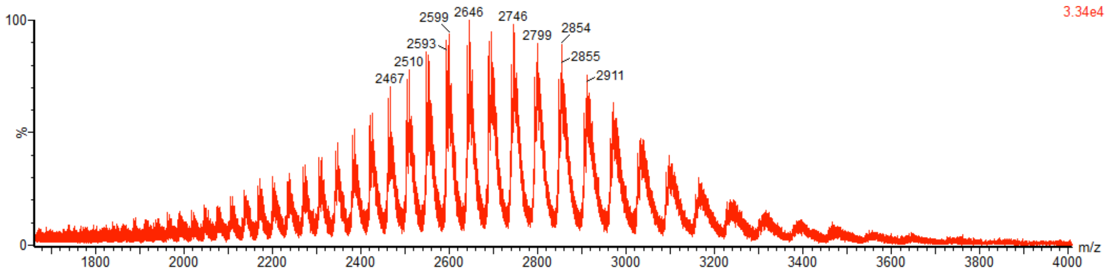

3.34e4
2599
2646
2746
2593
2799
2854
2510
2855
2467
2911
100
%
0
1800
2000
2200
2400
2600
2800
3000
3200
3400
3600
3800
4000
m/z

b)

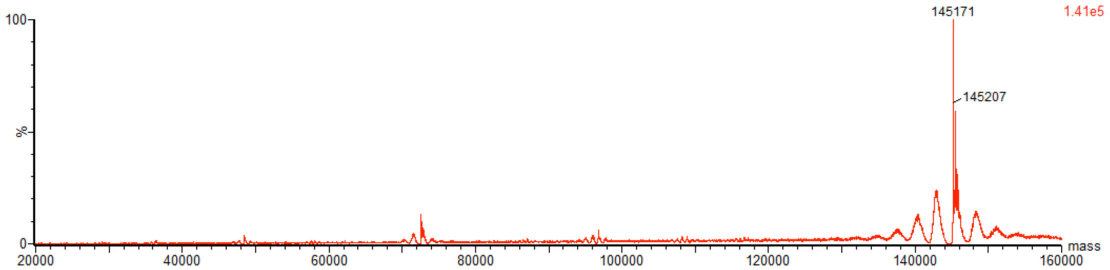

145171
1.41e5
145207
100
%
0
20000
40000
60000
80000
100000
120000
140000
160000
mass

c)

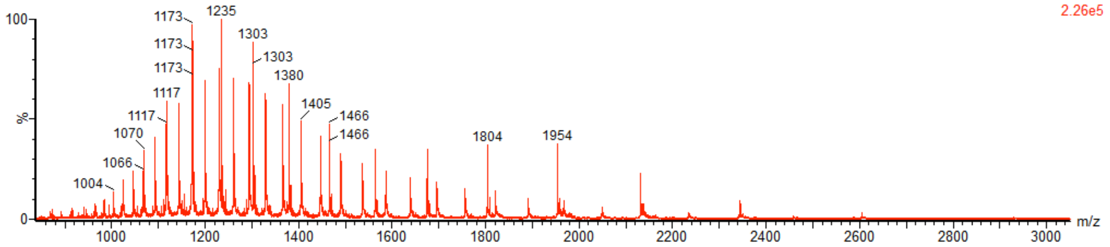

2.26e5
1173
1235
1173
1303
1173
1303
1380
1117
1405
1117
1466
1070
1466
1804
1954
1066
1004
100
%
0
1000
1200
1400
1600
1800
2000
2200
2400
2600
2800
3000
m/z

d)

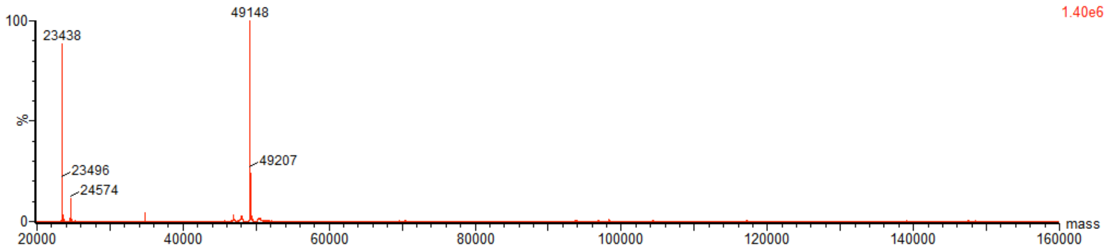

49148
1.40e6
23438
49207
23496
24574
100
%
0
20000
40000
60000
80000
100000
120000
140000
160000
mass

e)

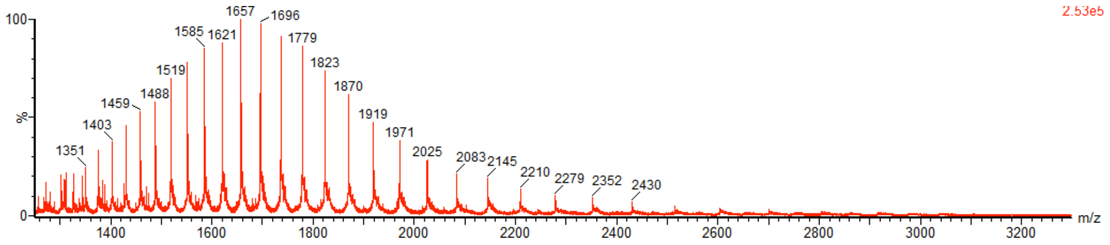

1657
2.53e5
1696
1585
1621
1779
1823
1519
1870
1488
1459
1919
1403
1971
1351
2025
2083
2145
2210
2279
2352
2430
100
%
0
1400
1600
1800
2000
2200
2400
2600
2800
3000
3200
m/z

f)

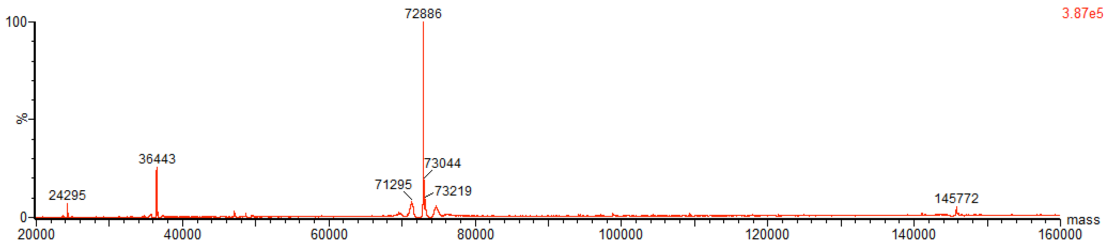

Fig. S15: Analysis of the reaction between trastuzumab and **7** by LC-MS, a) non-deconvoluted MS of trastuzumab, b) deconvoluted MS of trastuzumab, c) non-deconvoluted MS after TCEP reduction, d) deconvoluted MS after TCEP reduction; HC = 49,148 Da, LC = 23,438 Da, e) non-deconvoluted MS, f) by LC-MS, deconvoluted MS; expected 145,760, 72,880 Da, observed 145,772, 72,886 Da.

Trastuzumab 7 conjugation optimisation

Reactions were carried out as described in the previous section under the conditions described in Table S1. At time points of 1, 2, 4, 8 and 24 hours, aliquots were removed from the reactions, diluted with PBS, flash frozen and stored at -20 °C until analysis.

Table S1: Conditions tested in the optimisation of the rebridging reaction of trastuzumab with DVP **7**.

Entry	Conc. (mg/mL)	Temp. (°C)	Organic (%)	Linker (7) Eq.	Time (h)
**1**	2.5	37	DMSO (10)	10	1,2,4,8,24
**2**	2.5	37	DMSO (10)	20	1,2,4,8,24
**3**	2.5	37	DMSO (10)	40	1,2,4,8,24
**4**	2.5	37	DMSO (10)	60	1,2,4,8,24
**5**	2.5	37	DMSO (10)	100	1,2,4,8,24
**6**	2.5	37	DMSO (10)	200	1,2,4,8,24
**7**	2.5	37	DMSO (10)	500	1,2,4,8,24
**8**	1	37	DMSO (10)	40	1,2,4
**9**	5	37	DMSO (10)	40	1,2,4
**10**	7.5	37	DMSO (10)	40	1,2,4
**11**	2.5	37	DMSO (1)	40	1,2,4
**12**	2.5	37	DMSO (5)	40	1,2,4
**13**	2.5	37	DMSO (12)	40	1,2,4
**14**	2.5	37	DMSO (15)	40	1,2,4
**15**	2.5	25	DMSO (10)	40	1,2,4
**16**	2.5	4	DMSO (10)	40	1,2,4
**17**	2.5	37	DMF (10)	40	1,2,4
**18**	2.5	37	MeCN (10)	40	1,2,4
**19**	2.5	37	MeOH (10)	40	1,2,4
**20**[a]	2.5	37	DMSO (10)	40	1,2,4
**21**[a]	2.5	25	DMSO (10)	40	1,2,4
**22**[a]	2.5	4	DMSO (10)	40	1,2,4

[a]Linker (**7**) was added to trastuzumab and incubated for 10 min before the addition of TCEP (10 molar eq.).

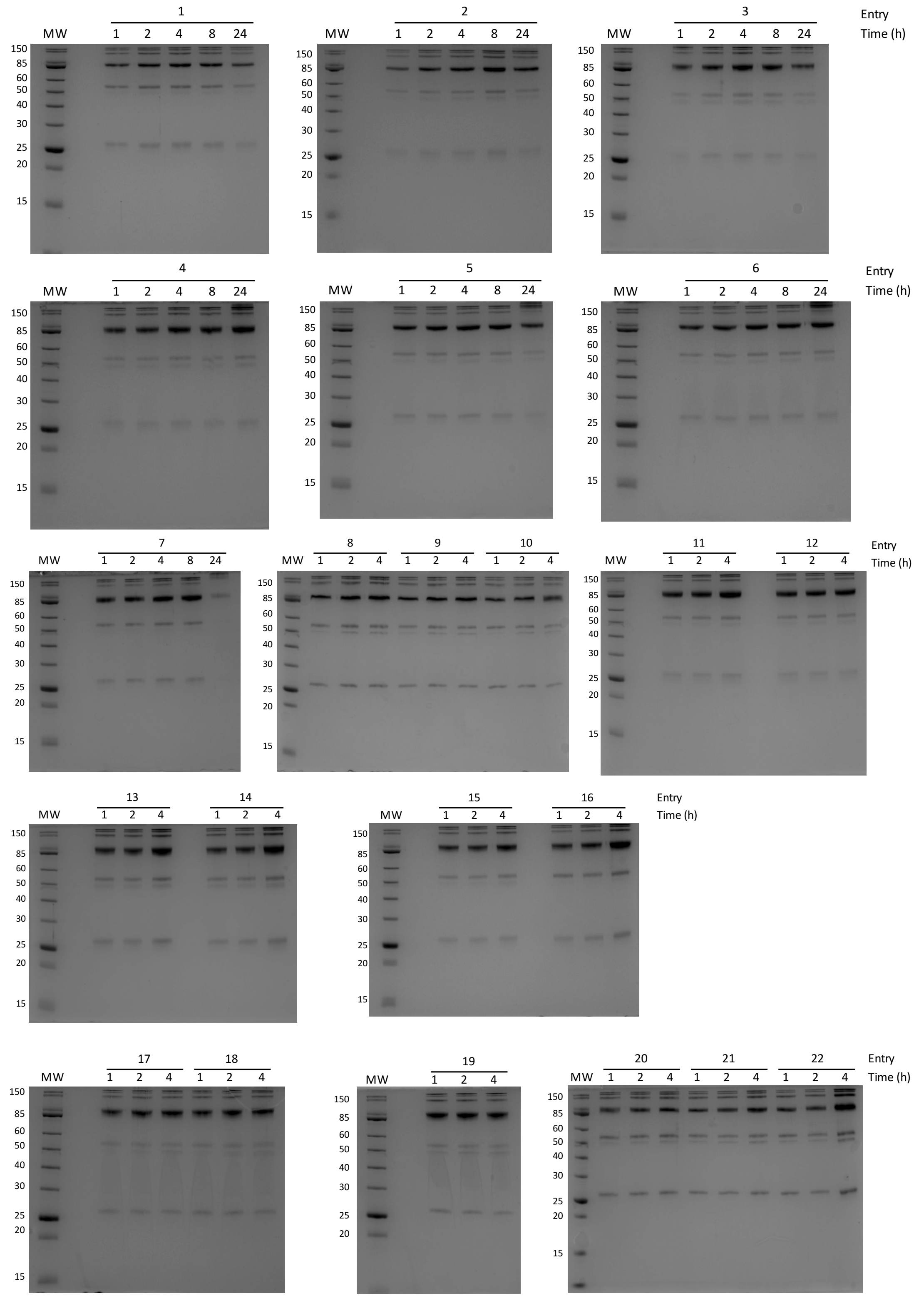

Fig. S16: SDS-PAGE analysis of the screen of conditions descrribed in Table S1. All samples were reduced with loading dye containing β-mercaptoethanol. The lanes are labelled according to the entry in Table S1 and the time the aliquot was removed from the reaction and frozen until analysis.

Trastuzumab 8 conjugation

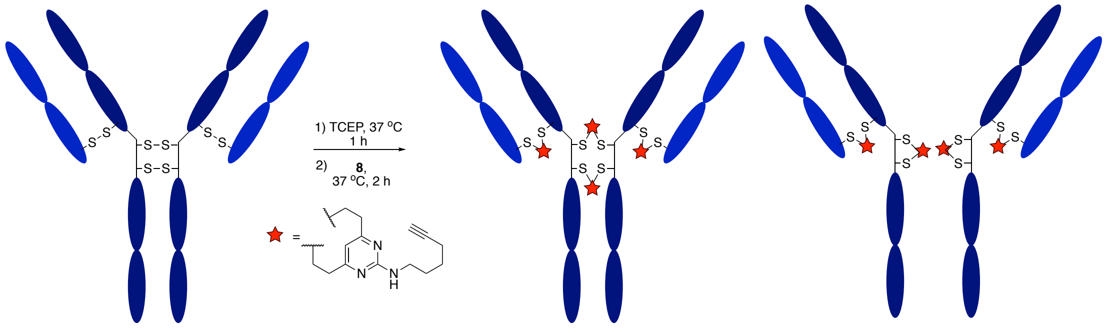

To a solution of trastuzumab (50 µL, 22.7 µM, 3.34 mg/mL) in Tris (25 mM Tris HCl pH 8, 25 mM NaCl, 0.5 mM EDTA) was added TCEP (10 eq.). The mixture was vortexed and incubated at 37 °C for 1 h. A solution of **8** (10 mM in DMSO) was added (final concentration of 908 µM, 40 eq.) and the reaction mixture incubated at 37 °C for 2 h. The excess reagents were removed by repeated diafiltration into PBS using an Amicon-Ultra centrifugal filter (10000 MWCO, Merck Millipore). LC-MS, SDS-PAGE and RP-HPLC analysis demonstrated >95% conversion to the bridged conjugate.

a)

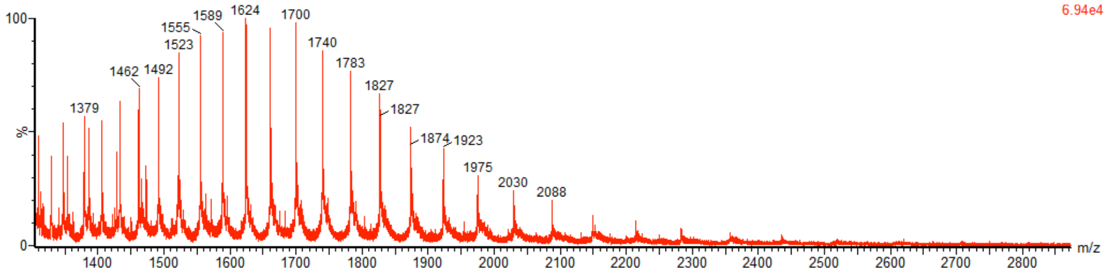

b)

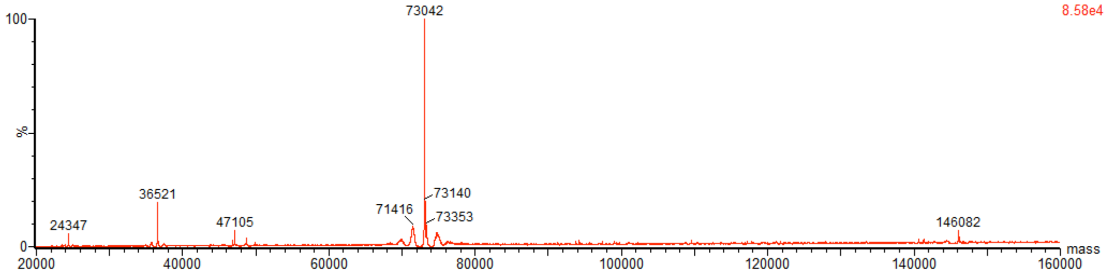

c)

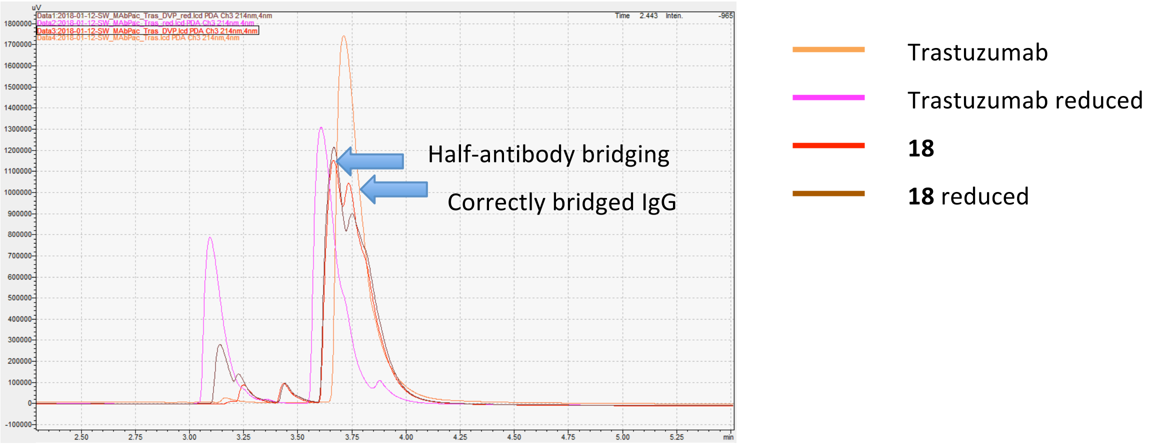

Product	Area
Light Chain	6.8%
Heavy Chain	2.6%
Half Antibody	41.5%
Full Antibody	49.1%

Fig. S17: Analysis of the reaction between trastuzumab and **8**, a) by LC-MS, non-deconvoluted MS, d) by LC-MS, deconvoluted MS; expected 146,080, 73,040 Da, observed 146,082, 73,042 Da, e) by RP-HPLC.

Trastuzumab 9 conjugation

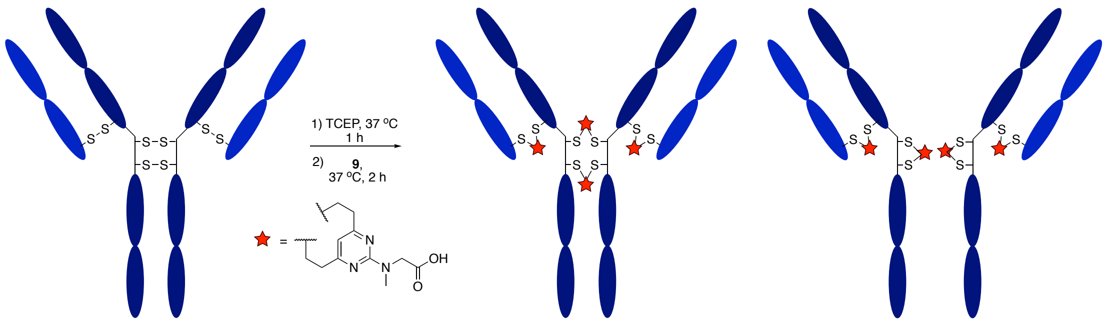

17

To a solution of trastuzumab (10 µL, 17 µM, 2.5 mg/mL) in Tris (25 mM Tris HCl pH 8, 25 mM NaCl, 0.5 mM EDTA) was added TCEP (10 eq.). The mixture was vortexed and incubated at 37 °C for 1 h. A solution of **9** (10 mM in DMSO) was added (final concentration of 680 µM, 40 eq.) and the reaction mixture incubated at 37 °C for 2 h. The excess reagents were removed by repeated diafiltration into PBS using an Amicon-Ultra centrifugal filter (10000 MWCO, Merck Millipore). LC-MS and SDS-PAGE analysis demonstrated >95% conversion to the bridged conjugate.

a)

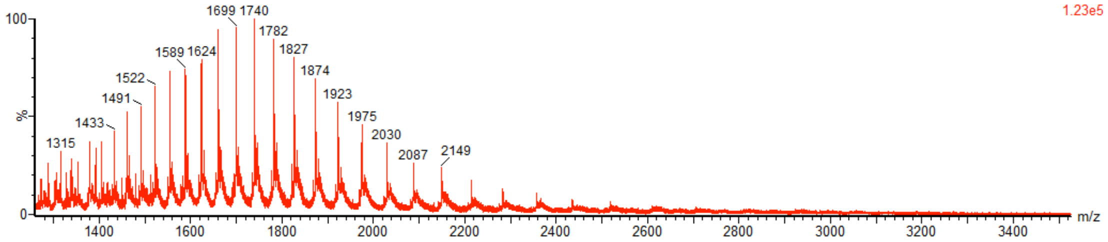

b)

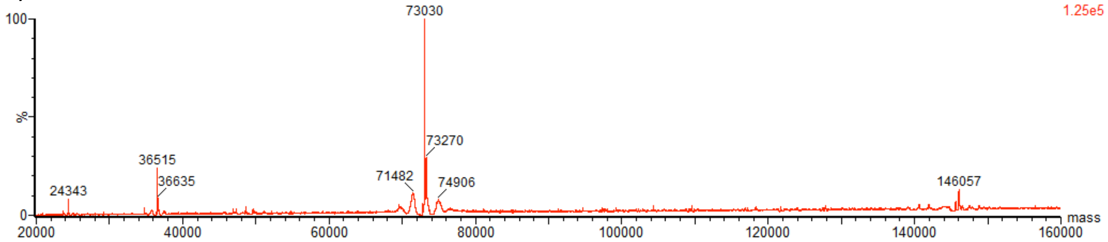

Fig. S18: Analysis of the reaction between trastuzumab and **9** by LC-MS, a) non-deconvoluted MS, d) by LC-MS, deconvoluted MS; expected 146,048, 73,024 Da, observed 146, 057, 73,030 Da.

Trastuzumab-DVP (18) Dox-PEG$_4$-N$_3$ CuAAC

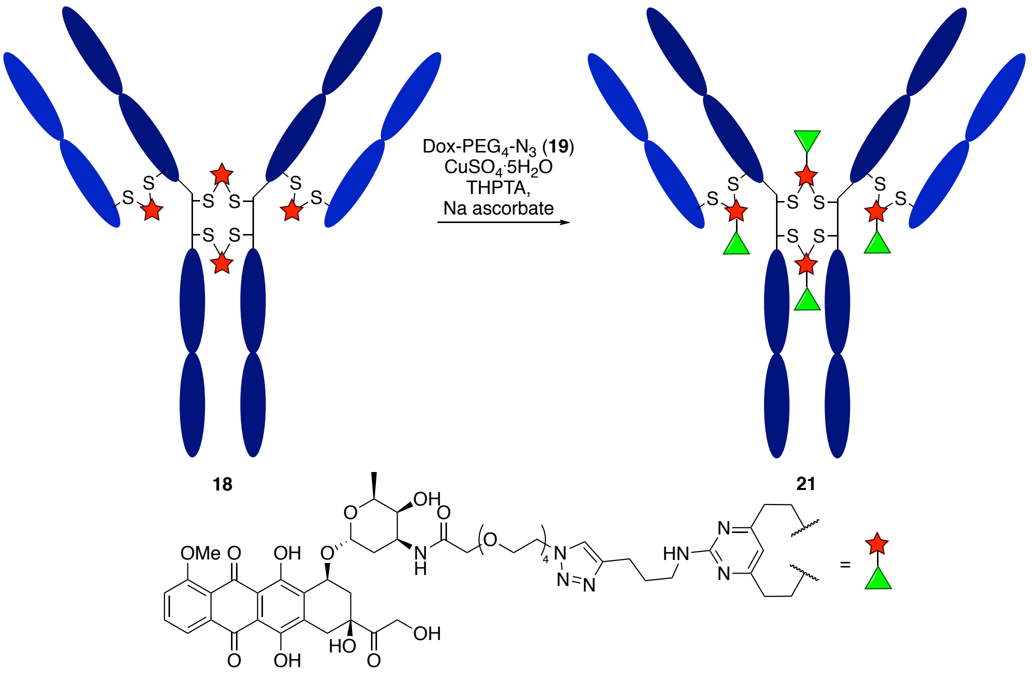

To a solution of trastuzumab-DVP **18** (70 µL, 12.5 µM, 1.86 mg/mL) in PBS was added **19** (5 mM in DMSO, to 150 µM), $CuSO_4{\cdot}5H_2O$ (to 250 µM), THPTA (to 1.25 mM) and sodium ascorbate (to 1.88 mM). The mixture was vortexed and incubated at 37 °C for 2 h. The excess reagents were removed by repeated diafiltration into PBS using an Amicon-Ultra centrifugal filter (10000 MWCO, Merck Millipore). LC-MS and UV-vis analysis revealed conversion to an ADC with an average DAR of 4.0.

a)

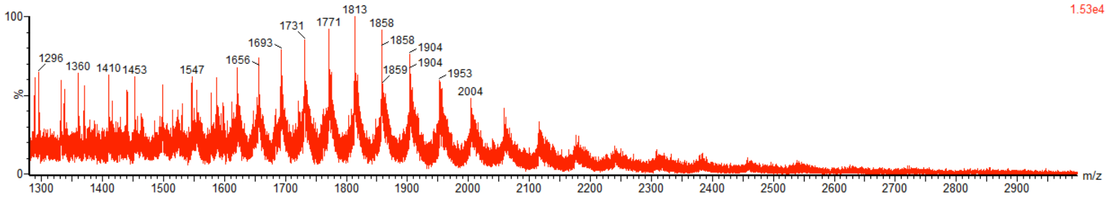

b)

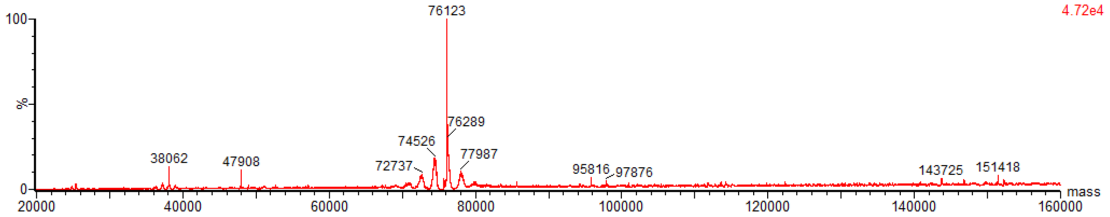

c)

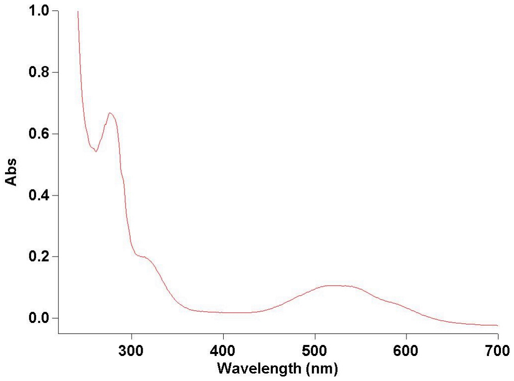

Fig. S19: LC-MS analysis of the CuAAC reaction between trastuzumab-DVP **18** and Dox-PEG$_4$-N$_3$ **19**, a) non-deconvoluted MS, b) deconvoluted MS; expected 76,119 Da, observed 76,123 Da, c) UV-visible absorbance of **21**.

UV-vis DAR Calculation

Sample buffer was used as a baseline for analysis. DAR was calculated using the following formula;

$$DAR = \frac{Abs_{495}/\varepsilon_{495}}{(Abs_{280} - 0.724 \times Abs_{495})/\varepsilon_{280}}$$

where;

Abs_{495} = 0.088

Abs_{280} = 0.656

ε_{280} = 215,380 M^{-1} cm^{-1} for trastuzumab

ε_{495} = 8030 M^{-1} cm^{-1} for doxorubicin

and a correction factor of 0.724 for doxorubicin absorption at 280 nm.

Trastuzumab-DVP (18) AlexaFluor488 Azide CuAAC

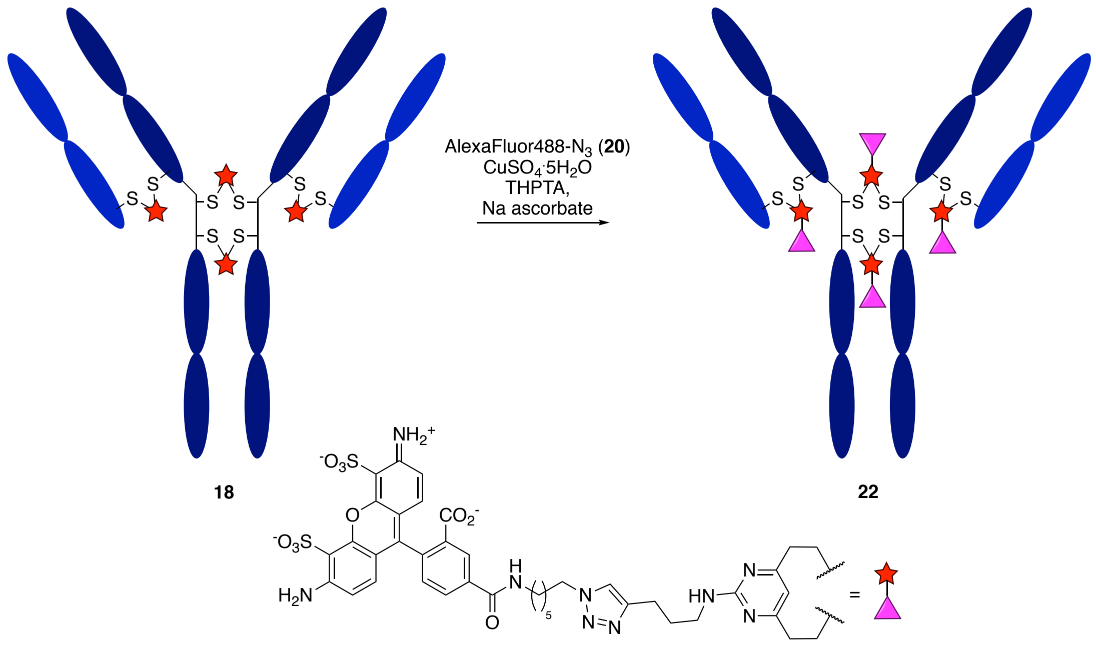

To a solution of trastuzumab-DVP **18** (55 µL, 13.7 µM, 2.05 mg/mL) in PBS was added AlexaFluor-488 Azide (ThermoFisher) (5 mM in DMSO, final concentration of 164.4 µM), $CuSO_4 \cdot 5H_2O$ (final concentration of 274 µM), THPTA (final concentration of 1.37 mM) and sodium ascorbate (final concentration of 2.06 mM). The mixture was vortexed and incubated at 37 °C for 4 h. The excess reagents were removed by repeated diafiltration into PBS using an Amicon-Ultra centrifugal filter (10000 MWCO, Merck Millipore). LC-MS and UV-vis analysis revealed conversion to an antibody-fluorophore conjugate (AFC) with an average fluorophore-antibody ratio (FAR) of 3.89.

a)

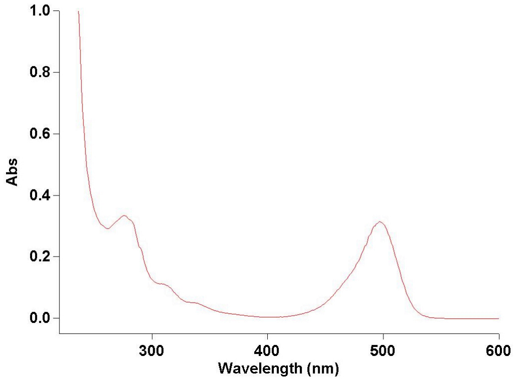

b)

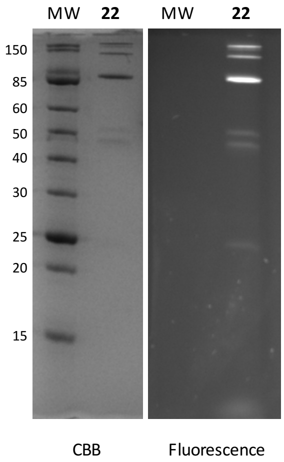

Fig. S20: a) UV-visible absorbance of trastuzumab conjugate, **22** and **b)** SDS-PAGE analysis of **22**.

UV-vis FAR Calculation

Sample buffer was used as a baseline for analysis. FAR was calculated using the following formula;

$$FAR = \frac{Abs_{495}/\varepsilon_{495}}{(Abs_{280} - 0.11 \times Abs_{495})/\varepsilon_{280}}$$

where;

Abs_{495} = 0.31

Abs_{280} = 0.25

ε_{280} = 215,380 M^{-1} cm^{-1} for trastuzumab

ε_{495} = 71,000 M^{-1} cm^{-1} for AlexaFluor488-Azide

and a correction factor of 0.11 for AlexaFluor488-Azide absorption at 280 nm.

Trastuzumab 24 conjugation

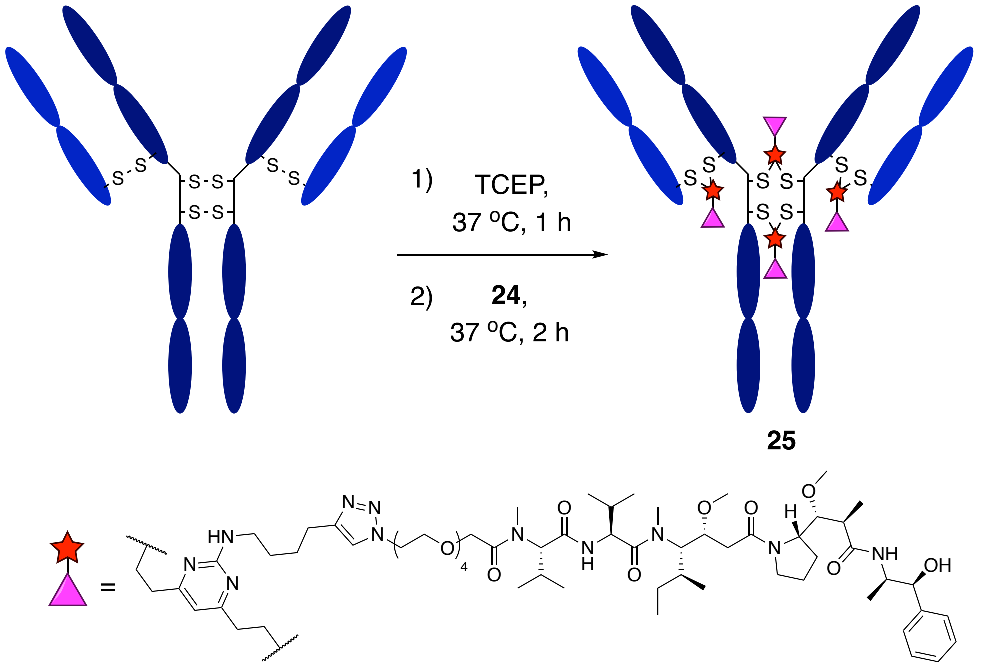

To a solution of trastuzumab (30 µL, 25.5 µM, 3.81 mg/mL) in Tris (25 mM Tris HCl pH 8, 25 mM NaCl, 0.5 mM EDTA) was added TCEP (10 eq.). The mixture was vortexed and incubated at 37 °C for 1 h. A solution of **24** (10 mM in DMSO) was added (final concentration of 1.02 mM, 40 eq.) and the reaction mixture incubated at 37 °C for 3 h. The excess reagents were removed by repeated diafiltration into PBS using an Amicon-Ultra centrifugal filter (10000 MWCO, Merck Millipore). LC-MS and SDS-PAGE analysis demonstrated >95% conversion to the half-antibody conjugate.

a)

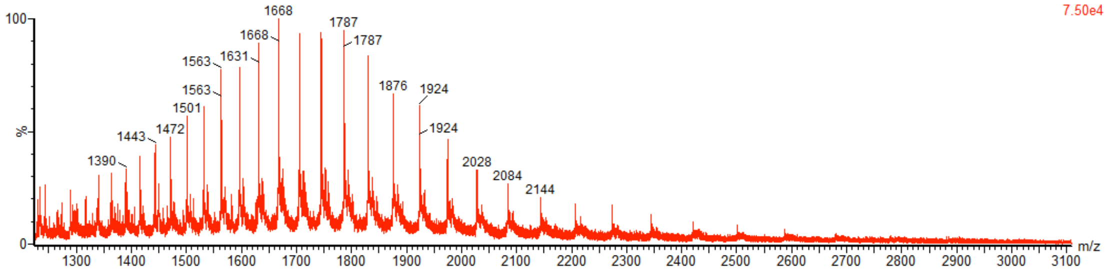

b)

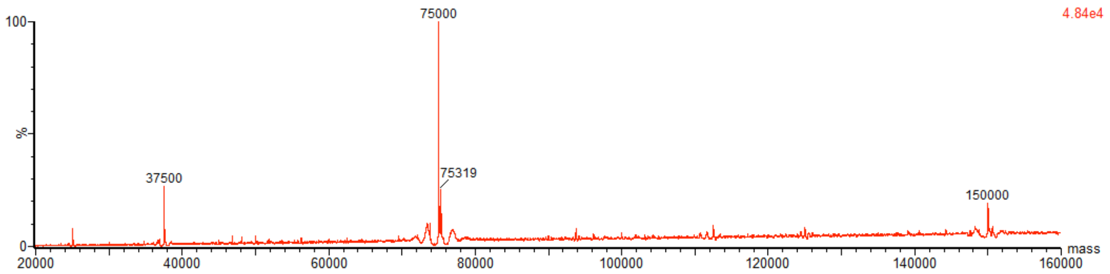

Fig. S21: LC-MS of reaction between trastuzumab and **24**, a) non-deconvoluted MS and b) deconvoluted MS; expected 149,988 and 74,994 Da; observed 150,000 and 75,000 Da.

Trastuzumab Ellman's Test

To a solution of trastuzumab (5 µL, 36.4 µM, 5.41 mg/mL) in Tris (25 mM Tris HCl pH 8, 25 mM NaCl, 0.5 mM EDTA) was added TCEP (10 eq.). The mixture was vortexed and incubated at 37 °C for 1 h. Excess TCEP was removed by repeated diafiltration into fresh Tris buffer using an Amicon-Ultra centrifugal filter (10000 MWCO, Merck Millipore) and concentrated to 1.35 µM (100 µL). To this solution was added 5,5'-dithiobis(2-nitrobenzoic acid) (DTNB) in 10% DMSO/Tris buffer (to 54 µM, 40 eq.) and the mixture incubated at room temperature for 10 min. UV-visible spectrometry revealed an absorption of 0.29 at 280 nm and 0.154 at 412 nm. The number of free sulfhydryls was calculated to be 8.06 using the following formula:

$$No. of\ free\ sulfhydryls = \frac{Abs_{412}/\varepsilon_{412}}{Abs_{280}/\varepsilon_{280}}$$

where;

ε_{412} = 14,150 M^{-1} cm^{-1}

ε_{280} = 215,389 M^{-1} cm^{-1}

Trastuzumab-AlexaFluor488 (22) Stability

A solution of trastuzumab-AlexaFluor488, **22** (70 µL, 2.10 µM) in PBS was diluted with 15 µL of reconstituted human plasma (Sigma) and 70 µL of PBS. To this solution was added reduced L-glutathione (final concentration = 1 µM) and mixture incubated at 37 °C for 14 days. Aliquots were removed after 0, 1, 3, 5, 7, 9, 11 and 14 days, flash frozen and stored at -20 °C until analysis. SDS-PAGE was followed by in-gel fluorescence and coomassie brilliant blue staining.

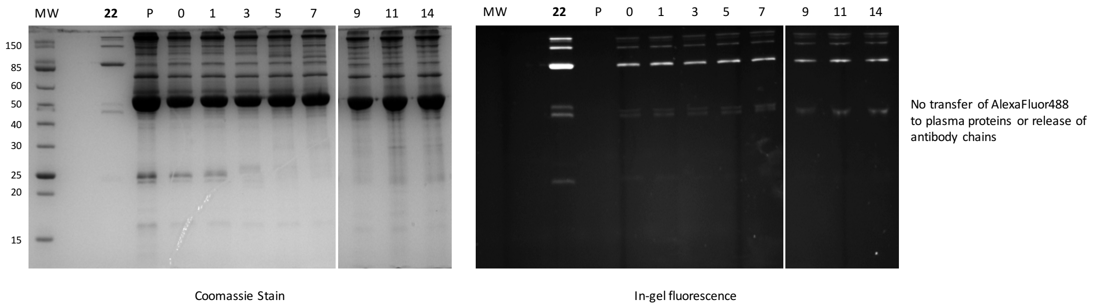

Fig. S22: SDS-PAGE analysis of stability. The left gel shows commassie staining of the gel, the right gel shows in gel fluorescence which was recorded prior to staining. The numbers above lanes refer to the days of **22** incubation in human plasma. M = molecular weight marker, P = human plasma.

Cysteine selectivity

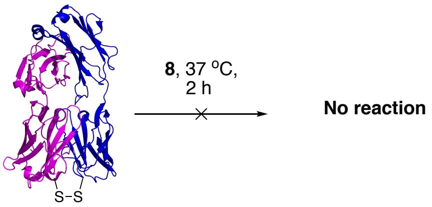

To a solution of trastuzumab Fab (5 µL, 29.6 µM, 1.41 mg/mL) in BBS (25 mM sodium borate pH 8, 25 mM NaCl, 0.5 mM EDTA) was added solution of **8** (10 mM in DMSO, final concentration of 296 µM, 10 eq.). The reaction mixture was vortexed and incubated at 37 °C for 2 h. LC-MS analysis revealed that no reaction had occurred.

a) T = 0h

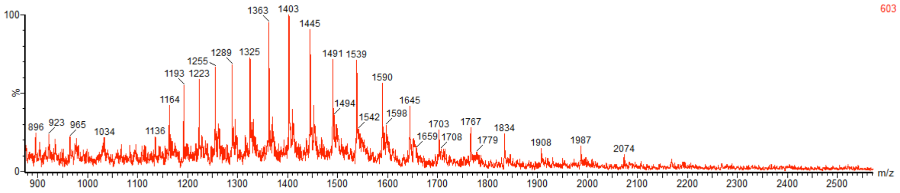

b) T = 0h

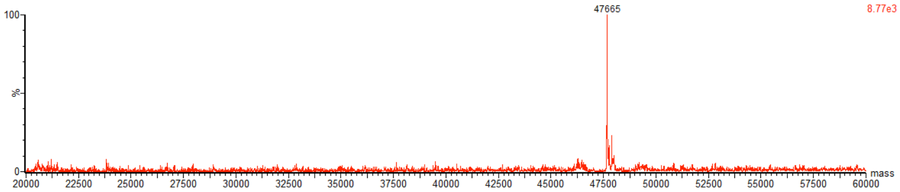

c) T = 2h

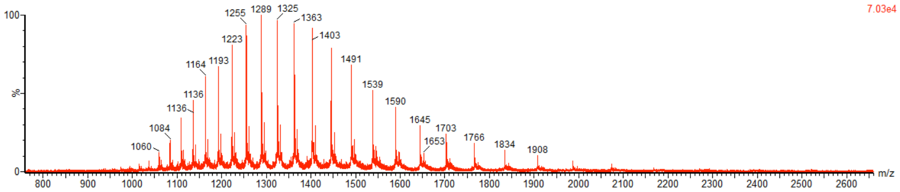

d) T = 2h

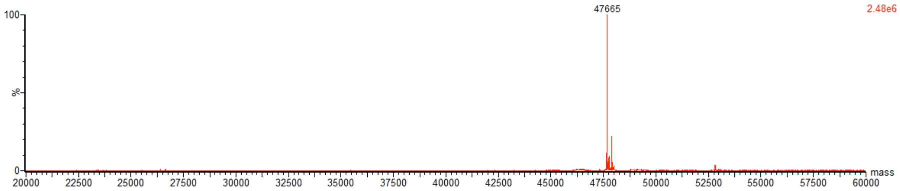

Fig. S23: LC-MS of reaction between trastuzumab Fab and **8** in the absence of TCEP, a) non-deconvoluted MS and b) deconvoluted MS at T = 0h, c) non-deconvoluted MS and b) deconvoluted MS at T = 2h; expected 47,665 Da, observed 47,665 Da.

Enzyme-linked immunosorbent assay (ELISA)

A 96-well plate was coated with 100 µL of a 0.25 µg/mL solution of HER2 (Sino Biological, His-tagged) overnight at 4 °C. Coating solutions were removed and each well washed with PBS (2 × 200 µL). Each well was then blocked with 1% BSA in PBS (200 µL) for 1 h at room temperature. The blocking solution was then removed and each well washed with PBS (3 × 200 µL). Wells were treated with a serial dilution of trastuzumab and trastuzumab-DVP conjugates **16**, **17** and **18** in PBS (100 µL of 30 nM, 10 nM, 3.33 nM, 1.11 nM, 0.37 nM, 0.12 nM, 0 nM) and incubated at room temperature for 2 h. The conjugate solutions were removed and each well was washed with 0.1% Tween 20 in PBS (2 × 200 µL) followed by PBS (3 × 200 µL). Next, 100 µL of detection antibody (1:1000 dilution of a mouse anti-human IgG-HRP, ThermoFisher) in PBS was added to each well and incubated at room temperature for 1 h. Each well was washed with 0.1% Tween 20 in PBS (2 × 200 µL) followed by PBS (3 × 200 µL). Finally, an OPD solution (100 µL of a solution prepared by dissolving 1 capsule in 9 mL H_2O and 1 mL stable peroxide substrate buffer (10×), ThermoFisher) was added to each well. After 10-15 minutes, 4M $HCl_{(aq)}$ (50 µL) was added to each well to quench the reaction. Absorbance was measured at 490 nm and 590 nm. Measurements were performed in quadruplicate and three independent repeats were performed.

Cells Lines

HER2-positive SKBR3 and BT474 cells were obtained from the American Type Culture Collection (ATCC) and HER2-negative MCF7 and T47D cells were obtained from the

European Collection of Authenticated Cell Cultures (ECACC) and ATCC, respectively. SKBR3 cells were maintained in high glucose McCoy's 5A medium, supplemented with 10% heat-inactivated foetal-bovine serum (FBS), 50 U/mL penicillin and 50 µg/mL streptomycin. MCF7 cells were maintained in Dulbecco's Modified Eagle Medium (DMEM) supplemented with 10% heat-inactivated fetal-bovine serum (FBS), 2 mM L-glutamine, 50 U/mL penicillin and 50 µg/mL streptomycin. BT474 and T47D cell lines were maintained in RPMI1640 medium supplemented with 10% heat-inactivated fetal-bovine serum (FBS), 2 mM L-glutamine, 50 U/mL penicillin and 50 µg/mL streptomycin. All cell lines were incubated at 37 °C with 5% CO_2.

Live Cell Labeling/Internalization by Fluorescence-activated cell sorting (FACS)

SKBR3, BT474, MCF7 and T47D cell lines were seeded in 6-well plates at 10^6 cells/well and allowed to adhere for 24 h at 37 °C with 5% CO_2. Cells were treated with 50 nM **22**, AlexaFluor™488 azide or PBS in complete growth medium for 1 h at 37 °C. Next, growth medium was removed, the cells were washed with PBS to remove unbound antibodies and the cells were detached with Accutase® cell dissociation reagent (StemPro), washed with PBS, pelleted and resuspended in PBS (100 µL). Analysis was conducted on an Amnis® ImageStream® imaging flow cytometer (Merck Millipore). DRAQ5™ (ThermoFisher) was used as a nuclear stain.

Cell Viability

Cells were seeded in 96-well plates for 24 h at 37 °C with 5% CO_2. SKBR3 cells were seeded at 15,000 cells/well, BT474 cells were seeded at 20,000 cells/well, MCF7 cells were seeded at 7,500 cells/well and T47D cells were seeded at 10,000 cells/well. Serial dilutions of **25**, trastuzumab and MMAE were added to the cells in complete growth medium and incubated at 37 °C with 5% CO_2 for 96 h. Cell viability was measured using CellTiter-Glo viability assay (Promega) according to the manufacturer's instructions. Cell viability was plotted as a percentage of untreated cells. Each measurement was taken in triplicate and three independent repeats were performed.

Cell Growth Assay

Cells were seeded in 96-well plates for 24 h at 37 °C with 5% CO_2. SKBR3 cells were seeded at 15,000 cells/well, BT474 cells were seeded at 20,000 cells/well, MCF7 cells were seeded at 7,500 cells/well and T47D cells were seeded at 10,000 cells/well. Serial dilutions of **25** were added to the cells in complete growth medium and incubated at 37 °C with 5% CO_2 for 6 days in an IncuCyte®. Cell growth is given as percentage confluence. Each concentration was measured in triplicate and error bars indicate standard deviation.

a)

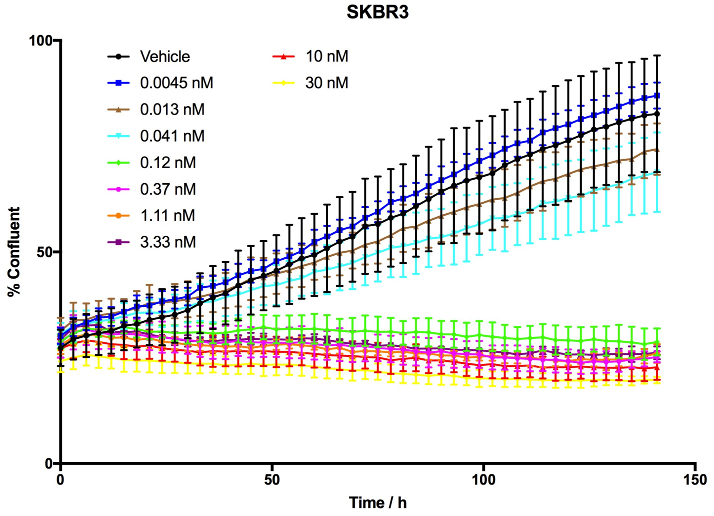

b)

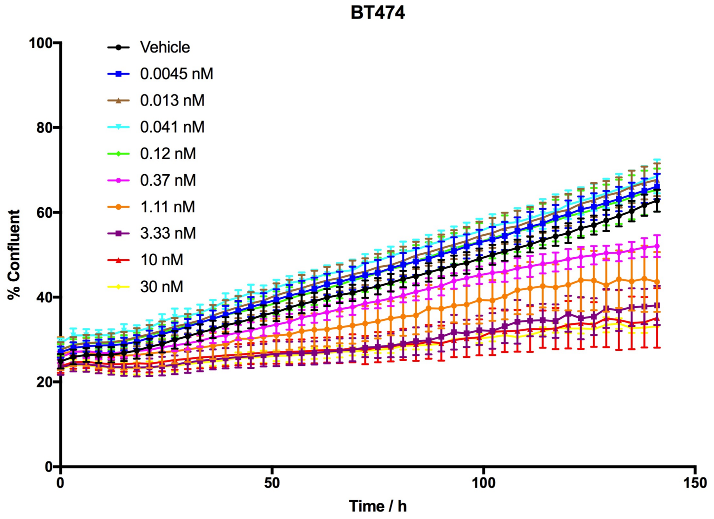

c)

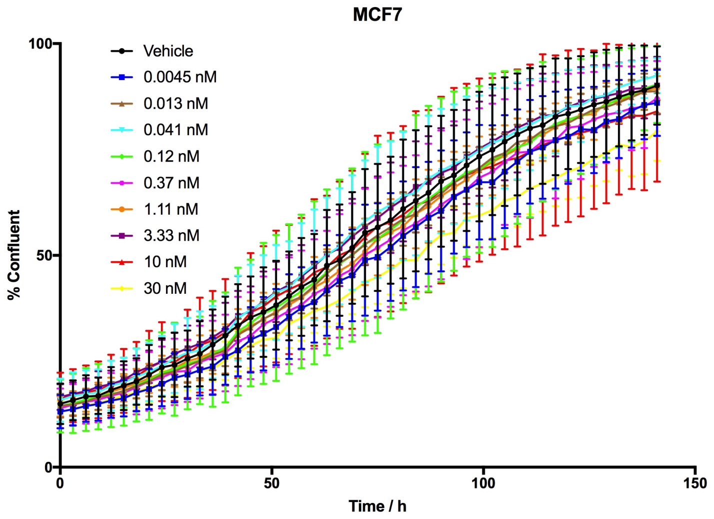

d)

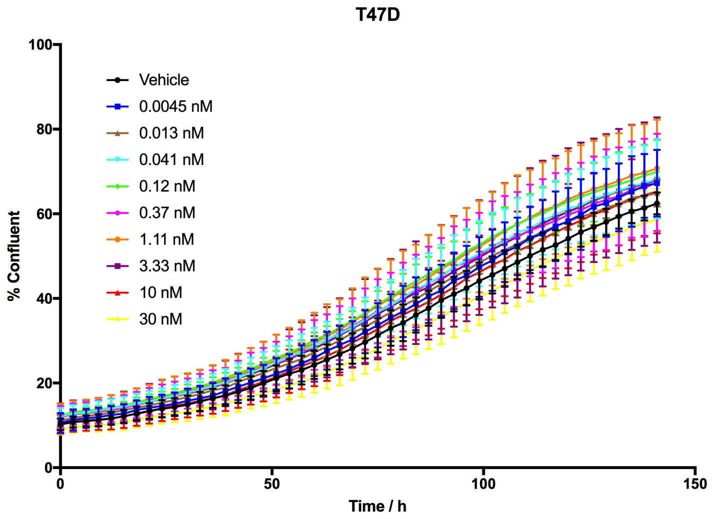

Fig. S24: Cell growth curves after incubation of **25** for 6 days in **a)** SKBR3, **b)** BT474, **c)** MCF7 and **d)** T47D cell lines.

*Pf*RadA Sequence and SDS-PAGE

a)

ATIGRISTGSKSLDKLLGGGIETQAITEVFGEFGSGKTQLAHTLAVMVQLPPEEGGL

NGSVIWIDTENTFRPERIREIAQ**C**RGLDPDEVLKHIYVARAFNSNHQMLLVQQAEDK

IKELLNTDRPVKLLIVDSLTSHFRSEYIGRGALAERQQKLAKHLADLHRLANLYDIA

VFVTNQVQANGGHILAHSATLRVYLRKGKGGKRIARLIDAPHLPEGEAVFSIT**C**KGI

ED

Molecular Weight = 25,304 Da.

b)

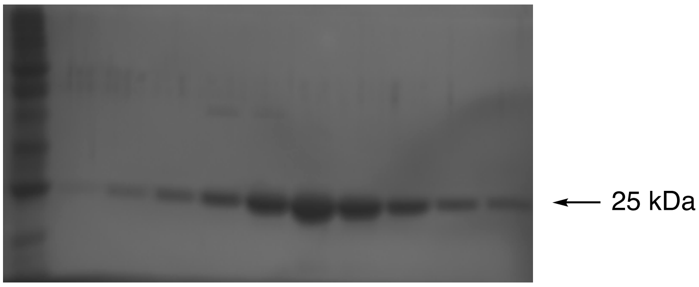

Fig. S25: a) Amino acid sequence of *Pf*RadA-dCys, mutations at positions 188 and 344 are highlighted in bold and underlined and **b)** SDS-PAGE analysis of purified recombinant *Pf*RadA-dCys.

NMR Spectra

2-amino-4-vinyl-pyrimidine (1)

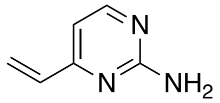

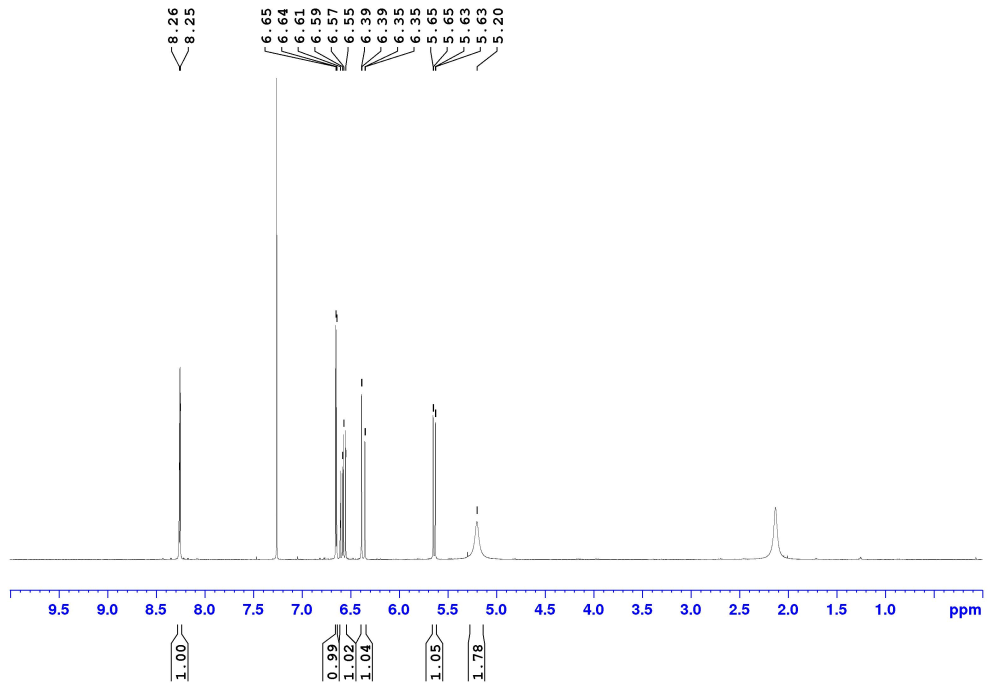

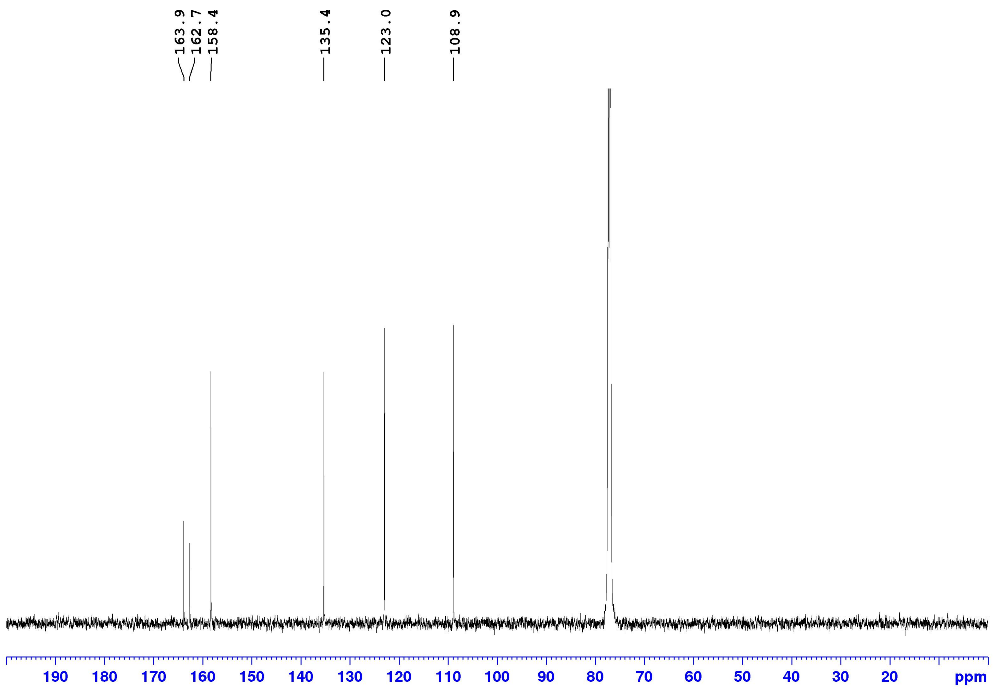

Methyl *S*-(2-(2-aminopyrimidin-4-yl)ethyl)-*N*-*tert*-butoxycarbonyl)-*L*-cysteinate (3)

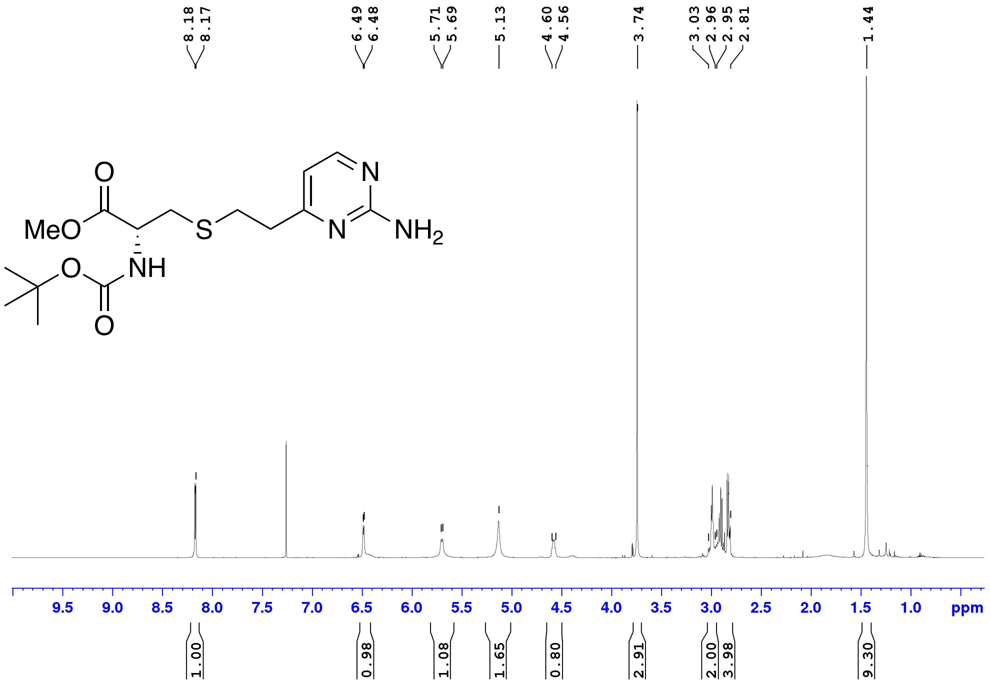

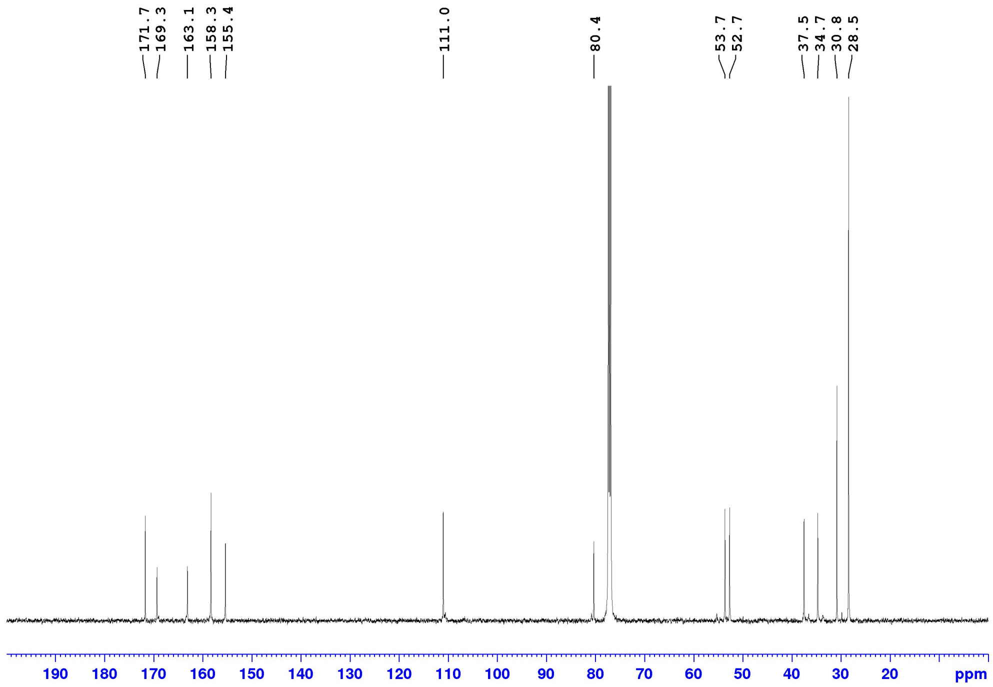

Methyl *S*-(1-benzyl-2,5-dioxopyrrolidin-3-yl)-*N*-(*tert*-butoxycarbonyl)-*L*-cysteinate (6)

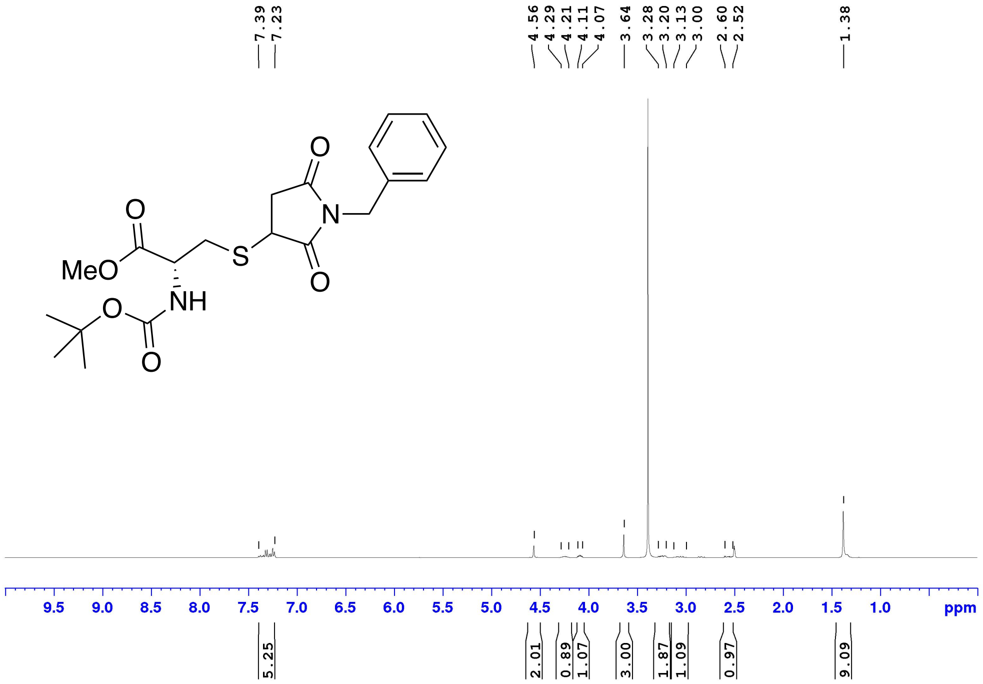

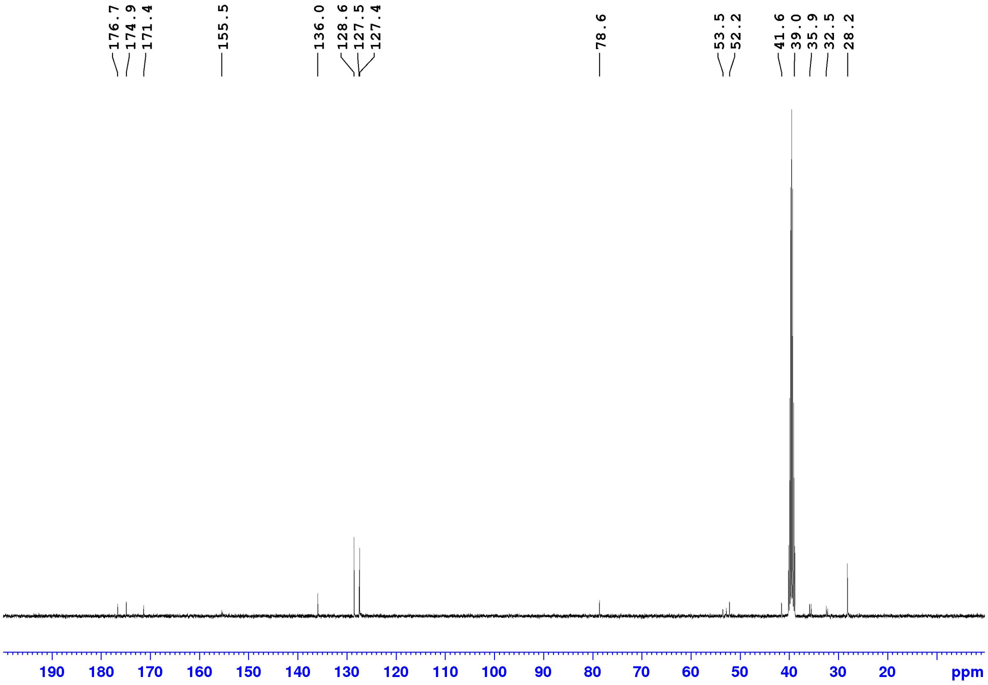

2-amino-4,6-divinylpyrimidine (7)

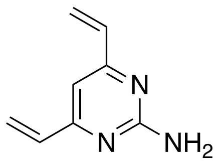

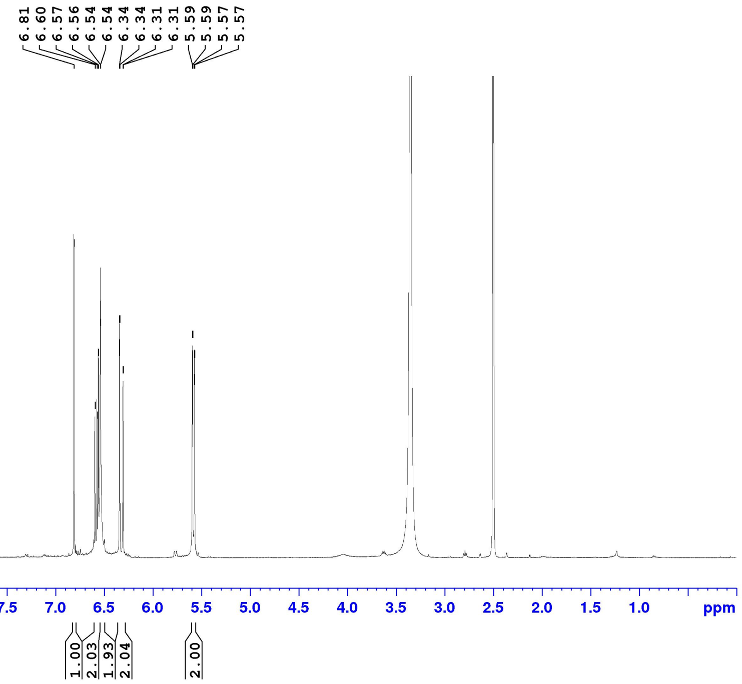

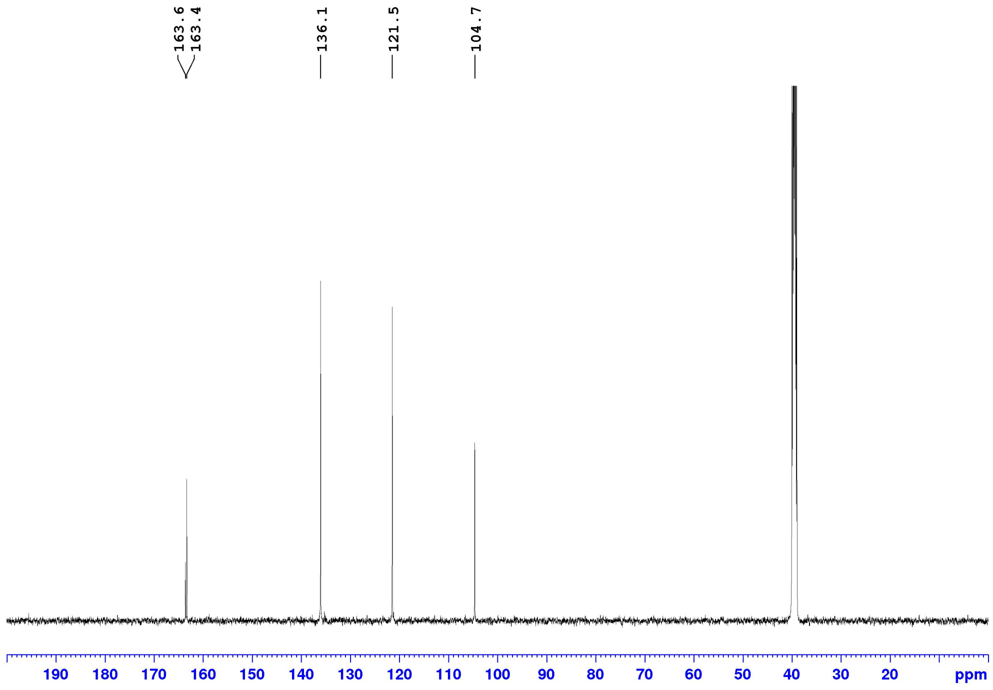

N-(hex-5-yn-1-yl)-4,6-divinylpyrimidin-2-amine (8)

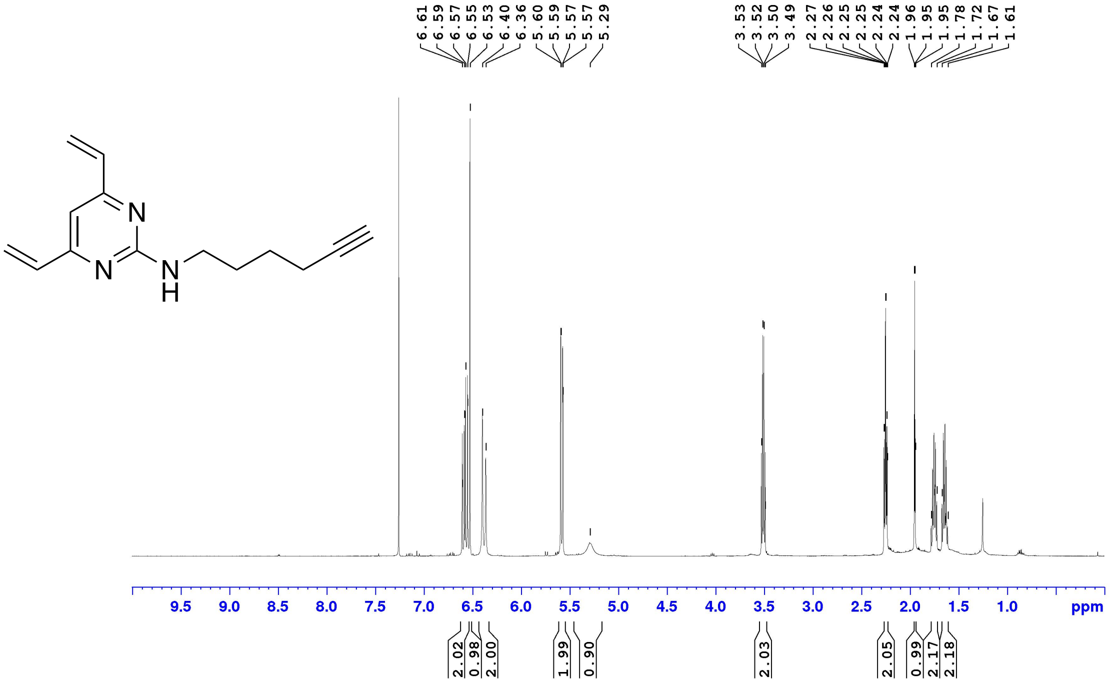

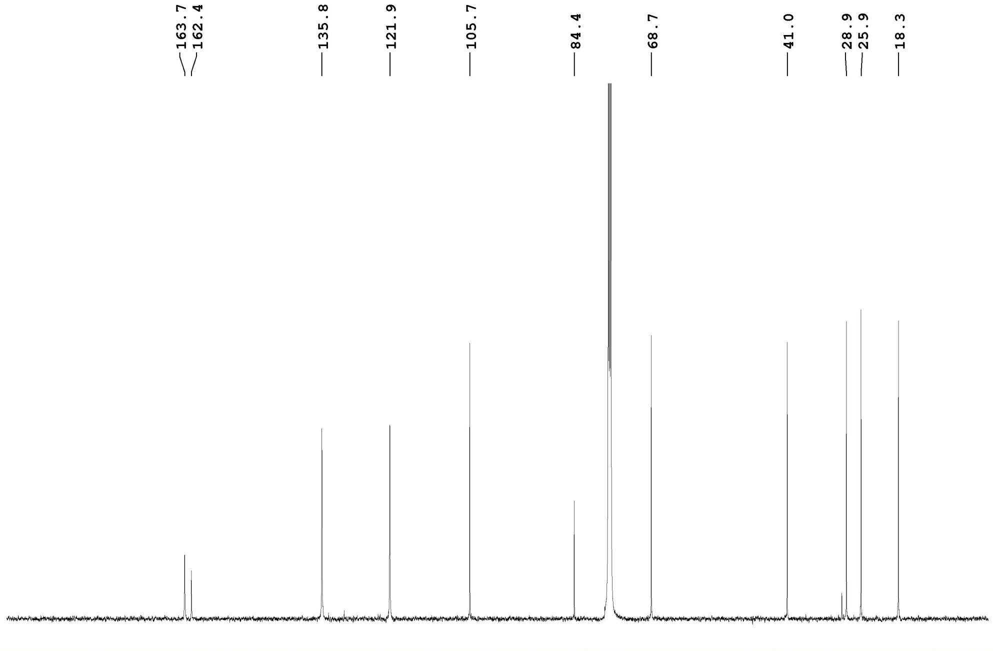

Ethyl *N*-(4,6-dichloropyrimidin-2-yl)-*N*-methylglycinate (26)

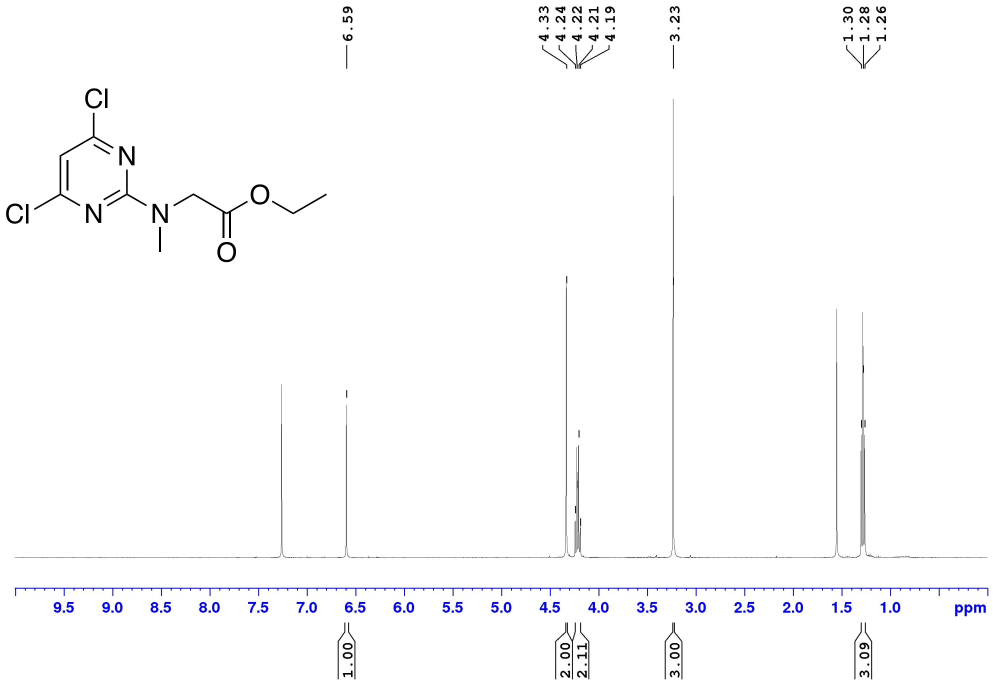

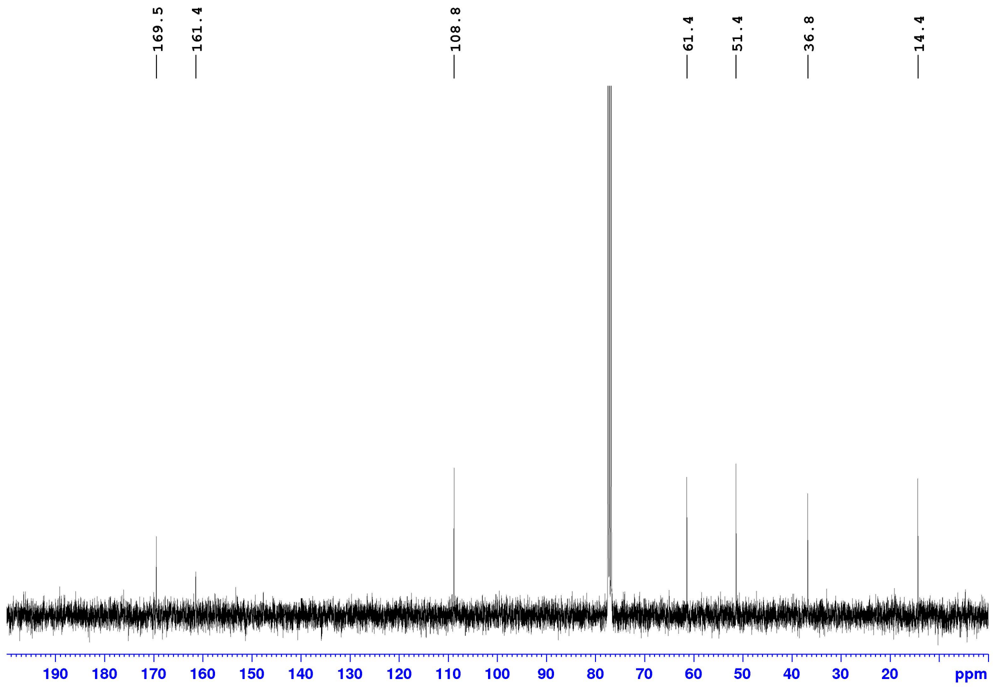

Ethyl *N*-(4,6-divinylpyrimidin-2-yl)-*N*-methylglycinate (27)

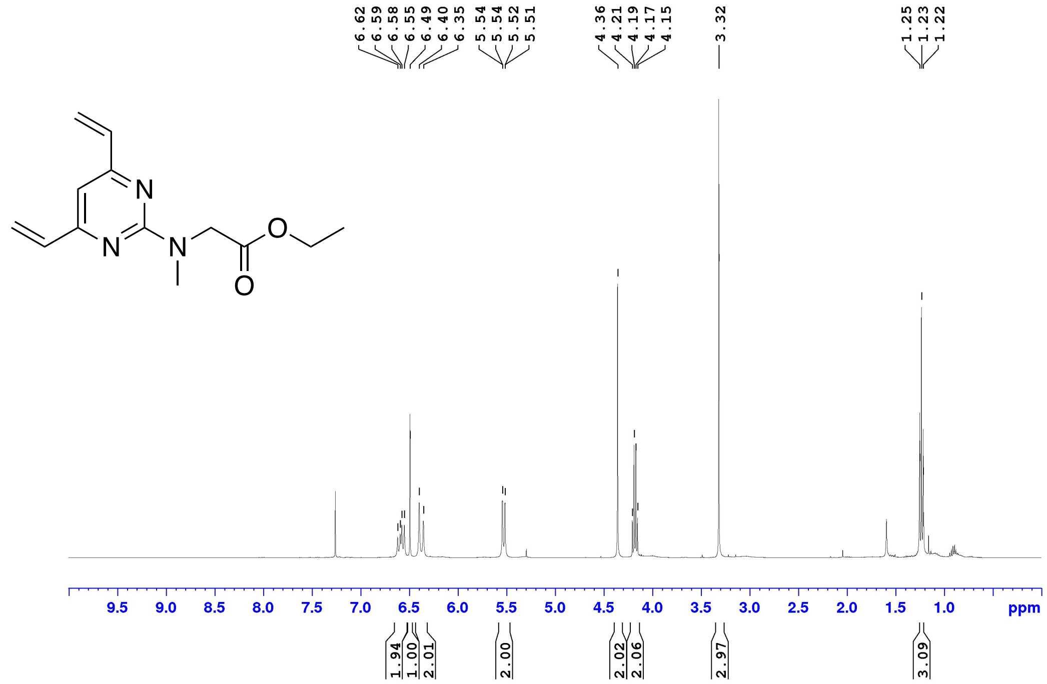

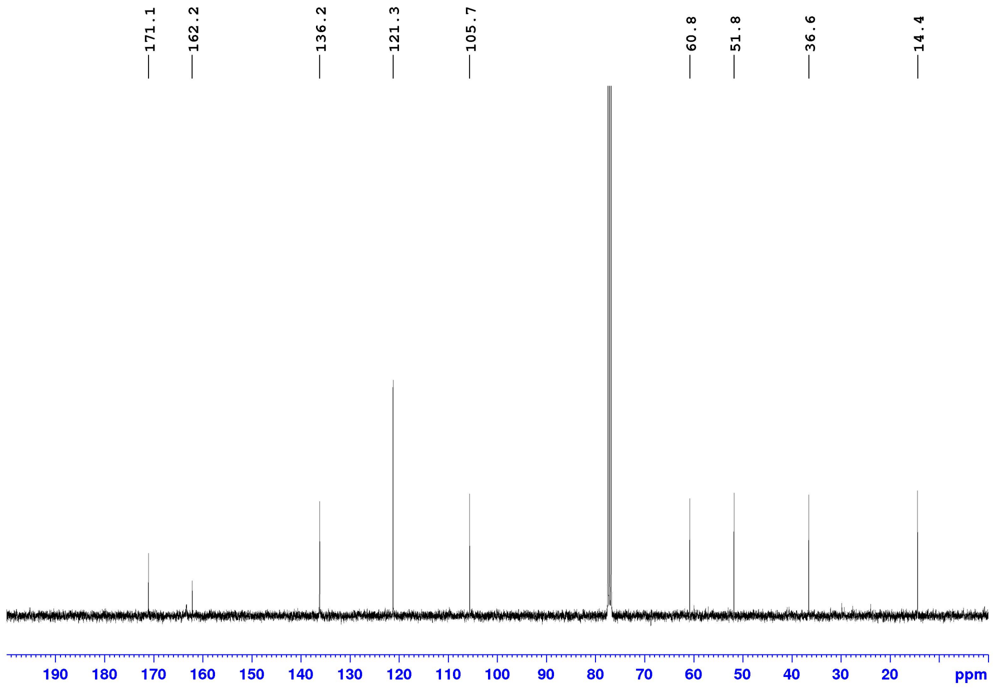

_N_-(4,6-divinylpyrimidin-2-yl)-_N_-methylglycine (9)

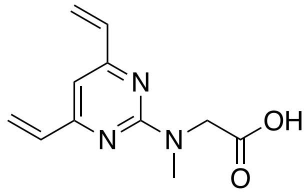

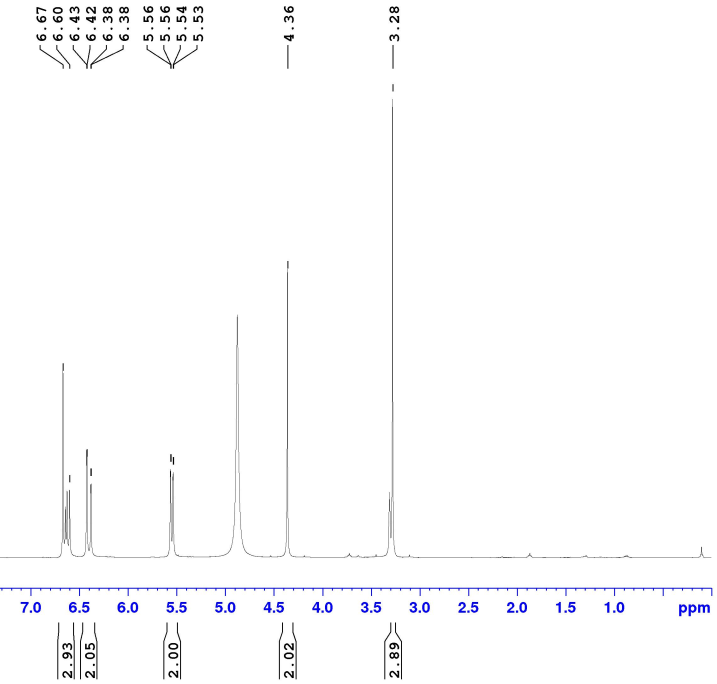

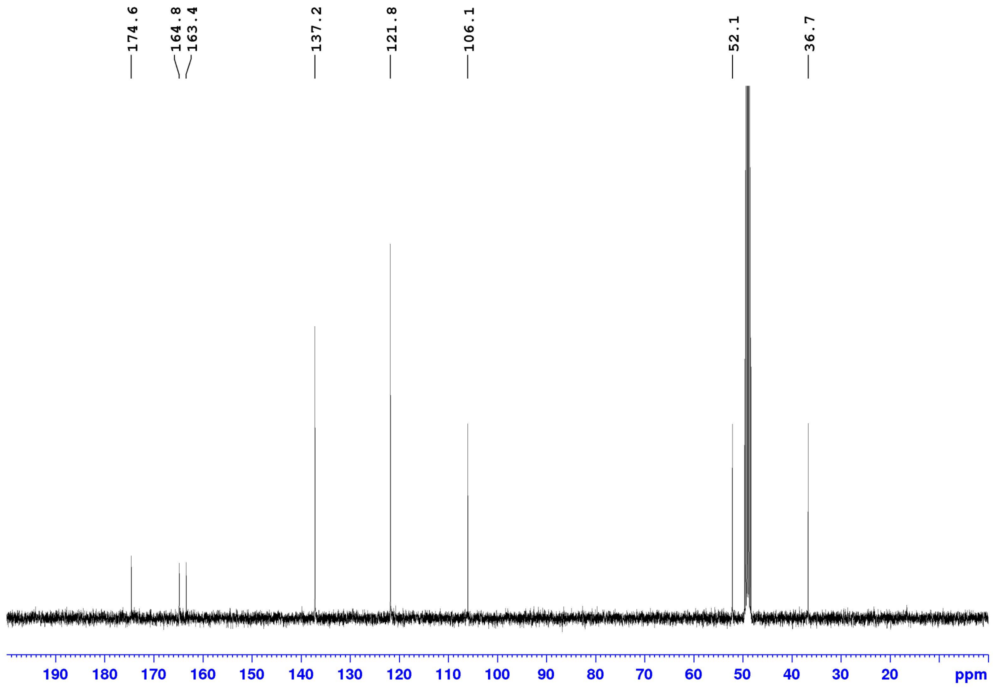

Dox-PEG$_4$-N$_3$ (19)

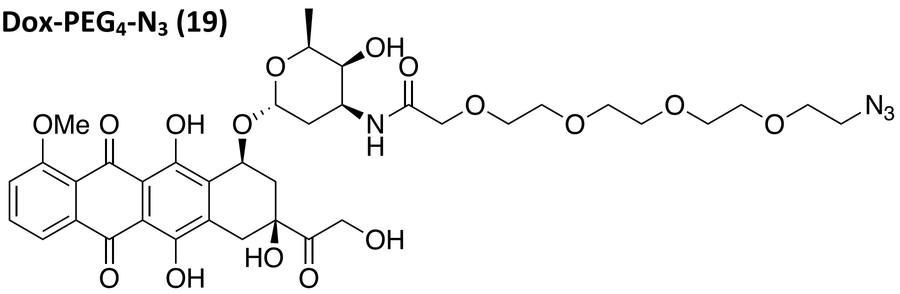

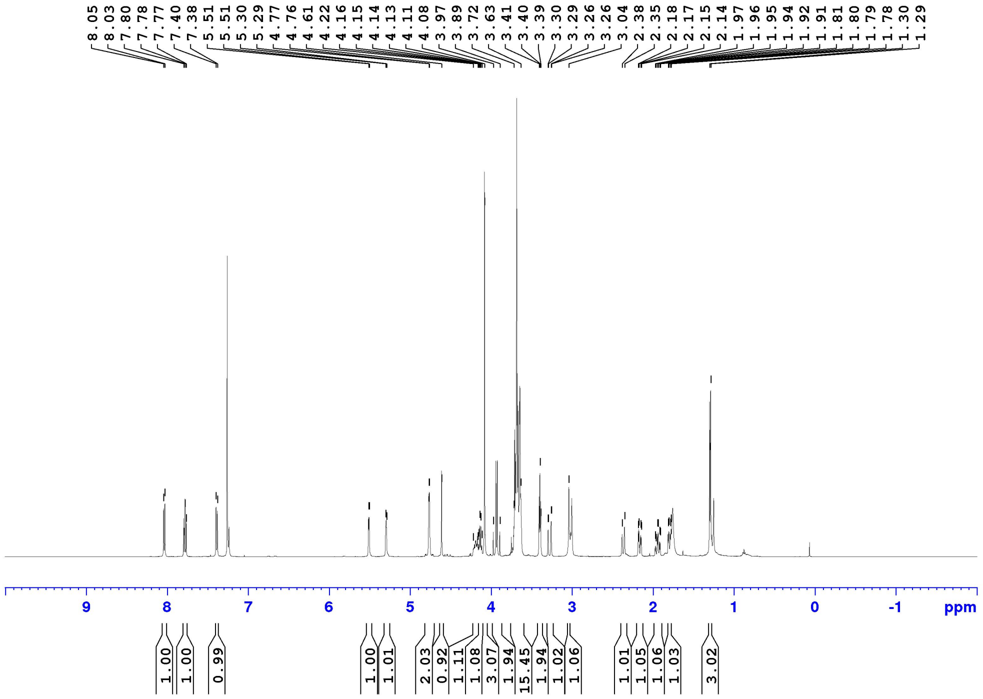

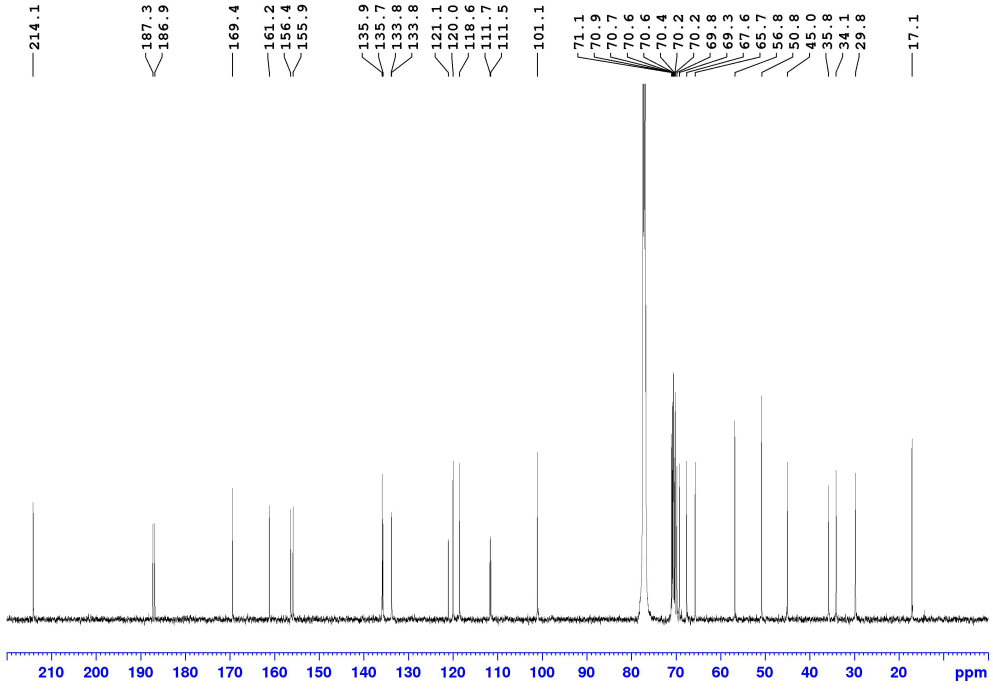

Dimethyl 3,3'-(((2-aminopyrimidine-4,6-diyl)bis(ethane-2,1-diyl))bis(sulfanediyl))(2*R*,2'*R*)-bis(2-((*tert*-butoxycarbonyl)amino)propanoate) (28)

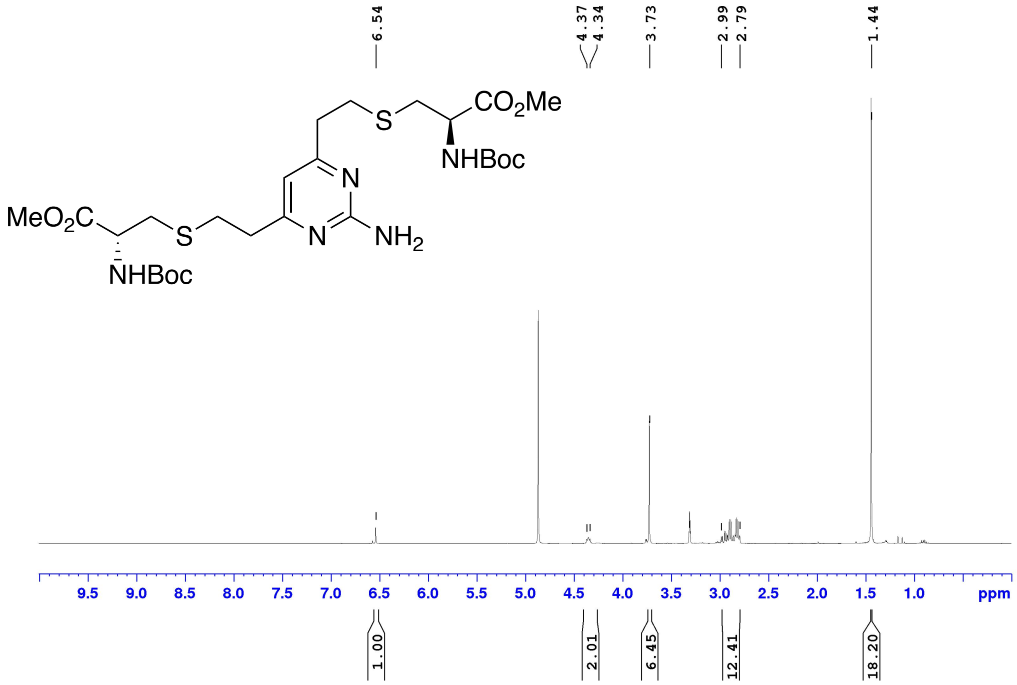

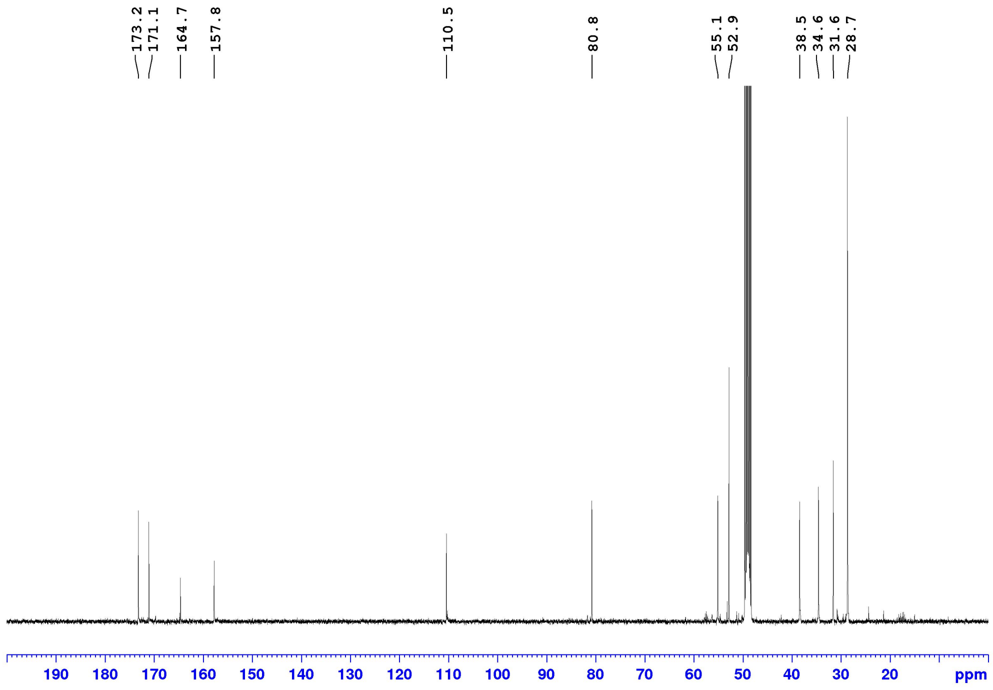

Dimethyl 3,3'-(((2-(hex-5-yn-1-ylamino)pyrimidine-4,6-diyl)bis(ethane-2,1-diyl))bis(sulfanediyl))(2*R*,2'*R*)-bis(2-((*tert*-butoxycarbonyl)amino)propanoate) (29)

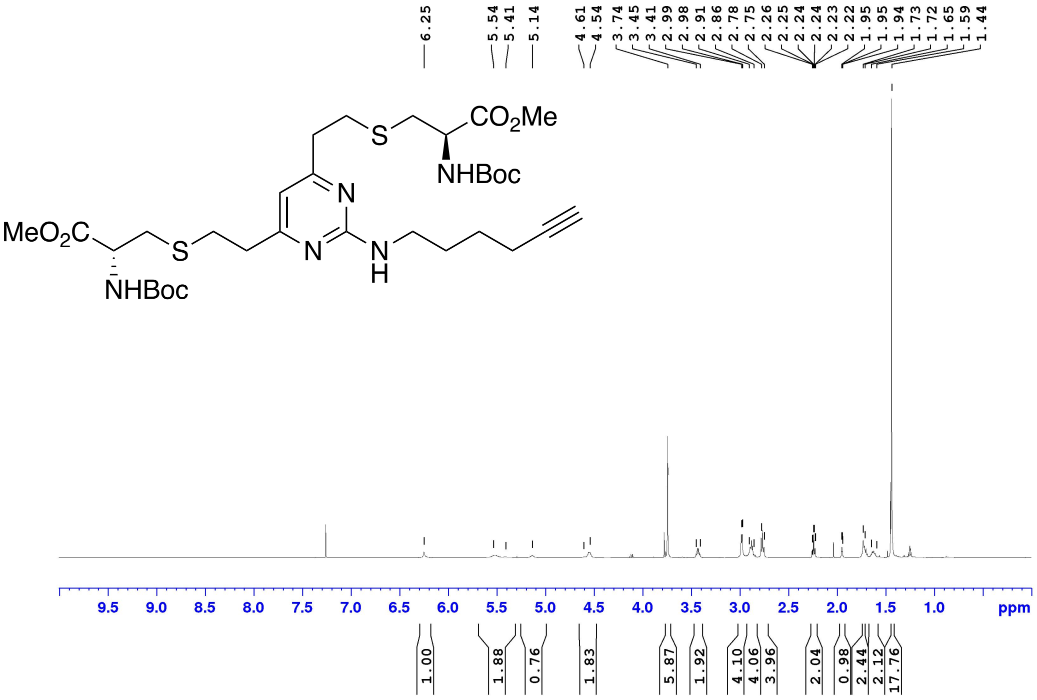

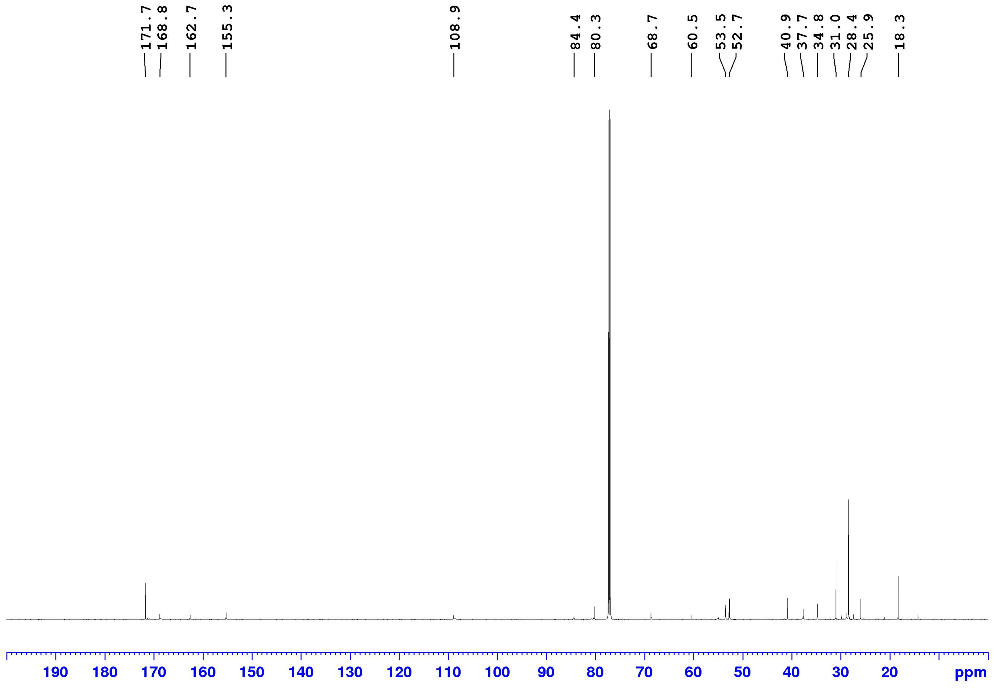

_N_-(4,6-bis(2-(((_R_)-2-((_tert_-butoxycarbonyl)amino)-3-methoxy-3-oxopropyl)thio)ethyl)pyrimidin-2-yl)-_N_-methylglycine (30)

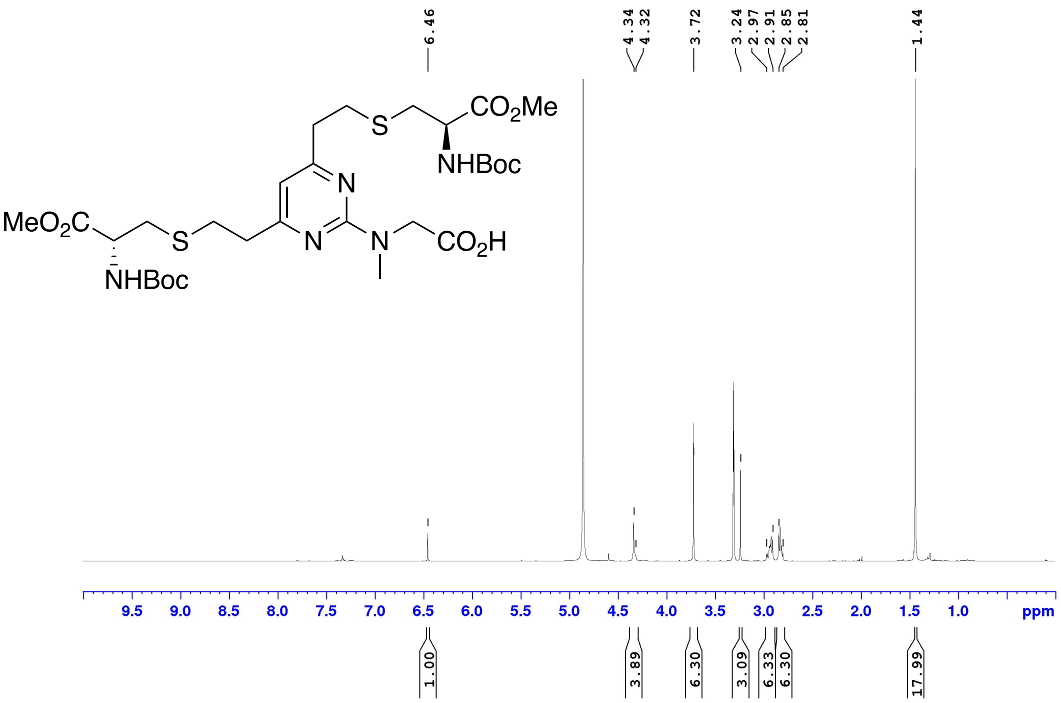

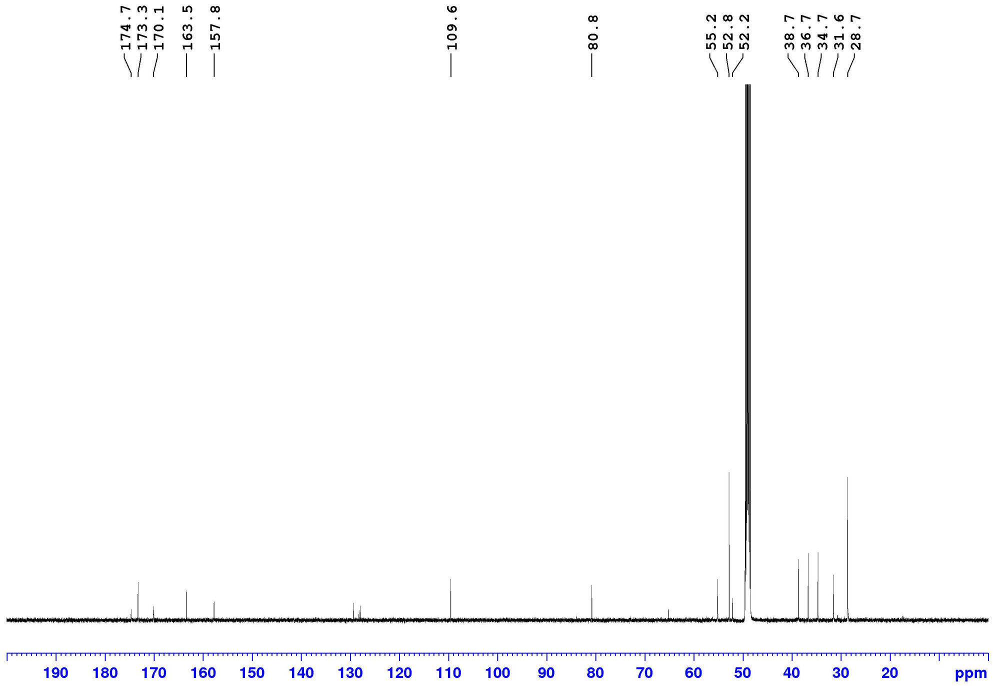

HPLC

N_3-PEG$_4$-MMAE (23)

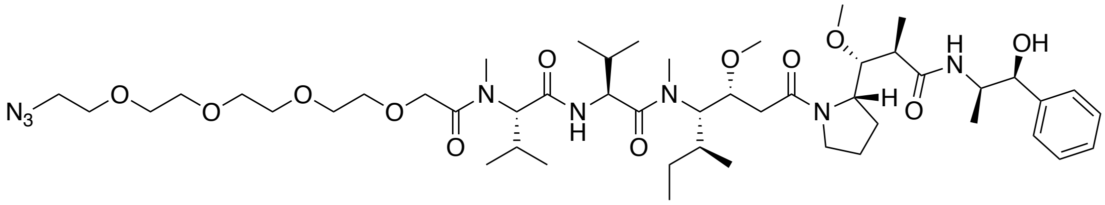

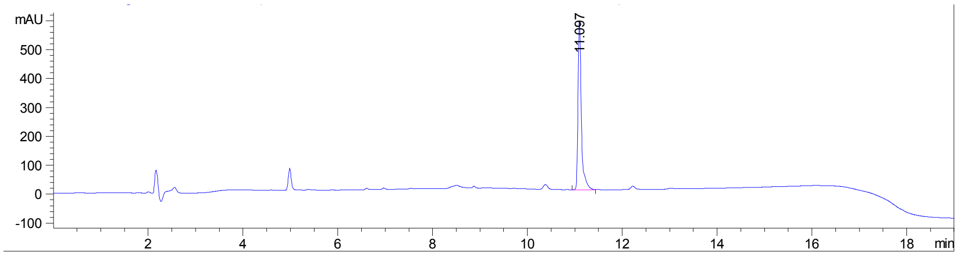

DVP-PEG$_4$-MMAE (24)

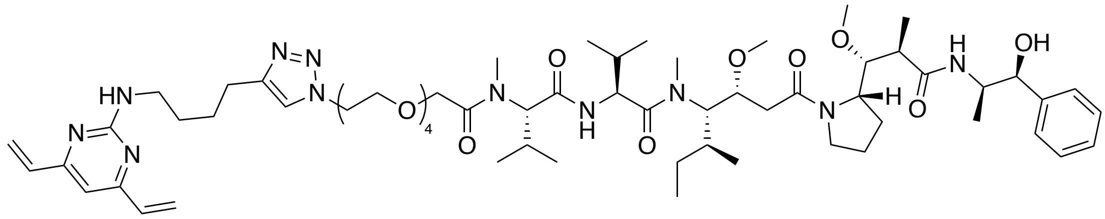

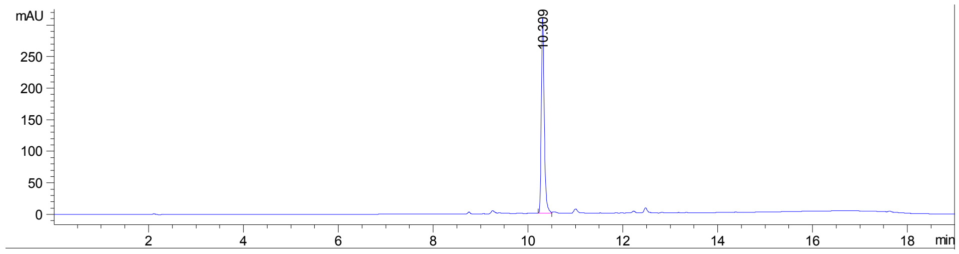

Supplement: Supplementary file 1 [file SC-010-C8SC04645J-s001.pdf]
